# Development and Properties of *Francisella tularensis* Subsp. *holarctica* 15 NIIEG Vaccine Strain without the *recD* Gene

**DOI:** 10.3390/vaccines10010108

**Published:** 2022-01-11

**Authors:** Vitaly Pavlov, Galina Vakhrameeva, Alexander Mokrievich, Mikhail E. Platonov, Galina Titareva, Raisa Mironova, Tatiana Kombarova, Tatiana Gapelchenkova, Ivan Dyatlov

**Affiliations:** State Research Center for Applied Microbiology and Biotechnology (SRCAMB), 142279 Obolensk, Russia; vahrameeva@obolensk.org (G.V.); mokrievich@obolensk.org (A.M.); platonov@obolensk.org (M.E.P.); titareva@obolensk.org (G.T.); mironovari@obolensk.org (R.M.); kombarova@obolensk.org (T.K.); gapelchenkova@obolensk.org (T.G.); dyatlov@obolensk.org (I.D.)

**Keywords:** *Francisella tularensis* 15 NIIEG, *recD* gene, allelic exchange, in vitro and in vivo reproduction, BALB/c mice

## Abstract

The genomic analysis of all subspecies *F. tularensis*, as found in Gen Bank NCBI, reveals the presence of genes encoding proteins like to the multifunctional RecBCD enzyme complex in *E. coli* and other bacteria. To date, the role of the *recD* gene in *F. tularensis*, which encodes the alpha chain of exonuclease V, in DNA metabolism processes, has not been studied either in vitro or in vivo. *F. tularensis* subsp. *holarctica* 15 NIIEG, a vaccine strain, served as the basis to create the *F. tularensis* 15D strain with *recD* deletion. The lack of the *recD* gene suppresses the integration of suicide plasmids with *F. tularensis* genome fragments into the chromosome. The modified strain showed reduced growth in vitro and in vivo. This study shows that such deletion significantly reduces the virulence of the strain in BALB/c mice.

## 1. Introduction

Tularemia is an acute infectious disease in humans and animals that is etiologically caused by the bacteria *Francisella tularensis*; humans’ high susceptibility to the pathogen makes this disease extremely dangerous for subspecies of *F. tularensis* subsp. *tularensis* (mortality rate of up to 60% in untreated cases) [1,2]. To prevent tularemia, endemic areas of Russia use a live vaccine based on the *F. tularensis* subsp. *holarctica* 15 NIIEG strain [3]. In Western Europe and North America, they use a live vaccine based on an *F. tularensis* LVS (live vaccine strain) in case of an emergency, which is obtained by a series of passages of *F. tularensis* 15 in vitro [4]. Both vaccine strains of *F. tularensis* have several weaknesses. The vaccine based on the 15 NIIEG strain causes adverse reactions in 6–10% of vaccinated patients [5]. The LVS strain has the same shortcomings: besides reactogenicity, the culture exhibits some dissociation during culturing [6,7]. All of this prevents the FDA from licensing a live tularemia vaccine in the USA [8].

Homologous recombination involving products of the *recA* gene and the multifunctional RecBCD enzyme complex is one of the factors affecting the stability of the bacterial genome [9]. The genomes of the genus Francisella contain regions encoding the RecBCD enzyme complex like to the structure of the region encoding the *E. coli* RecBCD enzyme complex. The amino acid sequence of the *E. coli* RecD protein has 40% similarity and 27% identity compared to the sequence of the RecD protein of *F. tularensis* subsp. *holarctica*. In the LVS and 15 NIIEG strains, the *recD* gene forms a single operon with the *recB (FTL_0669)* gene; genes *recBD* and *recC(FTL_0666)* are separated by an intergenic region of 71 bp [Gen Bank NCBI, CP009694.1] (Supplementary S1).

The genomes of all *F. tularensis* subspecies have a *recD*-like nucleotide sequences: *FTL_0670* [Gen Bank NCBI, CP009694.1] in *holarctica*, *FTM_0631* [Gen Bank NCBI, CP000915.1] in *mediasiatica*, [Gen Bank NCBI, AJ749949.2] in *tularensis.*

The role of *recD* in *F. tularensis* in DNA metabolism and reproduction in vitro and in vivo remains unclear. Here, we describe the construction of *F. tularensis* 15 NIIEG strain without *recD* gene and further research of its properties. *recD* deletion significantly reduces the ability to integrate suicide plasmids with *F. tularensis* genome fragments and the bacteria growth in vitro. Complementation of the deletion restores the molecular and biological properties of the modified strain.

## 2. Materials and Methods

*Bacterial strains, plasmids, and primers.* Bacterial strains and plasmids used in this study are listed in Table 1. The primers for synthesis and control of the modified regions of the *F. tularensis* genome are listed in Table 2.

*Culture conditions. F. tularensis* strains were grown at 37 °C in FT agar: per 1 L: fish meal hydrolysate 16 g, enzymatic lysate of cattle blood 4 g, a mixture of B vitamins 0.014 g, calcium pantothenate 0.014 g, magnesium sulfate 0.45 g, sodium sulphite 0.65 g, cysteine 0.4 g, glucose 6 g, agar 10 g, pH 7.2 (manufactured by the department of nutrient media SRCAMB), chocolate agar [18], or in brain heart infusion broth (BHI) (Becton–Dickinson, Franklin Lakes, NJ, USA), and FTB broth: per one l–5 g of casein acid hydrolysate, 5 g of yeast extraction, 12 g of monopotassium phosphate, 3.9 g of potassium hydroxide, 10 g of sodium chloride, 0.1 g of cysteine hydrochloride monohydrate, 6 mg of iron II sulfate heptahydrate, 2 g of glucose, pH 7.2. The cultivation was carried out in flasks on a thermostated shaker, 200 rpm, or in the plate wells on the shaker Multiscan FC unit (Thermo scientific, Singapore). When required, polymyxin B (100 μg/mL), chloramphenicol (3 μg/mL), kanamycin (20 μg/mL), or sucrose (10%) was added to media. Bacterial inocula for animal infection studies was generated by streaking *F. tularensis* strains onto FT plates, resuspending bacteria in PBS, and diluting to the appropriate concentration.

*E. coli* was grown at 37 °C in Luria–Bertani broth (LB) and on LB agar (LA) [19]. LB was supplemented with ampicillin (100 μg/mL), chloramphenicol (20 μg/mL), kanamycin (20 μg/mL), or sucrose (10%) when required.

For growth curves, single colonies of 15 NIIEG and the derivates were grown on FT-agar plates, then prepared a bacterial suspension in BHI broth to an optical density at 600 nm (OD_600_) of 0.8. Ten microliters of each culture was added to wells with 190 microliters of BHI broth. Moreover, 96-well culture flat bottom plate with a lid (Costar REF 3599, Corning, NY, USA) was used for cultivation in Multiscan FC unit (Thermo scientific) at 37 °C with shaking 100 rpm/min for 24 h. OD_600_ was recorded in wells at one-hour intervals.

*DNA manipulations.* To isolate plasmid DNA from *E. coli* cells, plasmid-transform *E. coli* cells, and perform genetic engineering, we followed the guidelines in [20]. Plasmid DNA from *F. tularensis* was isolated as described earlier in [21]. *F. tularensis* DNA was isolated with the GenElute™ Bacterial Genomic DNA Kit (Sigma-Aldrich, Moscow, Russia). *F. tularensis* whole-genome sequencing was performed using the Illumina MiSeq instrument according to the manufacturer’s instruction. MiSeq Reagent Kit v3 (300 cycles) was used for sequencing.

*Interspecies conjugation.* Plasmids were transferred from *E. coli* S17-1 cells into *F. tularensis* cells as described in [11,22] and modified as follows: 50 μL of a suspension of the donor *E. coli* S17-1 strain with the mobilized plasmid (1 × 10^8^ cells) was mixed with 50 μL of the recipient *F. tularensis* strain (3 × 10^10^ cells), and was spotted 10 μL on LB agar. The cultures were then incubated at 25 °C over 20 h and seeded onto FT agar containing 100 μg/mL of polymyxin B and 1 μg/mL of chloramphenicol to select *F. tularensis* clones with the *cat* gene of plasmid. Petri dishes were incubated 5 days at 37 °C.

*Cryotransformation of F. tularensis bacteria by plasmid DNA.* Overnight agar culture was suspended in 10 mL of FTB.: The suspension was cultivated in a shaker at 37 °C and 200 rpm over 2.5 h and concentrated by centrifugation. Centrifugation sediment was suspended in 80 μL of the transformation buffer TB (0.1 M MgSO_4_, 10 mM (NH_4_)_2_ SO_4_, pH 7.5).

For cryotransformation, 2 μL of the DNA solution was mixed with 20 μL of the bacterial suspension in a 250-μL microtube, and incubated over 10 min at room temperature. Then the tube was cooled down in liquid nitrogen over 5 min and heated to 37 °C over 10 min. The suspension was inoculated into 500 μL FTB, incubated at 37 °C over 2 h and plated onto the selective FT agar containing selective antibiotic [23].

*Design of pHVmob plasmid.* pHV33 plasmid DNA was digested with BamHI and ligated with 1.7-kb Bam HI fragment with a *mob* region of pPV plasmid [11]. Recombinant plasmid was transformed into *E. coli* DH5α. The clones with *~*8.9-kb pHVmob plasmid were selected on LA with chloramphenicol. Functional activity of the *mob* region in pHVmob was tested by its ability to mobilize the plasmid from *E. coli* S17-1λpir into *F. tularensis* 15 NIIEG.

*Design of bireplicon vector pUK194.* pUK194 with *amp* and *kan* genes was constructed as result of ligation of plasmids pK194 and pUC19 digested with HindIII.

pK194 was developed on base of modified pHV33 plasmid carrying *kan* gene from mTn*10*Km transposon instead *cat* gene. pHV33Km was selected after insertion mTn*10*Km transposon in *F. tularensis* 15(pHV33). pHV33Km was digested with HindIII, 2.9-kb fragment gel purified and ligated. Plasmid (pK194) obtained contains *kan* gene and is capable of autonomously replication in *F. tularensis* 15 NIIEG.

recD deletion in F. tularensis 15 NIIEG genome.

**Figure 1:**
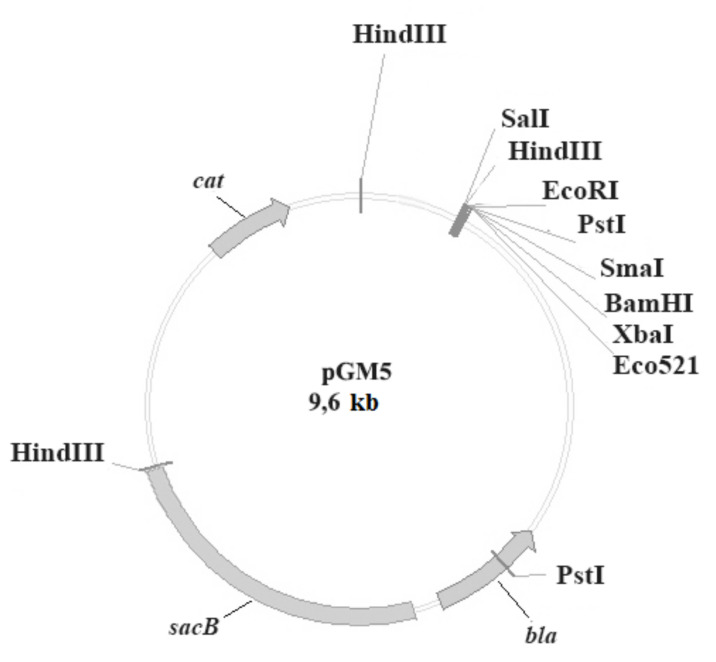
pGM5 plasmid.

*Design of suicide plasmid for recD deletion.* pGMΔrecD plasmid was create on base of pGM5 vector [16] (Figure 1).

FSD/RBD and FBD/RSD primers were used for synthesis of two amplicons adjacent to the *recD F. tularensis* gene and sized 1433 bp (5′ left arm) and 1462 bp (3′ right arm). The amplicons were hydrolyzed with SalI and BamHI and ligated with SalI linearized and dephosphorylated pGM5. The mixture after ligation was transformed the *E. coli* DH5α. Clones with pGMΔrecD plasmid were selected on Amp^R^Cm^R^Suc^S^ phenotype and by PCR on the FCD/RCD primers.

*Allelic replacement of recD gene.* Broth culture of *F. tularensis* with integrated pGMΔrecD was incubated at 37 °C over 6 h, then plated onto FT agar with added 5% sucrose and polymyxin B and continued incubation at 37 °C over 3 days for selection of Suc^R^ clones. The clones were checked on Cm^S^ phenotype on FT agar with Cm (1 μg/mL). and presence only genome region without *recD* gene using FCD/RCD and FSD/RSD primers.

*Creation of plasmid with recD gene (pUK/recD) for complementation.* A 3498-kb amplicon with the *recD* gene was created by PCR on FSD/RSD primers and *F. tularensis* 15 NIIEG DNA, hydrolyzed with SalI and ligated in SalI site of pUK194. The ligate was used to transform *E. coli* DH5α cells. Presence of *F. tularensis recD* gene in Amp^R^Km^R^ transformants was checked by PCR on FSD/RSD and FCD/RCD primers.

*Design of suicide plasmid for sodC deletion.* pGMΔsodC plasmid was create on base of pGM5. sodCL-F/sodCL and sodCR-F/sodCR-R primers were used for synthesis of two amplicons adjacent to the *sodC* *F. tularensis* gene and sized 937 bp (5′ left arm) and 745 bp (3′ right arm). The amplicons were hydrolyzed with SalI and BamHI and ligated with SalI linearized and dephosphorylated pGM5. The mixture after ligation was transformed the *E. coli* DH5α. Clones with pGMΔsodC plasmid were selected on Amp^R^Cm^R^Suc^S^ phenotype and by PCR on the sodC-KF/sodC-KR primers.

*Testing F. tularensis strains’ capacity for homologous recombination. F. tularensis* 15 NIIEG, 15D, 15D(pUK/recD, and 15D(pUK194) strains were tested for their capacity for homologous recombination of pPV/∆*iglC* [11] and pGMΔsodC suicide plasmids. With this purpose pPV/∆*iglC* was mobilized from *E. coli* S17-1 into *F. tularensis*, and pPV/∆*iglC* was cryotransformated into *F. tularensis*. pHVmob plasmid autonomous replicating in *F. tularensis* and capable of mobilizing from *E. coli* S17-1 into *F. tularensis* was used as control for efficiency of plasmids transfer.

*Infection of macrophages*. The murine macrophage line J774A.1 from the Russian Collection of Cell Cultures, St. Petersburg was used. 1 × 10^5^ cells were seeded into wells of in 24-well tissue culture plates (Costar, Corning, NY, USA) and incubated in Dulbecco’s modified Eagle’s medium (DMEM; GIBCO BRL, Grand Islands, NY, USA) supplemented with 10% fetal bovine serum and 2 mM L-glutamine at 37 °C in humidified air containing 5% CO_2_. 24 h. *F. tularensis* suspension was inoculated in the wells with macrophages monolayer at a multiplicity of infection 100:1. After incubation for 2 h at 37 °C, wells were washed with PBS to remove extracellular bacteria. Macrophages were reconstituted in culture medium supplemented with 2 μg/mL of gentamicin and incubated for the indicated periods of time. Then, the infected macrophages were lysed with 0.5 mL of 0.05% dodeoxycholate and the number of CFU determined by plating serial 10-fold dilutions on FT agar medium.

*Animal infections.* All animal studies were conducted at the Protocol Number VP-2021/4 from 23.09.2021 of the SRCAMB Bioethics Committee in accordance with the Good Animal Care requirements [24]. Six- to 8-week-old BALB/c mice (Institute of Bioorganic Chemistry, Russian Academy Sciences in Pushchino, Russian Federation) were infected subcutaneously (s.c.) in a final volume of 0.2 mL with *F. tularensis* 15 NIIEG derivatives. Challenge doses per mouse were from 1 × 10^2^ to 1 × 10^5^ CFU (five mice per one dose).

*Statistical Analyses.* All values were expressed as means ± standard errors of the means. One-way analysis of variance (ANOVA), followed by a post hoc test (Bonferroni) and a one-tailed Student *t* test, was used to identify differences between groups.

## 3. Results and Discussion

### 3.1. Comparative Analysis of Amino Acid Sequences RecD Proteins in the Genus Francisella

The amino acid sequences of in strains of different species and subspecies of the genus Francisella have some differences. RecD protein *F. philomiragia* and *F. tularensis* subsp. *novicida*, in comparison with other subspecies of the tularemia microbe, has an extended deletion of 22 aa. At the N-terminus of the RecD protein in *F. tularensis* subsp. *holarctica* and *F. philomiragia*, there is a 9 amino acid deletion, which distinguishes these species from others. There are 28 amino acid substitutions and a deletion of 22 amino acid residues in the RecD protein of *F. tularensis* subsp. *novicida* in comparison with other subspecies of the tularemia microbe. The amino acid sequences of the RecD proteins of *F. philomiragia* differ in 47 amino acid residues from RecD of subspecies of *F. tularensis* (Supplementary S3).

### 3.2. recD Deletion in F. tularensis 15 NIIEG Genome

**Figure 2:**
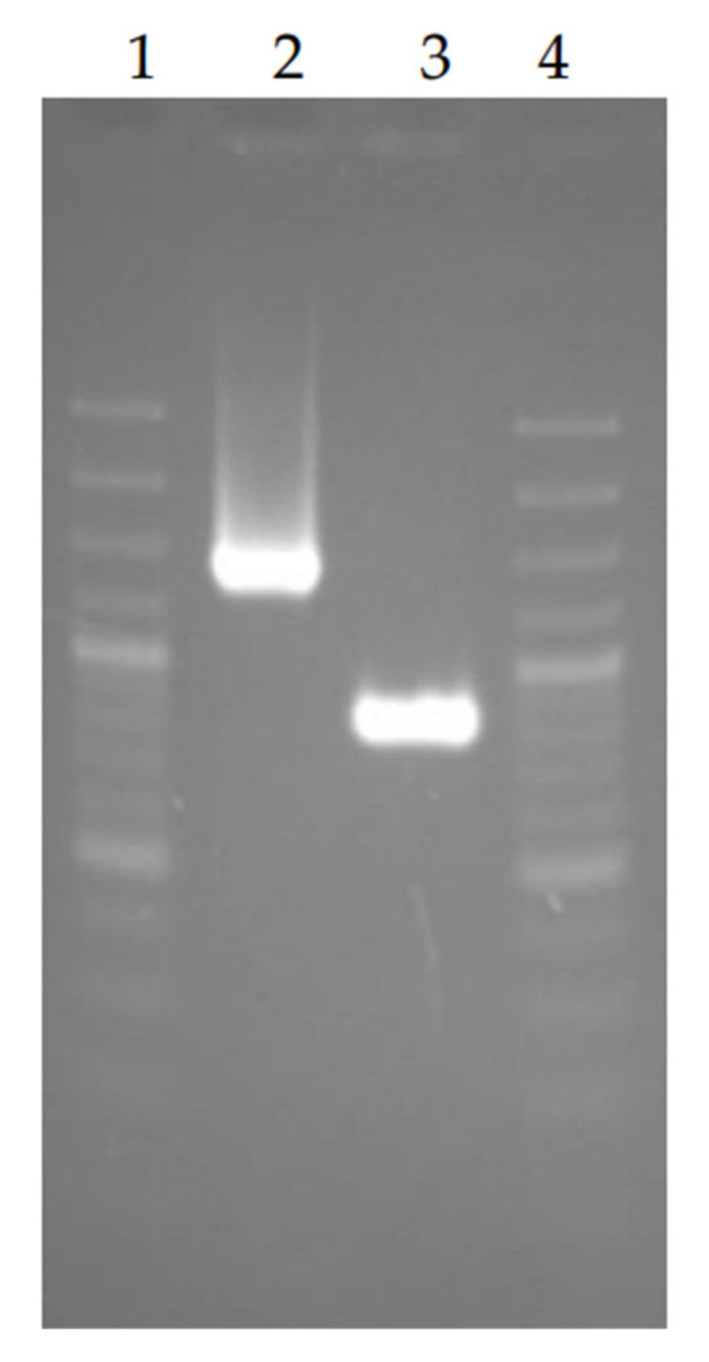
PCR analysis with FCD/RCD primers of *F. tularensis* DNA: 15 NIIEG—line 2, 15D—line 3. 100 bp plusDNA ladder (Fermentas)–lines 1 and 4.

The recD deletion variant of *F. tularensis* 15 NIIEG was obtained using the suicide plasmid pGMΔrecD, created on the basis of the pGM5 vector, consisting of the pBR22 plasmid and the modified pC194 plasmid, which lost the ability to replicate in *F. tularensis* [16]. Plasmid pGMΔrecD was introduced into *F. tularensis* 15 NIIEG cells by cryotransformation. Clones carrying pGMΔrecD were selected on FT-agar with chloramphenicol (1 μg/ml). The presence of sacB gene in the clones was detected by the inhibition of culture growth on FT-agar with the addition of 10% sucrose. FCD/RCD primers were used for a check of presence of both native and modified recD gene in the clones. As a result of allelic exchange, clones with the Cm^S^Suc^R^ phenotype were selected, containing only a modified region of the genome without the recD gene. Amplicon obtained with FCD/RCD primers on the modified strain 15D DNA was 0.6 kb shorter than it on parent strain DNA (Figure 2, Supplementary S4).

*F. tularensis* 15D was free from selection markers of pGM∆recD suicide plasmid, such as chloramphenicol resistance or sucrose sensitivity. Comparison of nucleotide sequences of *recD* regions of *F. tularensis* 15D and 15 NIIEG strains showed that the modified strain to have 604 nucleotides deletion. No nucleotide substitutions found in the *recB* and *recC* genes adjacent to *recD*, see Supplementary S5.

### 3.3. Effects of recD on Homologous Recombination in F. tularensis

To study the effects of the *recD* gene on homologous recombination in *F. tularensis*, we tested the *F. tularensis* 15D strain and its derivatives: 15D(pUK/recD) with pUK/recD plasmid carrying genome fragment with the *recD* gene; and 15D(pUK194) with pUK194 vector plasmid. The homologous recombination event was assessed by the appearance of clones resistant to chloramphenicol as a result of transfer of suicide plasmids carrying *F. tularensis* DNA fragments. pPVΔiglC plasmid integration efficiency into the *F. tularensis* 15D and 15D(pUK194) chromosomes by mobilization from *E. coli* S17-1 was significantly lower than it for parent strain 15 NIIEG, see Table 3. The same result was obtained for pGMΔsodC plasmid which was transformed *F. tularensis* 15D and 15D(pUK194). The phenomenon of homologous recombination with the participation of the suicide plasmids in strain 15 NIIEG was confirmed by the fact that the transfer of vectors (pPV and pGM5) did not lead to the appearance of Cm^R^ clones. Transformation of pUK/recD plasmid into the 15D strain restored the suicide plasmids integration capacity to the same level of efficiency as in the parent strain 15 NIIEG, see Table 3.

The modified strains practically did not differ from 15 NIIEG in their ability to mobilize (~1 × 10^7^ CFU) and transform (~1 × 10^6^ CFU per μg of DNA) in experiments with the pHVmob plasmid that replicating autonomously into *F. tularensis* (Table 4).

### 3.4. Growth of F. tularensis 15D in Nutrient Media and J774.1A Cells

**Figure 3:**
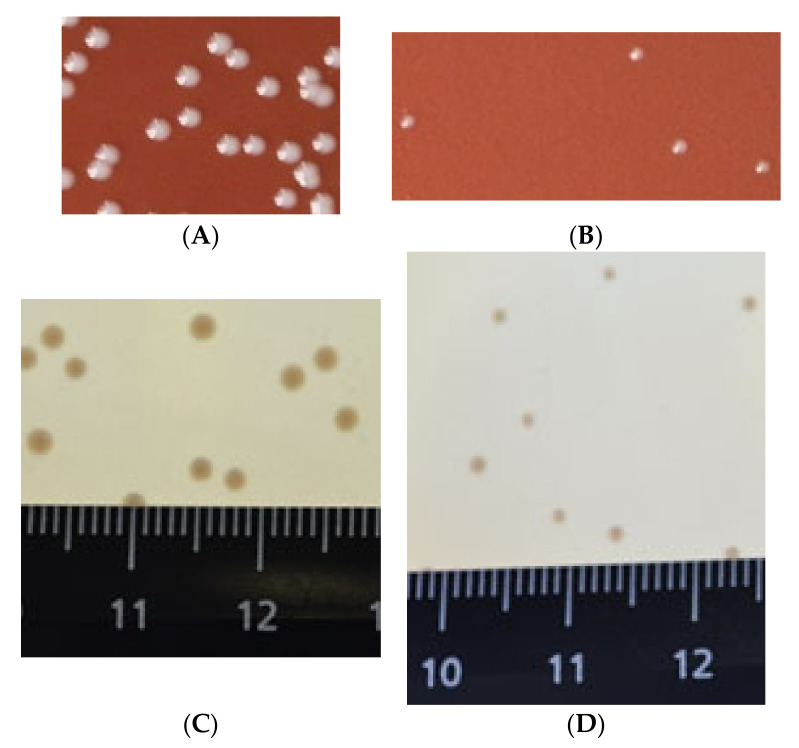
*F. tularensis* colonies morphology on chocolate agar (**A**,**B**), FT-agar (**C**,**D**): 15 NIIEG (**A**,**C**) and 15D (**B**,**D**).

Comparative analysis of *F. tularensis* 15D colonies sizes on chocolate agar after 3 days of culturing at 37 °C showed that modified strain colonies were smaller *F. tularensis* 15 NIIEG one, see Figure 3.

**Figure 4:**
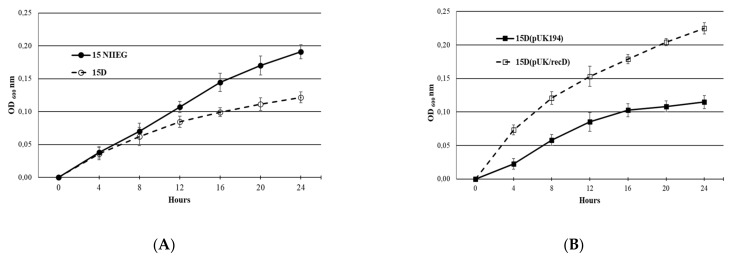
*F. tularensis* growth in BHI broth at 37 °C, strains: (**A**)—15D and 15 NIIEG; (**B**)—15D (pUK/recD) and 15D (pUK194).

Slower growth of *F. tularensis* 15D in comparisio with 15 NIIEG was observed in BHI medium, see Figure 4A. Transcomplementation of the *recD* deletion in *F. tularensis* 15D with pUK/recD restored speed growth in comparison to the 15D(pUK194), carrying only plasmid vector, see Figure 4B.

**Figure 5:**
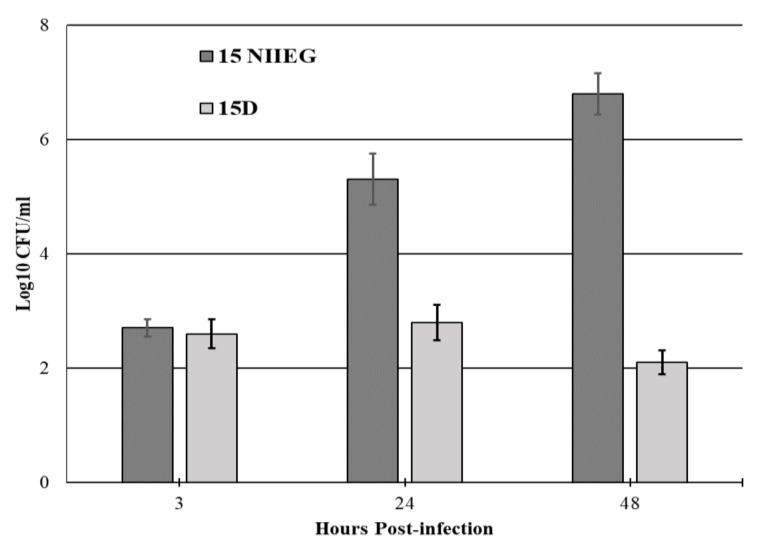
Effect of *recD* deletion on *F. tularensis* reproduction in J774.1A cells. 15 NIIEG strain was used as control. Macrophages were infected with bacteria at a ratio of 100:1. The macrophages were lysated at 3, 24, and 48 h upon infection. The diagram shows the average results of the number of colonies on FT agar as Lg CFU/mL ± confidence interval (*p* < 0.05) obtained in three independent experiments.

*F. tularensis* 15D bacteria, aside from reduced growth in vitro, had reduced reproduction ability in J774.1A cells compared to the 15 NIIEG strain, see Figure 5.

### 3.5. recD Deletion Effects on F. tularensis Virulence

*F. tularensis* 15D without *recD* gene had significantly reduced virulence for BALB/c mice infected s.c. in comparison with 15 NIIEG, see Table 5.

Transcomplementation of the *recD* deletion in *F. tularensis* 15D with pUK/recD increased virulence in comparison to the 15D and 15D (pUK194), carrying only plasmid vector, see Table 5.

## 4. Discussion

One of the possible directions for stabilizing and reactogenicity reducing of existing tularemia vaccines is the modification or deletion of genes, the products of which are involved in repair processes and intragenomic rearrangements. Such targets can be *recA* gene [25], *recB* [26], and *recD* gene. The function of RecD protein of *E. coli*, a component multifunctional enzyme RecBCD in nucleic acid exchange, has been studied in detail [27,28]. The *F. tularensis* LVS genome contains the FTL_0670 gene (Gen Bank NCBI) encoding a protein similar to the *E. coli* RecD protein. An identical nucleotide sequence is also present in the genome of *F. tularensis* 15 NIIEG (Supplementary S2).

*recD* gene deletion in strain 15 NIIEG led to a slowdown in bacterial growth on solid and liquid nutrient media, as well as to a slowdown in reproduction in J774.1A macrophage-like cells. The observed decrease in the replication of strain 15D in macrophages can probably be explained by its reduced ability to grow in the BHI medium, which mimics the growth conditions in macrophages [29,30,31]. This effect may have led to a significant decrease in the virulence of the modified *F. tularensis* 15D strain. Influence of the *recD* gene on bacterial growth was previously demonstrated for *Pseudomonas syringae* Lz4W [32].

There are extended nucleotide repeats in *F. tularensis* genomes [33], which could be the reason for genomic rearrangements. To elucidate the *recD* gene role in the process of homologous recombination, the *F. tularensis* 15 NIIEG strain without the *recD* gene was created. The recipient properties of 15D strain during interspecies transfer and cryotransformation of autonomous replication in *F. tularensis* plasmids did not differ from the properties of the parental strain. However, the transfer and integration of suicide plasmids carrying extended *F. tularensis* genome fragments into 15D strain were significantly suppressed in comparison with strain 15 NIIEG. Transcomplementation of this mutation in 15D strain led to the restoration of suicidal plasmids integration. Thus, the deletion of the *recD* gene may reduce the likelihood of intragenomic rearrangements in the vaccine strain, which will lead to stabilization of the properties of the tularemia vaccine. The properties of the vaccine strain without the *recD* gene listed above allow us to hope that this approach is promising for the development of more stable and less reactogenic vaccine strains of *F. tularensis*.

## Figures and Tables

**Table 1:** Bacterial strains and plasmids used in this research.

Name	Description	Source or Reference
Strains		
*E. coli* DH5α	*F^-^ (φ80dlacZΔM15) recA1 endA1 gyrA96 thi-1 hsdR17(r_k_^–^m_k_^+^) supE44 relA1 deoR Δ(lacZYA-argF) U169*	GKPM-Obolensk * [10]
*E. coli* S17-1 λpir	(*thi pro hsdR– hsdM+ recA* RP4-2-Tc::Mu-Km::Tn7(TpR SmR))	GKRM-Obolensk [11]
*E. coli* S17(pHVmob)	Amp^R^, Cm^R^, a derivative of S17-1 *λpir* containing pHVmob plasmid	This paper
*E. coli* DH5α(pGM∆recD)	Amp^R^, Cm^R^, Suc^S^, derivative of DH5α, pGM∆recD plasmid	This paper
*E. coli* DH5α (pUK194)	Amp^R^ Km^R^ derivative of DH5α containing pUK194 plasmid	This paper
*E. coli* DH5α(pUK/recD)	Amp^R^ Km^R^ derivative of DH5α with pUK194 plasmid, a fragment of the *F. tularensis* genome with the *recD* gene	This paper
*E. coli* DH5α(pGM∆sodC)	Amp^R^, Cm^R^, Suc^S^, derivative of DH5α containing pGM∆sodC plasmid	This paper
*E. coli* S17(pPV∆glC)	Amp^R^, Cm^R^, Suc^S^, derivative of S17-1 *λpir* containing pPV∆iglC plasmid,	GKRM-Obolensk [11]
*F. tularensis* 15 NIIEG	Pm^R^, Amp^R^, subsp. *holarctica*, a vaccine strain	GKPM-Obolensk
*F. tularensis* 15D	Pm^R^, a modified *F. tularensis* 15 NIIEG strain with the *recD*gene deleted	This paper
*F. tularensis* 15D(pHVmob)	Pm^R^, Cm^R^ *F. tularensis* 15D with pHVmob plasmid	This paper
*F. tularensis* 15(pK194)	Km^R^, a *F. tularensis* 15 NIIEG strain with pK194 plasmid,	This paper
*F. tularensis* 15D(pUK194)	Pm^R^, a *F. tularensis* 15D strain with pUK194 plasmid	This paper
*F. tularensis* 15D(pUK/recD)	Pm^R^, a *F. tularensis* 15D with pUK/recD plasmid containing a fragment of the *F. tularensis* genome with the *recD* gene	This paper
Plasmids		
pC194	Cm^R^. Replicates autonomously in *F. tularensis* cells	[12,13,14]
pHV33	Amp^R^, Cm^R^	[15]
pHVmob	Amp^R^, Cm^R^, pHV33 plasmid containing a 1.7-kb fragment of RP4 plasmid with a *mob* region	This paper
pGM5	Amp^R^, Cm^R^, sacB.	[16]
pGM∆recD	Amp^R^, Cm^R^, sacB, pGM5 plasmid with a modified 2.9-kb fragment of the F. tularensis 15 NIIEG genome, 603 bps deleted from the recD gene	This paper
pK194	Km^R^, derivative of pC194 plasmid with the *cat* gene replaced by Km^R^ from the mTn*10*Km transposon. Replicates autonomously in *F. tularensis* cells	This paper
pUC19	Amp^R^	[17]
pUK194	Amp^R^ Km^R^ cointegrate of pUC19 and pK194 plasmids at the HindIII site.	This paper
pUK/*recD*	Amp^R^ Km^R^, pUK194 plasmid with a 3.5-kb fragment of the *F. tularensis* genome containing the *recD* gene	This paper
pPV∆iglC	Amp^R^, Cm^R^, *sacB*, pPV plasmid with a 3.0-kb fragment of the *F. tularensis* 15 NIIEG genome, 545 bps deleted from the *iglC* gene	[11]
pGM∆sodC	Amp^R^, Cm^R^, *sacB*, pGM5 plasmid with a modified 1.7-kb fragment of the F. tularensis 15 NIIEG genome, 528 bps deleted from the *sodC* gene	This paper

* GKPM-Obolensk stands for the State Collection of Pathogenic Microorganisms and Cell.

**Table 2:** Primers used in this research.

Name	Sequence 5′-3′ ^a,b^	Primer Localization
For *recD* deletion		
Amplicon upstream the *recD* gene		
FSD	AAAgtcgacTGGCAAAGATGATAGTGT	forward primer with a SalI site
RBD	AAAggatccTTACATAGACGGAGTAGTCT	reverse primer with a BamHI site
Amplicon downstream the *recD* gene		
FBD	AAAggatccTAAGCTCAGAGAATGACAGA	forward primer with a BamHI site
RSD	AAAgtcgacGTGCTATCCTACCAGG	reverse primer with a SalI site
Amplicon to control *recD* deletion		
FCD	GATGGTTATGGAGTTTATCGAGC	forward primer
RCD	TAAGAGCCTCTTTGTAGTCACGG	reverse primer
For *sodC* deletion		
Amplicon upstream the *sodC* gene		
sodCL-F	AAAgtcgacAACGACAGCATATTGCCACTCATAG	forward primer with a SalI site
sodCL-R	AAAggatccCACCTCCAAAATTTAGGTCATATC	reverse primer with a BamHI site
Amplicon downstream the *sodC* gene		
sodCR-F	AAAggatccGTGCTAGAATGTGGTGTGGAGTTA	forward primer with a BamHI site
sodCR-R	AAAgtcgacCATATCAATATGACCTTTCTTTGGC	reverse primer with a SalI site
Amplicon to control *sodC* deletion		
sodC-KF	CGTATCAGCTAAAGTGATAATCGGT	forward primer
sodC-KR	GACAAAATACTGCAACACCAACAGC	reverse primer

^a^ Primers for amplification of target fragments in the tularemia microbe chromosome designed using the *F. tularensis* ssp. *holarctica* LVS gene sequence (Gen Bank NCBI, CP009694.1). Comparative analysis showed the *recBCD* operons in LVS and 15 NIIEG strains to have identical nucleotide sequences, see Supplementary S2. ^b^ Lowercase refers to recognition sites for restriction enzymes.

**Table 3:** Efficiency of the integration of pPVΔiglC and pGMΔsodC suicide plasmids into *F. tularensis* chromosomes.

*F. Tularensis* Strain	Cm^R^ Clones Per Recipient Cell	
	pPV/∆iglC	pGM/∆sodC
15D	<1 × 10^−9^	<1 × 10^−9^
15 NIIEG	1 × 10^−7^	1 × 10^−6^
15D(pUK/recD)	1 × 10^−6^	1 × 10^−6^
15D(pUK194)	<1 × 10^−9^	<1 × 10^−9^

**Table 4:** Efficiency of the transfer of pHVmob plasmid into *F. tularensis* strains.

*F. Tularensis* Strain	Cm^R^ Clones Per Recipient Cell	
	Mobilization Efficiency (Per Recipient Cell)	Cryotransformation Efficiency (CFUμg DNA)
15D	3 × 10^−2^	1 × 10^6^
15 NIIEG	3 × 10^−2^	1 × 10^6^
15D (pUK/recD)	3 × 10^−2^	1 × 10^6^
15D (pUK194)	3 × 10^−2^	1 × 10^6^

**Table 5:** BALB/c mice survival after s.c. *F. tularensis* challenge.

*F. Tularensis* Strain	Challenge Dose	Survival (%)
15D	1 × 10^3^	100
	1 × 10^5^	100
15D(pUK/recD)	8 × 10^2^	0
	8 × 10^4^	0
15D (pUK194)	6 × 10^2^	100
	6 × 10^4^	100
15 NIIEG	1 × 10^2^	30
	1 × 10^3^	0

## Data Availability

All data used for this study are available in the text of the article and in the Supplementary Materials.

## Supplementary Materials

The following supporting information can be downloaded at: https://www.mdpi.com/article/10.3390/vaccines10010108/s1, Supplementary S1: Structure of the genome regions of *F. tularensis* and *E. coli* with *recBCD* genes; Supplementary S2: Comparison of the*recBCD* regions of *Francisella tularensis* subsp. *holarctica* LVS and *Francisella tularensis* subsp. *holarctica* 15 NIIEG; Supplementary S3: Multiple alignment of the amino acid sequence of the RecD proteins of the genus Francisella; Supplementary S4: Electrophoregram of amplicons; Supplementary S5: Comparison of the*recBCD* regions of *Francisella tularensis* subsp. *holarctica* LVS and *Francisella tularensis* subsp. *holarctica* 15D.
