# Supplementary material for: Development and Properties of Francisella tularensis Subsp. holarctica 15 NIIEG Vaccine Strain without the recD Gene
Source: Vaccines (Basel). 2022 Jan 11;10(1):108. doi: 10.3390/vaccines10010108 (PMC8778684; doi:10.3390/vaccines10010108)
Supplement: Supplementary file 1 [file vaccines-10-00108-s001.zip › Supplementary S1.pdf]

Supplementary 1.

Structure of the genome regions of *F. tularensis* and *E. coli* with *recBCD* genes

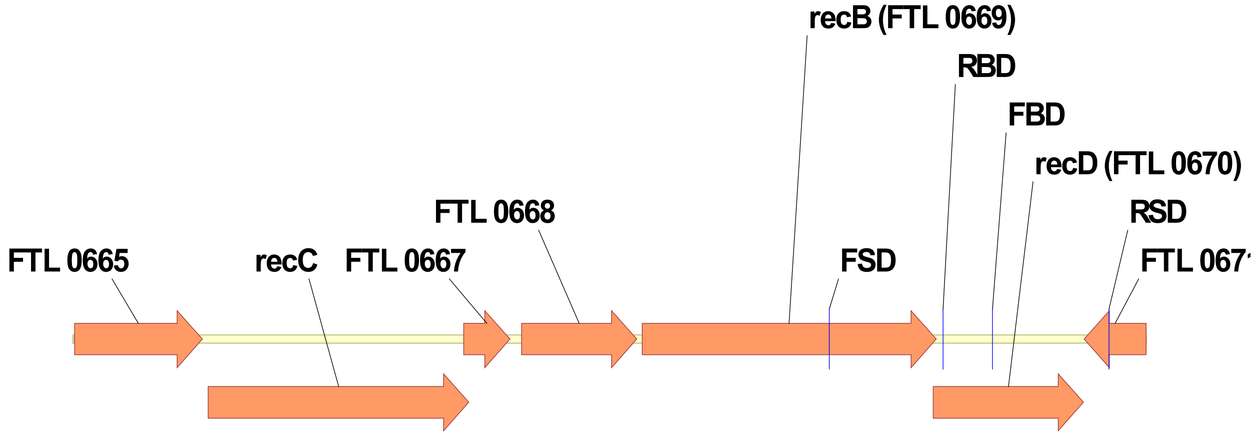

F tularensis LVS recBCD region

13328 bp

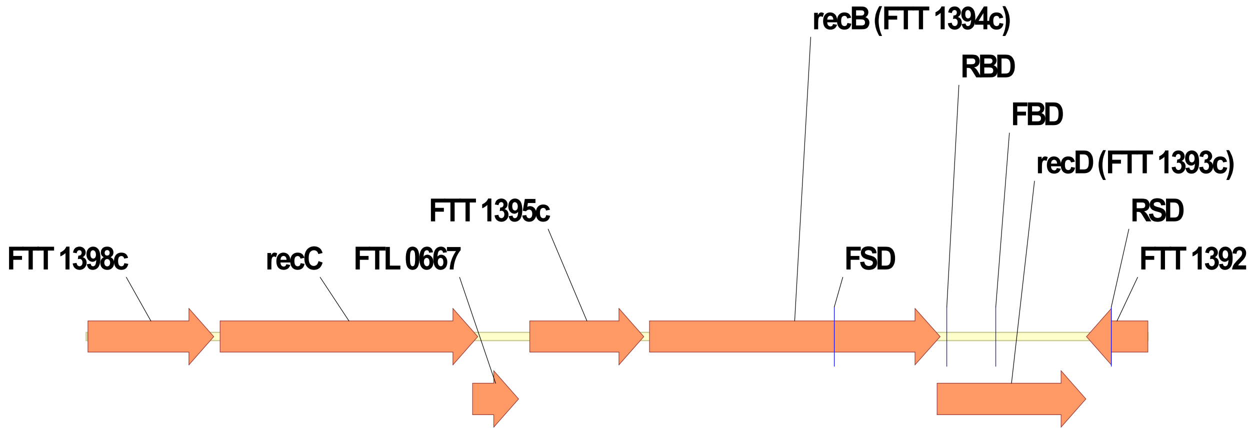

F tularensis SCHU S4 recBCD region

13327 bp

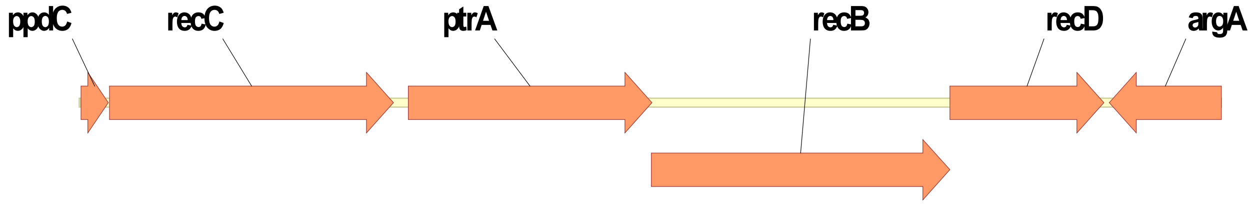

E coli K-12 MG1655 recBCD region

13523 bp

Multiple alignment of the amino acid sequence of the RecD proteins of *F. tularensis* and *E. coli*.

Strain	RecD protein accession number
E. coli K-12 MG1655	NP_417296.1
F. tularensis subsp. *holarctica* LVS	AJI59740.1

```
                               1                                               50
E_coli_K-12_MG1655_RecD    (1) --------------------------------------MKLQKQLLEAVE
  F_tularensis_LVS_RecD    (1) MLVYLMEMIMYKTFHKACEQLADIRAIDFFFAKEVFDLVKKTTPSMTTLS
              Consensus    (1)                                       MK     L  L
                               51                                             100
E_coli_K-12_MG1655_RecD   (13) HKQLRPLDVQFALTVAGDEHPAVTLAAALLSHDAGEGHVCLPLSRLENN-
  F_tularensis_LVS_RecD   (51) SESYPPFHGAKGNDLEQKSNEILFHILMKLMHVYSHGHSCLKIADISNKT
              Consensus   (51)      P     A  L       L      L H    GH CL IA I N
                               101                                            150
E_coli_K-12_MG1655_RecD   (62) -EASHPLLATCVSEIGELQNWEECLLASQAVSRGDEPTPMILCGDRLYLN
  F_tularensis_LVS_RecD  (101) IFASEQSNQSDAKVGFKMPSSDKIASILDNLNYDNLPIYFAKQYDSLYIK
              Consensus  (101)   AS     S       L   D        L     P        D LYI
                               151                                            200
E_coli_K-12_MG1655_RecD  (111) RMWCNERTVARFFNEVNHAIEVDEALLAQTLDKLFPVSDEINWQKVAAAV
  F_tularensis_LVS_RecD  (151) RLWNYENEIANFIKLKTIQNTQKDNRIEEVVDKLFEPAGEINWQKQAVIK
              Consensus  (151) RLW  E  IA F           D  I   LDKLF  A EINWQK A
                               201                                            250
E_coli_K-12_MG1655_RecD  (161) ALTRRISVISGGPGTGKTTTVAKLLAALIQMADGERCRIRLAAPTGKAAA
  F_tularensis_LVS_RecD  (201) SLNYNFSIISGGPGTGKTTTVAKLLLAIQMLNQ-NQQRIALLAPTGKAAQ
              Consensus  (201) AL    SIISGGPGTGKTTTVAKLL AI  L      RI L APTGKAA
                               251                                            300
E_coli_K-12_MG1655_RecD  (211) RLTESLGKALR-------------QLPLTDEQKK---------RIPEDAS
  F_tularensis_LVS_RecD  (250) RMTESLKNTLATVIPSSLTTESTIQKNDIFKQVQDELNSVAQYLNNLEAQ
              Consensus  (251) RLTESL   L              Q      Q               DA
                               301                                            350
E_coli_K-12_MG1655_RecD  (239) TLHRLLGAQPGSQRLRHHAGNPLHLDVLVVDEASMIDLPMMSRLIDALPD
  F_tularensis_LVS_RecD  (300) TIHRFLGLRPNSTYVKYNQQSKAPFDVIIVDEASMLDMNIFIKLIRAVAD
              Consensus  (301) TIHR LG  P S  LKH        DVIIVDEASMIDL I  KLI AL D
                               351                                            400
E_coli_K-12_MG1655_RecD  (289) HARVIFLGDRDQLASVEAGAVLGDICAYANAGFTAERARQLSRLTGTHVP
  F_tularensis_LVS_RecD  (350) NTKLILIGDTNQLPSVEAGSLLANFTHDKYG-------------------
              Consensus  (351)   KLI IGD  QL SVEAGALLA       A
                               401                                            450
E_coli_K-12_MG1655_RecD  (339) AGTGTEAASLRDSLCLLQKSYRFGSDSGIGQLAAAINRGDKTAVKTVFQQ
  F_tularensis_LVS_RecD  (381) --------SITPYTTLLIKNYRS--QQYINNLAASVLKGDVNIDS----Q
              Consensus  (401)         SI     LL K YR      I NLAAAI KGD         Q
                               451                                            500
E_coli_K-12_MG1655_RecD  (389) DFTDIEKRLLQSGEDYIAMLEEALAGYGRYLDLLQARAEPDLIIQAFNEY
  F_tularensis_LVS_RecD  (417) QNENIKFHSLRN-------LDIYLREYAKRYSQLEKCRDYKEALTELNKF
              Consensus  (451)     I    L         LD  L  YAK    L    D    I   N F
                               501                                            550
E_coli_K-12_MG1655_RecD  (439) QLLCALREGPFGVAGLNERIEQFMQQKRKIHRHPHSRWYEGRPVMIARND
  F_tularensis_LVS_RecD  (460) RILVANKNLEIGTDKLNQKIEKFMS-------KPIDSNYKGKPIMITQNS
              Consensus  (501)  IL A K    G   LN KIE FM         P    Y GKPIMI  N
                               551                                            600
E_coli_K-12_MG1655_RecD  (489) SALGLFNGDIGIALDRGQG-TRVWFAMPDGNIKSVQPSRLPEHETTWAMT
  F_tularensis_LVS_RecD  (503) YSLGLFNGDVGIMWPDDTGKLRAYFDGKDAKAFSLN--MLPKYESVYAMT
              Consensus  (551)  ALGLFNGDIGI      G  R WF   DA   SLN   LP HES WAMT
                               601                                            650
E_coli_K-12_MG1655_RecD  (538) VHKSQGSEFDHAALILPSQRTPVVTRELVYTAVTRARRRLSLYADERILS
  F_tularensis_LVS_RecD  (551) IHKTQGSEFDEIVIILPAEDNEALSKQLLYTAITRAKYKLTIISEQSSLR
              Consensus  (601) IHKSQGSEFD   IILPA     LSK LLYTAITRAK KLSI AD   L
                               651                672
E_coli_K-12_MG1655_RecD  (588) AAIATRTERRSGLAALFSSRE-
  F_tularensis_LVS_RecD  (601) DIAQKDINRNSNISELVSLSTQ
              Consensus  (651)         R S IA L S
```
