# Supplementary material for: Development and Properties of Francisella tularensis Subsp. holarctica 15 NIIEG Vaccine Strain without the recD Gene
Source: Vaccines (Basel). 2022 Jan 11;10(1):108. doi: 10.3390/vaccines10010108 (PMC8778684; doi:10.3390/vaccines10010108)
Supplement: Supplementary file 1 [file vaccines-10-00108-s001.zip › Supplementary S2.pdf]

Supplementary 2. Comparison of the *recBCD* regions of *Francisella tularensis* subsp. *holarctica* LVS and *Francisella tularensis* subsp. *holarctica* 15 NIIEG

Francisella tularensis subsp. holarctica LVS, complete genome
Sequence ID: CP009694.1 Length: 1892177 Number of Matches: 1
Range 1: 1371162 to 1384489 GenBankGraphicsNext MatchPrevious Match

Query - Francisella tularensis subsp. holarctica 15 NIIEG

Alignment statistics for match #1

Score	Expect	Identities	Gaps
24613 bits(13328)	0.0	13328/13328(100%)	0/13328(0%)

```
Query  1        ATGCATATAAGTGAGCGCCGTAAATGGTATATAGGCTTTGCTGTTTTTGTGATGGTGATT  60
                ||||||||||||||||||||||||||||||||||||||||||||||||||||||||||||
Sbjct  1371162  ATGCATATAAGTGAGCGCCGTAAATGGTATATAGGCTTTGCTGTTTTTGTGATGGTGATT  1371221

Query  61       TTATTATCGCTTTCACTACTTCATGCTTCAATATGGTTTAGTATAGGGTTTGTCGCTGTT  120
                ||||||||||||||||||||||||||||||||||||||||||||||||||||||||||||
Sbjct  1371222  TTATTATCGCTTTCACTACTTCATGCTTCAATATGGTTTAGTATAGGGTTTGTCGCTGTT  1371281

Query  121      TTGGCAGTGATCGCAATCTATGATGTTAGCCAAACAAAGCATTCCATCCTAAGAAACTTT  180
                ||||||||||||||||||||||||||||||||||||||||||||||||||||||||||||
Sbjct  1371282  TTGGCAGTGATCGCAATCTATGATGTTAGCCAAACAAAGCATTCCATCCTAAGAAACTTT  1371341

Query  181      CCTATAGTAGGACATATGCGCTATATACTTGAGTTTTTACGTCCTGAAATTCAGCAGTAT  240
                ||||||||||||||||||||||||||||||||||||||||||||||||||||||||||||
Sbjct  1371342  CCTATAGTAGGACATATGCGCTATATACTTGAGTTTTTACGTCCTGAAATTCAGCAGTAT  1371401

Query  241      TTCATTGCTGATAATGAGAGTGAAAAGCCATTTGGTAGAGAGCTACGTTCGACAATCTAC  300
                ||||||||||||||||||||||||||||||||||||||||||||||||||||||||||||
Sbjct  1371402  TTCATTGCTGATAATGAGAGTGAAAAGCCATTTGGTAGAGAGCTACGTTCGACAATCTAC  1371461

Query  301      CGTCGTGCTAAGGGTATTAATGATACTGTTGCTTTTGGAACAGAGAAAGATATTTATAGG  360
                ||||||||||||||||||||||||||||||||||||||||||||||||||||||||||||
Sbjct  1371462  CGTCGTGCTAAGGGTATTAATGATACTGTTGCTTTTGGAACAGAGAAAGATATTTATAGG  1371521

Query  361      GTTGGTTATGAATGGGTAACACATTCACTCATGCCTAAGCATCTTGATGAAATTGAAACT  420
                ||||||||||||||||||||||||||||||||||||||||||||||||||||||||||||
Sbjct  1371522  GTTGGTTATGAATGGGTAACACATTCACTCATGCCTAAGCATCTTGATGAAATTGAAACT  1371581

Query  421      CGAGTTAAAATTGGTGGTTCTGATTGTAAACAACCATATATGGCATCGCATCTAAATATA  480
                ||||||||||||||||||||||||||||||||||||||||||||||||||||||||||||
Sbjct  1371582  CGAGTTAAAATTGGTGGTTCTGATTGTAAACAACCATATATGGCATCGCATCTAAATATA  1371641

Query  481      TCTGCGATGAGTTTTGGGGCATTATCTGCTAATGCCGTTATGGCTTTGAATAAGGGAGCT  540
                ||||||||||||||||||||||||||||||||||||||||||||||||||||||||||||
Sbjct  1371642  TCTGCGATGAGTTTTGGGGCATTATCTGCTAATGCCGTTATGGCTTTGAATAAGGGAGCT  1371701

Query  541      AAACTTGGAGGGTTTTATCAATGTACAGGTGAGGGCGGCTTGACTAAATATCATTTACAA  600
```

```
                 ||||||||||||||||||||||||||||||||||||||||||||||||||||||||||||
Sbjct  1371702   AAACTTGGAGGGTTTTATCAATGTACAGGTGAGGGCGGCTTGACTAAATATCATTTACAA  1371761

Query  601       GGTGGAGATCTTGTATTTCAAATTGGTACAGGCTATTTTGGCTGTCGTACTGATGATGGC  660
                 ||||||||||||||||||||||||||||||||||||||||||||||||||||||||||||
Sbjct  1371762   GGTGGAGATCTTGTATTTCAAATTGGTACAGGCTATTTTGGCTGTCGTACTGATGATGGC  1371821

Query  661       AAGTTCTCAGCAGAGAAATTTGTTGAAAAAGCAAATTTAGATAGAGTCAAAATGATAGAA  720
                 ||||||||||||||||||||||||||||||||||||||||||||||||||||||||||||
Sbjct  1371822   AAGTTCTCAGCAGAGAAATTTGTTGAAAAAGCAAATTTAGATAGAGTCAAAATGATAGAA  1371881

Query  721       ATTAAGTTATCTCAAGGGGCTAAACCATCTCATGGCGGGGTTTTGCCAGCAGCTAAGATA  780
                 ||||||||||||||||||||||||||||||||||||||||||||||||||||||||||||
Sbjct  1371882   ATTAAGTTATCTCAAGGGGCTAAACCATCTCATGGCGGGGTTTTGCCAGCAGCTAAGATA  1371941

Query  781       ACTCCAGAAATTGCTGAAATTAGGGGTGTTTCAATGGGCAAAGATGTACTATCACCTCCT  840
                 ||||||||||||||||||||||||||||||||||||||||||||||||||||||||||||
Sbjct  1371942   ACTCCAGAAATTGCTGAAATTAGGGGTGTTTCAATGGGCAAAGATGTACTATCACCTCCT  1372001

Query  841       GCGCATAGTGCTTTTTCGACACCGATAGAATTTTGCTATTTCATTAAACAGCTAAGAGAT  900
                 ||||||||||||||||||||||||||||||||||||||||||||||||||||||||||||
Sbjct  1372002   GCGCATAGTGCTTTTTCGACACCGATAGAATTTTGCTATTTCATTAAACAGCTAAGAGAT  1372061

Query  901       CTTTCTAATGGCAAACCAATAGGGTTTAAGCTATGTATTGGTAGTCATGTTGAGTTTTTA  960
                 ||||||||||||||||||||||||||||||||||||||||||||||||||||||||||||
Sbjct  1372062   CTTTCTAATGGCAAACCAATAGGGTTTAAGCTATGTATTGGTAGTCATGTTGAGTTTTTA  1372121

Query  961       GCTATTTGTAAAGCAATGCTTGAGACAGGAATAAGACCTGATTTTATAACTGTTGATGGC  1020
                 ||||||||||||||||||||||||||||||||||||||||||||||||||||||||||||
Sbjct  1372122   GCTATTTGTAAAGCAATGCTTGAGACAGGAATAAGACCTGATTTTATAACTGTTGATGGC  1372181

Query  1021      GCTGATGGTGGTACAGGGGCGGCACCACTTGAGTTCTCTAATCATATTGGTATGCCTTTA  1080
                 ||||||||||||||||||||||||||||||||||||||||||||||||||||||||||||
Sbjct  1372182   GCTGATGGTGGTACAGGGGCGGCACCACTTGAGTTCTCTAATCATATTGGTATGCCTTTA  1372241

Query  1081      GAAGATAGTTTAATCTTTGTGCATAATGCTTTGGTTGGTTGTGGCTTACGTGATGAGATT  1140
                 ||||||||||||||||||||||||||||||||||||||||||||||||||||||||||||
Sbjct  1372242   GAAGATAGTTTAATCTTTGTGCATAATGCTTTGGTTGGTTGTGGCTTACGTGATGAGATT  1372301

Query  1141      CGTATAATTGCAAGTAGTAAAGTAGCTACAGGTTTTGATATGGTGAGGTTATTTGCTATG  1200
                 ||||||||||||||||||||||||||||||||||||||||||||||||||||||||||||
Sbjct  1372302   CGTATAATTGCAAGTAGTAAAGTAGCTACAGGTTTTGATATGGTGAGGTTATTTGCTATG  1372361

Query  1201      GGAGCAGATACTTGTAACTCAGCTAGAGCAATGATGCTTGCTATCGGCTGTATTCAGTCA  1260
                 ||||||||||||||||||||||||||||||||||||||||||||||||||||||||||||
Sbjct  1372362   GGAGCAGATACTTGTAACTCAGCTAGAGCAATGATGCTTGCTATCGGCTGTATTCAGTCA  1372421

Query  1261      CGACAGTGTAATAACAACACTTGTCCAGTTGGTGTTGCTACACAAAATCCACGTCTTGAA  1320
```

```
                      ||||||||||||||||||||||||||||||||||||||||||||||||||||||||||||
Sbjct  1372422  CGACAGTGTAATAACAACACTTGTCCAGTTGGTGTTGCTACACAAAATCCACGTCTTGAA  1372481

Query  1321     AAAGCTTTGGTAGTTGAAGACAAAATGTATCGAGTTTATAATTTTCATAAAGTTACAATT  1380
                ||||||||||||||||||||||||||||||||||||||||||||||||||||||||||||
Sbjct  1372482  AAAGCTTTGGTAGTTGAAGACAAAATGTATCGAGTTTATAATTTTCATAAAGTTACAATT  1372541

Query  1381     CAATCTTGTTTAGAAATTATTGGAGCTATGGGGCTTATTTCTACTGATGATGTTGAGCCA  1440
                ||||||||||||||||||||||||||||||||||||||||||||||||||||||||||||
Sbjct  1372542  CAATCTTGTTTAGAAATTATTGGAGCTATGGGGCTTATTTCTACTGATGATGTTGAGCCA  1372601

Query  1441     GAAAACCTTAAAAAGCGTATATCTGTAAATGAGATTAAATCATATGCAGATCTGTATGAT  1500
                ||||||||||||||||||||||||||||||||||||||||||||||||||||||||||||
Sbjct  1372602  GAAAACCTTAAAAAGCGTATATCTGTAAATGAGATTAAATCATATGCAGATCTGTATGAT  1372661

Query  1501     TTTATTCCAGAGAGATGTTTAGTCGATGGTAATATCCCAGCTAGTTTTGCAAGATCTTGG  1560
                ||||||||||||||||||||||||||||||||||||||||||||||||||||||||||||
Sbjct  1372662  TTTATTCCAGAGAGATGTTTAGTCGATGGTAATATCCCAGCTAGTTTTGCAAGATCTTGG  1372721

Query  1561     GAAAAGGCTCGAGCTGATTCTTTTTAGTTTCTGAATATAAATAATAATTTTTGTCTAATC  1620
                ||||||||||||||||||||||||||||||||||||||||||||||||||||||||||||
Sbjct  1372722  GAAAAGGCTCGAGCTGATTCTTTTTAGTTTCTGAATATAAATAATAATTTTTGTCTAATC  1372781

Query  1621     AATTTGCTAAAATAGCTTCTATAAGTATTCTGATTAATCAAGCATGGCACTATACACTTA  1680
                ||||||||||||||||||||||||||||||||||||||||||||||||||||||||||||
Sbjct  1372782  AATTTGCTAAAATAGCTTCTATAAGTATTCTGATTAATCAAGCATGGCACTATACACTTA  1372841

Query  1681     CCCATCAAATAAACTTGAGTATCTTGTACAAGTTCTATCTAAACTTCTAGATGTTGaaaa  1740
                ||||||||||||||||||||||||||||||||||||||||||||||||||||||||||||
Sbjct  1372842  CCCATCAAATAAACTTGAGTATCTTGTACAAGTTCTATCTAAACTTCTAGATGTTGAAAA  1372901

Query  1741     aaaaGATTTATTTACACCAACACAGTTAATTGTTGGTAGCCGTGGTATGCAACATTGGTT  1800
                ||||||||||||||||||||||||||||||||||||||||||||||||||||||||||||
Sbjct  1372902  AAAAGATTTATTTACACCAACACAGTTAATTGTTGGTAGCCGTGGTATGCAACATTGGTT  1372961

Query  1801     AAGTATGCAACTAGCCGAATATCGCAATATTGCAATGAATCTCAAATATGACATGATAAA  1860
                ||||||||||||||||||||||||||||||||||||||||||||||||||||||||||||
Sbjct  1372962  AAGTATGCAACTAGCCGAATATCGCAATATTGCAATGAATCTCAAATATGACATGATAAA  1373021

Query  1861     TGGTTATATACTTGATATCTGCTATGAACTCACAGCAAAACAAGAATATAAAAAAGCATA  1920
                ||||||||||||||||||||||||||||||||||||||||||||||||||||||||||||
Sbjct  1373022  TGGTTATATACTTGATATCTGCTATGAACTCACAGCAAAACAAGAATATAAAAAAGCATA  1373081

Query  1921     TACAAAAGATATTTTAGCTTGGAGAGTTTTTAGGTTGCTATCTAGCCAAAATCAGGaaaa  1980
                ||||||||||||||||||||||||||||||||||||||||||||||||||||||||||||
Sbjct  1373082  TACAAAAGATATTTTAGCTTGGAGAGTTTTTAGGTTGCTATCTAGCCAAAATCAGGAAAA  1373141

Query  1981     aaaCACTCATTCCCACGCAGGTGGGAATCTCATAGCTGATGGATTAAGTTCAAAAGATAC  2040
```

```
                 ||||||||||||||||||||||||||||||||||||||||||||||||||||||||||||
Sbjct  1373142  AAACACTCATTCCCACGCAGGTGGGAATCTCATAGCTGATGGATTAAGTTCAAAAGATAC  1373201

Query  2041     TAGCCTACGCAGGTATGACAATAGAGATGGTTGGAGTAGTAAGCTTAAAGAGTACTACCA  2100
                ||||||||||||||||||||||||||||||||||||||||||||||||||||||||||||
Sbjct  1373202  TAGCCTACGCAGGTATGACAATAGAGATGGTTGGAGTAGTAAGCTTAAAGAGTACTACCA  1373261

Query  2101     AGATAGTGATCTCAAAAAATATCAGTTGTCAGTTAAAATAGCAGAGACCTTCTCAAAATA  2160
                ||||||||||||||||||||||||||||||||||||||||||||||||||||||||||||
Sbjct  1373262  AGATAGTGATCTCAAAAAATATCAGTTGTCAGTTAAAATAGCAGAGACCTTCTCAAAATA  1373321

Query  2161     TATATCATATCGTAGTGAGTGGCTACAAAAGTGGGAGAAGGACGAATATATAAATCCCTC  2220
                ||||||||||||||||||||||||||||||||||||||||||||||||||||||||||||
Sbjct  1373322  TATATCATATCGTAGTGAGTGGCTACAAAAGTGGGAGAAGGACGAATATATAAATCCCTC  1373381

Query  2221     TAAACTAGAGAATGATGAAGATTGGCAGATGCTAATTTGGCAGAATCTTGTCAAAGATAT  2280
                ||||||||||||||||||||||||||||||||||||||||||||||||||||||||||||
Sbjct  1373382  TAAACTAGAGAATGATGAAGATTGGCAGATGCTAATTTGGCAGAATCTTGTCAAAGATAT  1373441

Query  2281     TGCAGAAACACCATATAAAGTCCAATTAGAAGCACTTCAAAAGCTTGATAAACAAAACTT  2340
                ||||||||||||||||||||||||||||||||||||||||||||||||||||||||||||
Sbjct  1373442  TGCAGAAACACCATATAAAGTCCAATTAGAAGCACTTCAAAAGCTTGATAAACAAAACTT  1373501

Query  2341     AGAAAAAATTAAATATACCTAGTGATATTTATATATTTGGAGTTAACACAATATCTCCAAA  2400
                ||||||||||||||||||||||||||||||||||||||||||||||||||||||||||||
Sbjct  1373502  AGAAAAAATTAAATATACCTAGTGATATTTATATATTTGGAGTTAACACAATATCTCCAAA  1373561

Query  2401     AAATCTTAAATTTATATTTGAATTAGCTAAGTATATTAATGTACATATTCTATATATTAA  2460
                ||||||||||||||||||||||||||||||||||||||||||||||||||||||||||||
Sbjct  1373562  AAATCTTAAATTTATATTTGAATTAGCTAAGTATATTAATGTACATATTCTATATATTAA  1373621

Query  2461     TCCATGTAGTGAGTATTGGTATGATTTGCATAAAAGTAAAATATCAGCTTGGTTAGATAG  2520
                ||||||||||||||||||||||||||||||||||||||||||||||||||||||||||||
Sbjct  1373622  TCCATGTAGTGAGTATTGGTATGATTTGCATAAAAGTAAAATATCAGCTTGGTTAGATAG  1373681

Query  2521     TGATGACTATGAAATTCAACCACTTTTGGCAAATCTTGGACAGCAGGGTAAAGAATTTTT  2580
                ||||||||||||||||||||||||||||||||||||||||||||||||||||||||||||
Sbjct  1373682  TGATGACTATGAAATTCAACCACTTTTGGCAAATCTTGGACAGCAGGGTAAAGAATTTTT  1373741

Query  2581     TAATCAGCTTCTAGAAAATGAGCAACAGCAAGAATTAGAAGTATTCGAAAAGTTTGATAA  2640
                ||||||||||||||||||||||||||||||||||||||||||||||||||||||||||||
Sbjct  1373742  TAATCAGCTTCTAGAAAATGAGCAACAGCAAGAATTAGAAGTATTCGAAAAGTTTGATAA  1373801

Query  2641     AGAATCATTAGCTTTTGAGAAACTTACCGATAATAATCAAACACAGCTAGTAAGCCTACA  2700
                ||||||||||||||||||||||||||||||||||||||||||||||||||||||||||||
Sbjct  1373802  AGAATCATTAGCTTTTGAGAAACTTACCGATAATAATCAAACACAGCTAGTAAGCCTACA  1373861

Query  2701     GCGAAACTTACTTGAACTAGATTGCCAAAACCATGCCAAGCAAAAAGATTTAAGTATTAG  2760
```

```
                  ||||||||||||||||||||||||||||||||||||||||||||||||||||||||||||
Sbjct  1373862  GCGAAACTTACTTGAACTAGATTGCCAAAACCATGCCAAGCAAAAAGATTTAAGTATTAG  1373921

Query  2761     TATTAACTCTTGTCATAGTCCACTTAGAGAGGTGCAGATTTTGCATGATAAGCTTTTGGA  2820
                ||||||||||||||||||||||||||||||||||||||||||||||||||||||||||||
Sbjct  1373922  TATTAACTCTTGTCATAGTCCACTTAGAGAGGTGCAGATTTTGCATGATAAGCTTTTGGA  1373981

Query  2821     TATGATCAAAGCAGATCCAAATATCAAGCCTAGAGATATCCTAGTGATGTGCCCAAATAT  2880
                ||||||||||||||||||||||||||||||||||||||||||||||||||||||||||||
Sbjct  1373982  TATGATCAAAGCAGATCCAAATATCAAGCCTAGAGATATCCTAGTGATGTGCCCAAATAT  1374041

Query  2881     TGAAGATTATTCACCATATATTGATAGTGTTTTCTCAAGATATCCAACTGATAAAAAACT  2940
                ||||||||||||||||||||||||||||||||||||||||||||||||||||||||||||
Sbjct  1374042  TGAAGATTATTCACCATATATTGATAGTGTTTTCTCAAGATATCCAACTGATAAAAAACT  1374101

Query  2941     GCCATGCTCAATCGCAGATAGAACGCTACTTGATTCAGAGCCTTTAGCTGCTAGTTTTAT  3000
                ||||||||||||||||||||||||||||||||||||||||||||||||||||||||||||
Sbjct  1374102  GCCATGCTCAATCGCAGATAGAACGCTACTTGATTCAGAGCCTTTAGCTGCTAGTTTTAT  1374161

Query  3001     TGAGCTTTTGCAGTTGCCTGAGAGTAATTTTGAGGTTAATAAAATACTTGATTATCTAGC  3060
                ||||||||||||||||||||||||||||||||||||||||||||||||||||||||||||
Sbjct  1374162  TGAGCTTTTGCAGTTGCCTGAGAGTAATTTTGAGGTTAATAAAATACTTGATTATCTAGC  1374221

Query  3061     TGTACCGGCTATCCAACAGAAGTTTAAGATCACAGATGAACAATTAGAGGCTATTCGCTA  3120
                ||||||||||||||||||||||||||||||||||||||||||||||||||||||||||||
Sbjct  1374222  TGTACCGGCTATCCAACAGAAGTTTAAGATCACAGATGAACAATTAGAGGCTATTCGCTA  1374281

Query  3121     TTGGTTAAAAGAGTCATGTATTCATCATAGCAATAATGATCAGACATTCTCATGGAGCTG  3180
                ||||||||||||||||||||||||||||||||||||||||||||||||||||||||||||
Sbjct  1374282  TTGGTTAAAAGAGTCATGTATTCATCATAGCAATAATGATCAGACATTCTCATGGAGCTG  1374341

Query  3181     GGGACTAAGAAGATTAATGCTTGGGTTTAGCTATAGTGATAGTAGTTACATCATTGATGA  3240
                ||||||||||||||||||||||||||||||||||||||||||||||||||||||||||||
Sbjct  1374342  GGGACTAAGAAGATTAATGCTTGGGTTTAGCTATAGTGATAGTAGTTACATCATTGATGA  1374401

Query  3241     TAAATTAATGACGGTACCAGTTATTGAAGGAAGTGAGATCGCTGAACTTGGTGGTTTGTA  3300
                ||||||||||||||||||||||||||||||||||||||||||||||||||||||||||||
Sbjct  1374402  TAAATTAATGACGGTACCAGTTATTGAAGGAAGTGAGATCGCTGAACTTGGTGGTTTGTA  1374461

Query  3301     TGAGTTATTAGAGCTACTTGAAAGATATTCTCAAAATTTGCTTAAACCAAGAAGTCTTGC  3360
                ||||||||||||||||||||||||||||||||||||||||||||||||||||||||||||
Sbjct  1374462  TGAGTTATTAGAGCTACTTGAAAGATATTCTCAAAATTTGCTTAAACCAAGAAGTCTTGC  1374521

Query  3361     AAATTGGCAAGTTTATCTTTTAGAAATGTTTGATGATGTCTTTGATGTCACTAATGATGA  3420
                ||||||||||||||||||||||||||||||||||||||||||||||||||||||||||||
Sbjct  1374522  AAATTGGCAAGTTTATCTTTTAGAAATGTTTGATGATGTCTTTGATGTCACTAATGATGA  1374581

Query  3421     GCAATATATTGCTaaaaaaaTCAAAGATATAATCGCAAAAACAGTCACTACCGCTAAAAA  3480
```

```
                  ||||||||||||||||||||||||||||||||||||||||||||||||||||||||||||
Sbjct  1374582  GCAATATATTGCTAAAAAAATCAAAGATATAATCGCAAAAACAGTCACTACCGCTAAAAA  1374641

Query  3481     TATATTGTTAGATCAAGAAATTGATTTATATACAATTAGATATTGTTTAATTTCTCAATT  3540
                ||||||||||||||||||||||||||||||||||||||||||||||||||||||||||||
Sbjct  1374642  TATATTGTTAGATCAAGAAATTGATTTATATACAATTAGATATTGTTTAATTTCTCAATT  1374701

Query  3541     ATCAGAGCCAATTATTAATAACCATTTTTTAAATGGTAAGGTGACTTTTTGTTCGATGAC  3600
                ||||||||||||||||||||||||||||||||||||||||||||||||||||||||||||
Sbjct  1374702  ATCAGAGCCAATTATTAATAACCATTTTTTAAATGGTAAGGTGACTTTTTGTTCGATGAC  1374761

Query  3601     GCCGATGCGTAGTGTACCATTTAGAGTTATAGCTATGCTTGGATTAAATAATGGTAAGTT  3660
                ||||||||||||||||||||||||||||||||||||||||||||||||||||||||||||
Sbjct  1374762  GCCGATGCGTAGTGTACCATTTAGAGTTATAGCTATGCTTGGATTAAATAATGGTAAGTT  1374821

Query  3661     CCCACACCAAGAATCTGCAATTAGCTTTGATCTGATAGCAAGGCTTGGTAGaaaaaaaGG  3720
                ||||||||||||||||||||||||||||||||||||||||||||||||||||||||||||
Sbjct  1374822  CCCACACCAAGAATCTGCAATTAGCTTTGATCTGATAGCAAGGCTTGGTAGAAAAAAAGG  1374881

Query  3721     TGATAGAACCAAACGCGATGATGATAGATATTTGTTTTTAGAGGCTATTTTATCTGCTAG  3780
                ||||||||||||||||||||||||||||||||||||||||||||||||||||||||||||
Sbjct  1374882  TGATAGAACCAAACGCGATGATGATAGATATTTGTTTTTAGAGGCTATTTTATCTGCTAG  1374941

Query  3781     AGATTATTTGTATATAAGCTATATAGGTAGAAGTGTTAAGACTAATGTTGAGCAACAGCC  3840
                ||||||||||||||||||||||||||||||||||||||||||||||||||||||||||||
Sbjct  1374942  AGATTATTTGTATATAAGCTATATAGGTAGAAGTGTTAAGACTAATGTTGAGCAACAGCC  1375001

Query  3841     AAGTTTGATACTTAAAGAGTTGACTAGCTATTTGAATTTGAACTACGATTGGGGAAAGGA  3900
                ||||||||||||||||||||||||||||||||||||||||||||||||||||||||||||
Sbjct  1375002  AAGTTTGATACTTAAAGAGTTGACTAGCTATTTGAATTTGAACTACGATTGGGGAAAGGA  1375061

Query  3901     GGATATAAAAGAGTATCCATTGCATGCTTTTAGTTCTAAATGTTATTCTGACAGTTATAG  3960
                ||||||||||||||||||||||||||||||||||||||||||||||||||||||||||||
Sbjct  1375062  GGATATAAAAGAGTATCCATTGCATGCTTTTAGTTCTAAATGTTATTCTGACAGTTATAG  1375121

Query  3961     AAGCTATGATAAAGCTTGGTTGAAGTTGTTACAGTCTGAGCCAAGGGGCTTTTATGATTC  4020
                ||||||||||||||||||||||||||||||||||||||||||||||||||||||||||||
Sbjct  1375122  AAGCTATGATAAAGCTTGGTTGAAGTTGTTACAGTCTGAGCCAAGGGGCTTTTATGATTC  1375181

Query  4021     GGCTTCATTAAGTAACTGTGCTAATTTACCCAAAAATCTAAGTATTTCAAAACTTGTTAA  4080
                ||||||||||||||||||||||||||||||||||||||||||||||||||||||||||||
Sbjct  1375182  GGCTTCATTAAGTAACTGTGCTAATTTACCCAAAAATCTAAGTATTTCAAAACTTGTTAA  1375241

Query  4081     AGTTTTTGATGACCCTATTAAGGCTTATGCAAATTATACTTTAGAGCTGTATTTAGAAGA  4140
                ||||||||||||||||||||||||||||||||||||||||||||||||||||||||||||
Sbjct  1375242  AGTTTTTGATGACCCTATTAAGGCTTATGCAAATTATACTTTAGAGCTGTATTTAGAAGA  1375301

Query  4141     TGACTTTGAGGAGCTAGAAGATAGTGAACCATTTGATATCAATAGTTTGGATAAGCATAA  4200
```

```
                    ||||||||||||||||||||||||||||||||||||||||||||||||||||||||||||
Sbjct  1375302  TGACTTTGAGGAGCTAGAAGATAGTGAACCATTTGATATCAATAGTTTGGATAAGCATAA  1375361

Query  4201     GCTCAAACAAGCTTTGTTAAAAACTTTTGAAGATGAAAAAGACAAAGATTTAACAATAAA  4260
                ||||||||||||||||||||||||||||||||||||||||||||||||||||||||||||
Sbjct  1375362  GCTCAAACAAGCTTTGTTAAAAACTTTTGAAGATGAAAAAGACAAAGATTTAACAATAAA  1375421

Query  4261     AACAGCCAAACTAAGTGGCAAGCTTCCGGAGTCAGTACTTACTGATACTGAGATTAAAGA  4320
                ||||||||||||||||||||||||||||||||||||||||||||||||||||||||||||
Sbjct  1375422  AACAGCCAAACTAAGTGGCAAGCTTCCGGAGTCAGTACTTACTGATACTGAGATTAAAGA  1375481

Query  4321     GGAAGTTGAGAATATTCAAAAGCTACTAGACAAAATGAGCTTAGCAAGTTACGAGTCAAA  4380
                ||||||||||||||||||||||||||||||||||||||||||||||||||||||||||||
Sbjct  1375482  GGAAGTTGAGAATATTCAAAAGCTACTAGACAAAATGAGCTTAGCAAGTTACGAGTCAAA  1375541

Query  4381     ATATTTTCATCAAGAGATATTAGGGTATGAGTTAGAAGCTAATTGTTATATTAAAAATAA  4440
                ||||||||||||||||||||||||||||||||||||||||||||||||||||||||||||
Sbjct  1375542  ATATTTTCATCAAGAGATATTAGGGTATGAGTTAGAAGCTAATTGTTATATTAAAAATAA  1375601

Query  4441     TCAAATTATGTTATCTACACCATCTAGTTTGAATATAGGaaaaaaaTTTGAATTGTACTT  4500
                ||||||||||||||||||||||||||||||||||||||||||||||||||||||||||||
Sbjct  1375602  TCAAATTATGTTATCTACACCATCTAGTTTGAATATAGGAAAAAAATTTGAATTGTACTT  1375661

Query  4501     AATAGCTTTATTAGTAGCATATTCTGAACAAAGAGATATTAGCGCAGTCTATTATGGTAT  4560
                ||||||||||||||||||||||||||||||||||||||||||||||||||||||||||||
Sbjct  1375662  AATAGCTTTATTAGTAGCATATTCTGAACAAAGAGATATTAGCGCAGTCTATTATGGTAT  1375721

Query  4561     AGaaaaaaaTCAAGTAGAAGAATTTAGAGTAGAAAATATTGAATATACAACAGCTAAAGG  4620
                ||||||||||||||||||||||||||||||||||||||||||||||||||||||||||||
Sbjct  1375722  AGAAAAAAATCAAGTAGAAGAATTTAGAGTAGAAAATATTGAATATACAACAGCTAAAGG  1375781

Query  4621     AATATTAGAGCATTATATAAATCAAGCTGAGCAAATAGTAGTTAAGCCTCAACTAGCACA  4680
                ||||||||||||||||||||||||||||||||||||||||||||||||||||||||||||
Sbjct  1375782  AATATTAGAGCATTATATAAATCAAGCTGAGCAAATAGTAGTTAAGCCTCAACTAGCACA  1375841

Query  4681     TTTATCTTTAGCTGAAGCTATTTGTGATTATAAAAATAATACTGATAAGAAGAAGCAACA  4740
                ||||||||||||||||||||||||||||||||||||||||||||||||||||||||||||
Sbjct  1375842  TTTATCTTTAGCTGAAGCTATTTGTGATTATAAAAATAATACTGATAAGAAGAAGCAACA  1375901

Query  4741     GGCTTGGCAAAAAGTAATAAAATCATCACAACATAATTTCAAAGCTCTAGAAGATAATAG  4800
                ||||||||||||||||||||||||||||||||||||||||||||||||||||||||||||
Sbjct  1375902  GGCTTGGCAAAAAGTAATAAAATCATCACAACATAATTTCAAAGCTCTAGAAGATAATAG  1375961

Query  4801     TTATTTCAAGCTATTCTATAATAAGTTTCCAACTGTAGATGATTTTGAGGGTGAAAAAAT  4860
                ||||||||||||||||||||||||||||||||||||||||||||||||||||||||||||
Sbjct  1375962  TTATTTCAAGCTATTCTATAATAAGTTTCCAACTGTAGATGATTTTGAGGGTGAAAAAAT  1376021

Query  4861     TTACAAAAAGTTCTTTGAGGTTATTTATGAAAGTCAATCATGAGCATTCTAATATCCCAC  4920
```

```
                  ||||||||||||||||||||||||||||||||||||||||||||||||||||||||||||
Sbjct  1376022  TTACAAAAAGTTCTTTGAGGTTATTTATGAAAGTCAATCATGAGCATTCTAATATCCCAC  1376081

Query  4921     AGCTATACAAAAATATTTATATTAGCTTTGAGATTGCTCTAGCTGCTTGTATATGTTTTC  4980
                ||||||||||||||||||||||||||||||||||||||||||||||||||||||||||||
Sbjct  1376082  AGCTATACAAAAATATTTATATTAGCTTTGAGATTGCTCTAGCTGCTTGTATATGTTTTC  1376141

Query  4981     TGTTAGGGTTTTATATGTCGAATTTGTTACATCGTGGTCAGTCGATTATTGGAGGCTTTT  5040
                ||||||||||||||||||||||||||||||||||||||||||||||||||||||||||||
Sbjct  1376142  TGTTAGGGTTTTATATGTCGAATTTGTTACATCGTGGTCAGTCGATTATTGGAGGCTTTT  1376201

Query  5041     GGTGTTTGATTACAGTTTCAACAATCTTACAGCTAAGTATTAGAGATTCGTATTTGGCTG  5100
                ||||||||||||||||||||||||||||||||||||||||||||||||||||||||||||
Sbjct  1376202  GGTGTTTGATTACAGTTTCAACAATCTTACAGCTAAGTATTAGAGATTCGTATTTGGCTG  1376261

Query  5101     CTTTTCAAATATTGGTTGGCTCTGTGATAGGAGGGATTACGGCATTTATTTTTACATCAG  5160
                ||||||||||||||||||||||||||||||||||||||||||||||||||||||||||||
Sbjct  1376262  CTTTTCAAATATTGGTTGGCTCTGTGATAGGAGGGATTACGGCATTTATTTTTACATCAG  1376321

Query  5161     TTTTAGGCTATTACTACTATGTTATGATTTTGGCTGTAGCTATTAGTGTTTTTATTACTG  5220
                ||||||||||||||||||||||||||||||||||||||||||||||||||||||||||||
Sbjct  1376322  TTTTAGGCTATTACTACTATGTTATGATTTTGGCTGTAGCTATTAGTGTTTTTATTACTG  1376381

Query  5221     CAAGTATAGGGTTAGAAAATGCGGTTAAAATGAGTAGCGCAAATGCTGGAGTAATTGTCG  5280
                ||||||||||||||||||||||||||||||||||||||||||||||||||||||||||||
Sbjct  1376382  CAAGTATAGGGTTAGAAAATGCGGTTAAAATGAGTAGCGCAAATGCTGGAGTAATTGTCG  1376441

Query  5281     CATTAGGTCTGTATCAGCCTAGTTATTCACCTTTTCTAAATACAGGTTTACGCTTGATAG  5340
                ||||||||||||||||||||||||||||||||||||||||||||||||||||||||||||
Sbjct  1376442  CATTAGGTCTGTATCAGCCTAGTTATTCACCTTTTCTAAATACAGGTTTACGCTTGATAG  1376501

Query  5341     AGACTTTTAGTGGTACTGCAATAGCtttatttttttatatttattagtaggatttttaGGA  5400
                ||||||||||||||||||||||||||||||||||||||||||||||||||||||||||||
Sbjct  1376502  AGACTTTTAGTGGTACTGCAATAGCTTTATTTTTTATATTTATTAGTAGGATTTTTAGGA  1376561

Query  5401     TAAGAACAGACGACTAATATATAGATTTATAGGATTAAATGAGTAATAATGAAAAACCTA  5460
                ||||||||||||||||||||||||||||||||||||||||||||||||||||||||||||
Sbjct  1376562  TAAGAACAGACGACTAATATATAGATTTATAGGATTAAATGAGTAATAATGAAAAACCTA  1376621

Query  5461     AACGCAAAAAGAATAACCCTGCAAGGTCGCCATATTATTAAGGCAAGTGTTAAGCTCGAT  5520
                ||||||||||||||||||||||||||||||||||||||||||||||||||||||||||||
Sbjct  1376622  AACGCAAAAAGAATAACCCTGCAAGGTCGCCATATTATTAAGGCAAGTGTTAAGCTCGAT  1376681

Query  5521     GCAATACAGTATGACAGAATCTAAAAAGATGAGAAACATATGAACATACTACAGCGAATA  5580
                ||||||||||||||||||||||||||||||||||||||||||||||||||||||||||||
Sbjct  1376682  GCAATACAGTATGACAGAATCTAAAAAGATGAGAAACATATGAACATACTACAGCGAATA  1376741

Query  5581     CAAAATGGTGAAGATAGTTACACGCAATTTAAACAAGATATAAATAATGCTGATAGTCTT  5640
```

```
                  ||||||||||||||||||||||||||||||||||||||||||||||||||||||||||||
Sbjct  1376742  CAAAATGGTGAAGATAGTTACACGCAATTTAAACAAGATATAAATAATGCTGATAGTCTT  1376801

Query  5641     GCACAAGAGCTTGTTGCTTTTTCAAATGCTAATGGCGGTATTTTAATCATTGGTGTTGCT  5700
                ||||||||||||||||||||||||||||||||||||||||||||||||||||||||||||
Sbjct  1376802  GCACAAGAGCTTGTTGCTTTTTCAAATGCTAATGGCGGTATTTTAATCATTGGTGTTGCT  1376861

Query  5701     GATGATGGTAAAATTATCGGATTATCAAAAGATGATATTCATAGGATAAATCAGCTTATA  5760
                ||||||||||||||||||||||||||||||||||||||||||||||||||||||||||||
Sbjct  1376862  GATGATGGTAAAATTATCGGATTATCAAAAGATGATATTCATAGGATAAATCAGCTTATA  1376921

Query  5761     AGTAATGTTTGCAGTACAAATATAAAACCACCAATAAGCGTACTAACAAAGATAGAGAAT  5820
                ||||||||||||||||||||||||||||||||||||||||||||||||||||||||||||
Sbjct  1376922  AGTAATGTTTGCAGTACAAATATAAAACCACCAATAAGCGTACTAACAAAGATAGAGAAT  1376981

Query  5821     ATAGATGATAAAGAAATACTTATAATAGAAATAAACAATGGAGTAAATAAACCATATAGT  5880
                ||||||||||||||||||||||||||||||||||||||||||||||||||||||||||||
Sbjct  1376982  ATAGATGATAAAGAAATACTTATAATAGAAATAAACAATGGAGTAAATAAACCATATAGT  1377041

Query  5881     ACTAACAAAGGAGTATATTTGACAAAAGTTGGTAGTGATAAAAGAAAAATGTCTCAAGAA  5940
                ||||||||||||||||||||||||||||||||||||||||||||||||||||||||||||
Sbjct  1377042  ACTAACAAAGGAGTATATTTGACAAAAGTTGGTAGTGATAAAAGAAAAATGTCTCAAGAA  1377101

Query  5941     GAGCTAGGTAGACTTTTTAGACAGGCTCCAAAACTGTATCCTGATGAAGAAGTGCTATAT  6000
                ||||||||||||||||||||||||||||||||||||||||||||||||||||||||||||
Sbjct  1377102  GAGCTAGGTAGACTTTTTAGACAGGCTCCAAAACTGTATCCTGATGAAGAAGTGCTATAT  1377161

Query  6001     AAAACATCGCTAGAAAACTTTGATGTTTCAAAGTTTAGAAAGTTTCTAAAAACAGATAAC  6060
                ||||||||||||||||||||||||||||||||||||||||||||||||||||||||||||
Sbjct  1377162  AAAACATCGCTAGAAAACTTTGATGTTTCAAAGTTTAGAAAGTTTCTAAAAACAGATAAC  1377221

Query  6061     TATACTACATTTTCAAAACTAGAGAATGGTAGTCTAGATTTATCAACTATTCTAGAAAAT  6120
                ||||||||||||||||||||||||||||||||||||||||||||||||||||||||||||
Sbjct  1377222  TATACTACATTTTCAAAACTAGAGAATGGTAGTCTAGATTTATCAACTATTCTAGAAAAT  1377281

Query  6121     AAAGATTTAGCAAAAGATGGCAAACTAACTTTAGCCGGTAATCTTATATTTGGAGAAGAG  6180
                ||||||||||||||||||||||||||||||||||||||||||||||||||||||||||||
Sbjct  1377282  AAAGATTTAGCAAAAGATGGCAAACTAACTTTAGCCGGTAATCTTATATTTGGAGAAGAG  1377341

Query  6181     CCACAGCGGTTTTGTCCAAGCTTTTATATAGACTGCTGTTATTTTGATGGTAATGATATT  6240
                ||||||||||||||||||||||||||||||||||||||||||||||||||||||||||||
Sbjct  1377342  CCACAGCGGTTTTGTCCAAGCTTTTATATAGACTGCTGTTATTTTGATGGTAATGATATT  1377401

Query  6241     TCTGTATCGAAATTTATATCTAAAAAAGTTATCAAAGGAACATTTTCTGAACTATATGAA  6300
                ||||||||||||||||||||||||||||||||||||||||||||||||||||||||||||
Sbjct  1377402  TCTGTATCGAAATTTATATCTAAAAAAGTTATCAAAGGAACATTTTCTGAACTATATGAA  1377461

Query  6301     GATACCATGAGATTTTTGCTTAGTCAGTTAAAAAGTTATCAAATAGATTTAGATTTTAAC  6360
```

```
                  ||||||||||||||||||||||||||||||||||||||||||||||||||||||||||||
Sbjct  1377462  GATACCATGAGATTTTTGCTTAGTCAGTTAAAAAGTTATCAAATAGATTTAGATTTTAAC  1377521

Query  6361     TCAAACCCACAATTAGAGGTAAATGAATCTGCACTAACAGAGATAGTTGTAAATGCACTT  6420
                ||||||||||||||||||||||||||||||||||||||||||||||||||||||||||||
Sbjct  1377522  TCAAACCCACAATTAGAGGTAAATGAATCTGCACTAACAGAGATAGTTGTAAATGCACTT  1377581

Query  6421     GTGCATAGAGACTATTATATAAACTCTTCTATAAAGGTTTTTATATTTCATAACCGTGTT  6480
                ||||||||||||||||||||||||||||||||||||||||||||||||||||||||||||
Sbjct  1377582  GTGCATAGAGACTATTATATAAACTCTTCTATAAAGGTTTTTATATTTCATAACCGTGTT  1377641

Query  6481     GAGATTATTAGCCCTGGTAAATTACCTGACACTTTAACAGTAGATAAGATTAAAAATGGC  6540
                ||||||||||||||||||||||||||||||||||||||||||||||||||||||||||||
Sbjct  1377642  GAGATTATTAGCCCTGGTAAATTACCTGACACTTTAACAGTAGATAAGATTAAAAATGGC  1377701

Query  6541     ATAGCAATTCAGCGAAACCCTATCTTAGCCTCAATATGCAAAACATTATTACCTTATACA  6600
                ||||||||||||||||||||||||||||||||||||||||||||||||||||||||||||
Sbjct  1377702  ATAGCAATTCAGCGAAACCCTATCTTAGCCTCAATATGCAAAACATTATTACCTTATACA  1377761

Query  6601     GGTTATGGTAGTGGAATCAAAAGAGCTATTGAGCTAATTCCTAATATTGAATTTATAAAT  6660
                ||||||||||||||||||||||||||||||||||||||||||||||||||||||||||||
Sbjct  1377762  GGTTATGGTAGTGGAATCAAAAGAGCTATTGAGCTAATTCCTAATATTGAATTTATAAAT  1377821

Query  6661     GATATAGATAGTGAACAGTTTAGATGTATTATaaaaagagatgaaagttcaccaaaagag  6720
                ||||||||||||||||||||||||||||||||||||||||||||||||||||||||||||
Sbjct  1377822  GATATAGATAGTGAACAGTTTAGATGTATTATAAAAAGAGATGAAAGTTCACCAAAAGAG  1377881

Query  6721     caagaaagttcaccaaaagagcaagaaagttcaccaaaagagcaagaaagttcaccaaTA  6780
                ||||||||||||||||||||||||||||||||||||||||||||||||||||||||||||
Sbjct  1377882  CAAGAAAGTTCACCAAAAGAGCAAGAAAGTTCACCAAAAGAGCAAGAAAGTTCACCAATA  1377941

Query  6781     AATATAAGTTATGGTGTTGGCAGTTCACCAAAAACAGATAAGCAGATTTTAGAAATGTTT  6840
                ||||||||||||||||||||||||||||||||||||||||||||||||||||||||||||
Sbjct  1377942  AATATAAGTTATGGTGTTGGCAGTTCACCAAAAACAGATAAGCAGATTTTAGAAATGTTT  1378001

Query  6841     AGAGAAAACCCAAAAATCACAATCAAGGAAATAGCAGAGAGATTGTCTATCACAGATAGA  6900
                ||||||||||||||||||||||||||||||||||||||||||||||||||||||||||||
Sbjct  1378002  AGAGAAAACCCAAAAATCACAATCAAGGAAATAGCAGAGAGATTGTCTATCACAGATAGA  1378061

Query  6901     GCTGTCAAAAAGCATATAGAAAAACTAAAATACCAAGGATTAATTGCTAGAGAAGGAAGC  6960
                ||||||||||||||||||||||||||||||||||||||||||||||||||||||||||||
Sbjct  1378062  GCTGTCAAAAAGCATATAGAAAAACTAAAATACCAAGGATTAATTGCTAGAGAAGGAAGC  1378121

Query  6961     CCTCGTAACGGCTATTGGAGAGTTATATAAATTGCAATGATGAAAGTTTTAAATAGACTT  7020
                ||||||||||||||||||||||||||||||||||||||||||||||||||||||||||||
Sbjct  1378122  CCTCGTAACGGCTATTGGAGAGTTATATAAATTGCAATGATGAAAGTTTTAAATAGACTT  1378181

Query  7021     TGAACTATCTAGATTTTATGGGTATATAGAAGACACAGATTATGGACAACCTAAACGCAA  7080
```

```
                ||||||||||||||||||||||||||||||||||||||||||||||||||||||||||||
Sbjct  1378182  TGAACTATCTAGATTTTATGGGTATATAGAAGACACAGATTATGGACAACCTAAACGCAA  1378241

Query  7081     ACATAATAACTCTACAAGGTCGCCATATTATCGAGGCAAGTGCTGGTACTGGTAAGACTT  7140
                ||||||||||||||||||||||||||||||||||||||||||||||||||||||||||||
Sbjct  1378242  ACATAATAACTCTACAAGGTCGCCATATTATCGAGGCAAGTGCTGGTACTGGTAAGACTT  1378301

Query  7141     TTAATATTACTAAGTTGTATATCCGACTTTTGCTAGaaaaaaaaTTATTGCCAAGTAATA  7200
                ||||||||||||||||||||||||||||||||||||||||||||||||||||||||||||
Sbjct  1378302  TTAATATTACTAAGTTGTATATCCGACTTTTGCTAGAAAAAAAATTATTGCCAAGTAATA  1378361

Query  7201     TCCTTGTAATGACCTTTACCAAAGATGCAACCCAAGAGATTATTGGTAGAGTTGAAAAAG  7260
                ||||||||||||||||||||||||||||||||||||||||||||||||||||||||||||
Sbjct  1378362  TCCTTGTAATGACCTTTACCAAAGATGCAACCCAAGAGATTATTGGTAGAGTTGAAAAAG  1378421

Query  7261     AGATTCGAGATGTATTAGCAAGTTATACAGATGAGAAAAAGGAGTCTGACAAAGAAAACT  7320
                ||||||||||||||||||||||||||||||||||||||||||||||||||||||||||||
Sbjct  1378422  AGATTCGAGATGTATTAGCAAGTTATACAGATGAGAAAAAGGAGTCTGACAAAGAAAACT  1378481

Query  7321     ACAAACATCTAAAAAGATCATTACTTGAGATAGATGAGGCAGCTATATTTACTATCCATG  7380
                ||||||||||||||||||||||||||||||||||||||||||||||||||||||||||||
Sbjct  1378482  ACAAACATCTAAAAAGATCATTACTTGAGATAGATGAGGCAGCTATATTTACTATCCATG  1378541

Query  7381     GTTTTTGTAAAAAAGTCTTAAGTGAGCAAGCCTTTGCTAGCGGTATCGAGATGGATGTCT  7440
                ||||||||||||||||||||||||||||||||||||||||||||||||||||||||||||
Sbjct  1378542  GTTTTTGTAAAAAAGTCTTAAGTGAGCAAGCCTTTGCTAGCGGTATCGAGATGGATGTCT  1378601

Query  7441     CTATGGAGGTTGATACTTCAGATATTTTTCAAAAGGTTGTAGAGGATTTTTTCAGAAAAC  7500
                ||||||||||||||||||||||||||||||||||||||||||||||||||||||||||||
Sbjct  1378602  CTATGGAGGTTGATACTTCAGATATTTTTCAAAAGGTTGTAGAGGATTTTTTCAGAAAAC  1378661

Query  7501     ATATCAATAAAAGCGAAACTAATTTTGGATATTTACAAATATATAAATTACATACTCCAG  7560
                ||||||||||||||||||||||||||||||||||||||||||||||||||||||||||||
Sbjct  1378662  ATATCAATAAAAGCGAAACTAATTTTGGATATTTACAAATATATAAATTACATACTCCAG  1378721

Query  7561     AGAAATTTTTAGATGACCTTGAGAATATTATTAGGTCTAATTATGAGATTTTAACAAAGC  7620
                ||||||||||||||||||||||||||||||||||||||||||||||||||||||||||||
Sbjct  1378722  AGAAATTTTTAGATGACCTTGAGAATATTATTAGGTCTAATTATGAGATTTTAACAAAGC  1378781

Query  7621     AAGCTATAAGCTTAGACGAATTTAAGATACTCAAAAAACAACAGTTAGAATTATTTATTA  7680
                ||||||||||||||||||||||||||||||||||||||||||||||||||||||||||||
Sbjct  1378782  AAGCTATAAGCTTAGACGAATTTAAGATACTCAAAAAACAACAGTTAGAATTATTTATTA  1378841

Query  7681     ATAATCACGATATCGTAGATGATTTCTTATATAAATTAGGAAAAAGTGAGCCACAGGGCA  7740
                ||||||||||||||||||||||||||||||||||||||||||||||||||||||||||||
Sbjct  1378842  ATAATCACGATATCGTAGATGATTTCTTATATAAATTAGGAAAAAGTGAGCCACAGGGCA  1378901

Query  7741     AGAGAGTAGATGAGTATCATAGGGTTTTAGAATGGTTGAAATTAGATAATCAAACACTAT  7800
```

```
                  ||||||||||||||||||||||||||||||||||||||||||||||||||||||||||||
Sbjct  1378902  AGAGAGTAGATGAGTATCATAGGGTTTTAGAATGGTTGAAATTAGATAATCAAACACTAT  1378961

Query  7801     TCCCAGAGAATATTTCTATAATTACAGATGGCAGAAAGATAAGTGCAAAACTAATAAAAC  7860
                ||||||||||||||||||||||||||||||||||||||||||||||||||||||||||||
Sbjct  1378962  TCCCAGAGAATATTTCTATAATTACAGATGGCAGAAAGATAAGTGCAAAACTAATAAAAC  1379021

Query  7861     CAATTTTTATTGGCGTAAAAGAATTAAGAGACCTGCAACAAGAAATAAAACAAGCACAAG  7920
                ||||||||||||||||||||||||||||||||||||||||||||||||||||||||||||
Sbjct  1379022  CAATTTTTATTGGCGTAAAAGAATTAAGAGACCTGCAACAAGAAATAAAACAAGCACAAG  1379081

Query  7921     CAGCACAATTTATCAGAAAAGCATGCTTGCAAATCCGCCAAGATTTTGCAAAGGCAAAAG  7980
                ||||||||||||||||||||||||||||||||||||||||||||||||||||||||||||
Sbjct  1379082  CAGCACAATTTATCAGAAAAGCATGCTTGCAAATCCGCCAAGATTTTGCAAAGGCAAAAG  1379141

Query  7981     ATCAAAAGGGTGTTTTAGACTTTGATGATTTGATTACCAAGCTTTGCCAAAGTGTCAAAA  8040
                ||||||||||||||||||||||||||||||||||||||||||||||||||||||||||||
Sbjct  1379142  ATCAAAAGGGTGTTTTAGACTTTGATGATTTGATTACCAAGCTTTGCCAAAGTGTCAAAA  1379201

Query  8041     AATCGCCAGAGTTAGTCAAAACTTTGCAAAAACAATATCCAGTTGCTCTTATAGATGAGT  8100
                ||||||||||||||||||||||||||||||||||||||||||||||||||||||||||||
Sbjct  1379202  AATCGCCAGAGTTAGTCAAAACTTTGCAAAAACAATATCCAGTTGCTCTTATAGATGAGT  1379261

Query  8101     TTCAAGATACTGATGCTGAGCAGTATGAGATATTAGATACTATTTATCCTTTGAAGAATA  8160
                ||||||||||||||||||||||||||||||||||||||||||||||||||||||||||||
Sbjct  1379262  TTCAAGATACTGATGCTGAGCAGTATGAGATATTAGATACTATTTATCCTTTGAAGAATA  1379321

Query  8161     GTAGTAGTCATTCCCACAAAGGTAAGAATCTCCTTACCAATGGATTAAATTTAAAAGATA  8220
                ||||||||||||||||||||||||||||||||||||||||||||||||||||||||||||
Sbjct  1379322  GTAGTAGTCATTCCCACAAAGGTAAGAATCTCCTTACCAATGGATTAAATTTAAAAGATA  1379381

Query  8221     CCCGCTTACGCGAGTATGACAGTAGTTCTGATCAGAACGATAGCAATCTTCTGCTCTTAA  8280
                ||||||||||||||||||||||||||||||||||||||||||||||||||||||||||||
Sbjct  1379382  CCCGCTTACGCGAGTATGACAGTAGTTCTGATCAGAACGATAGCAATCTTCTGCTCTTAA  1379441

Query  8281     TGATAGGCGATCCAAAACAGGCAATTTATGGCTTTAGAGGTGGAGATATCTTCACATATC  8340
                ||||||||||||||||||||||||||||||||||||||||||||||||||||||||||||
Sbjct  1379442  TGATAGGCGATCCAAAACAGGCAATTTATGGCTTTAGAGGTGGAGATATCTTCACATATC  1379501

Query  8341     TAAAAGCTAAAGATAGTTGTCCAGAAGAAAATCAATGGAGTATGGACACCAACTGGCGTT  8400
                ||||||||||||||||||||||||||||||||||||||||||||||||||||||||||||
Sbjct  1379502  TAAAAGCTAAAGATAGTTGTCCAGAAGAAAATCAATGGAGTATGGACACCAACTGGCGTT  1379561

Query  8401     CAACTGGAGAGATGATAAAAGCTTATAATAGACTTTTTTATAAACAAGATTATCAGTCAG  8460
                ||||||||||||||||||||||||||||||||||||||||||||||||||||||||||||
Sbjct  1379562  CAACTGGAGAGATGATAAAAGCTTATAATAGACTTTTTTATAAACAAGATTATCAGTCAG  1379621

Query  8461     AAGAAGAGGGGCAAATTGGAACTAATATTTTTAGTGATGGTATTGGATATCAGCTTGTCA  8520
```

```
                 ||||||||||||||||||||||||||||||||||||||||||||||||||||||||||||
Sbjct  1379622   AAGAAGAGGGGCAAATTGGAACTAATATTTTTAGTGATGGTATTGGATATCAGCTTGTCA  1379681

Query  8521      AAGCTTCACCGGATGCTGATaaaaaaaCAAAAGATTTTGACGATAATTTTAAACCTATTA  8580
                 ||||||||||||||||||||||||||||||||||||||||||||||||||||||||||||
Sbjct  1379682   AAGCTTCACCGGATGCTGATAAAAAAACAAAAGATTTTGACGATAATTTTAAACCTATTA  1379741

Query  8581      ATTATTTCTATTATGAAGTAGCCGAAGATGATAATAAAAGTGATATTGATACGAATTTAG  8640
                 ||||||||||||||||||||||||||||||||||||||||||||||||||||||||||||
Sbjct  1379742   ATTATTTCTATTATGAAGTAGCCGAAGATGATAATAAAAGTGATATTGATACGAATTTAG  1379801

Query  8641      CATTATGGACAGTAAATGAAATTACTAGATTACTAAATACACAAAAAGTAGCAGAAAATG  8700
                 ||||||||||||||||||||||||||||||||||||||||||||||||||||||||||||
Sbjct  1379802   CATTATGGACAGTAAATGAAATTACTAGATTACTAAATACACAAAAAGTAGCAGAAAATG  1379861

Query  8701      ATATAGCTATTTTGGTAGAAAATGGCAAACAGGCAAAGATTATCCAACAAGCTTTGCAAG  8760
                 ||||||||||||||||||||||||||||||||||||||||||||||||||||||||||||
Sbjct  1379862   ATATAGCTATTTTGGTAGAAAATGGCAAACAGGCAAAGATTATCCAACAAGCTTTGCAAG  1379921

Query  8761      CTAAAAATCTAAGCTCAGTATACCTAAGCCAGCGCGATAATGTCTATCACAGTCAAGAGG  8820
                 ||||||||||||||||||||||||||||||||||||||||||||||||||||||||||||
Sbjct  1379922   CTAAAAATCTAAGCTCAGTATACCTAAGCCAGCGCGATAATGTCTATCACAGTCAAGAGG  1379981

Query  8821      CTAAAGAGATCTTAGCTCTTATGGAGGGTATAAATGATCTTGAAAATAAATCTATGTTGA  8880
                 ||||||||||||||||||||||||||||||||||||||||||||||||||||||||||||
Sbjct  1379982   CTAAAGAGATCTTAGCTCTTATGGAGGGTATAAATGATCTTGAAAATAAATCTATGTTGA  1380041

Query  8881      AAAGGGCATTATCGACTAGTTTACTCGGTGGTAGAGCTGATAAGTTTATTAGCTATATTG  8940
                 ||||||||||||||||||||||||||||||||||||||||||||||||||||||||||||
Sbjct  1380042   AAAGGGCATTATCGACTAGTTTACTCGGTGGTAGAGCTGATAAGTTTATTAGCTATATTG  1380101

Query  8941      ATGAAAATGATGTCTCAGCGTGGGATGATGAGATAGAAAAAGCCAAATCTCTAAGGCAAC  9000
                 ||||||||||||||||||||||||||||||||||||||||||||||||||||||||||||
Sbjct  1380102   ATGAAAATGATGTCTCAGCGTGGGATGATGAGATAGAAAAAGCCAAATCTCTAAGGCAAC  1380161

Query  9001      AATGGCATAAATATGGATTTATGGCATTTATTATGCAGATTATACATGAGAATTTCACTC  9060
                 ||||||||||||||||||||||||||||||||||||||||||||||||||||||||||||
Sbjct  1380162   AATGGCATAAATATGGATTTATGGCATTTATTATGCAGATTATACATGAGAATTTCACTC  1380221

Query  9061      AAAAAGCAGATTCAAAAGAGCGTATTATTACAAATATTCTTCATTTAGCAGAACTTATTA  9120
                 ||||||||||||||||||||||||||||||||||||||||||||||||||||||||||||
Sbjct  1380222   AAAAAGCAGATTCAAAAGAGCGTATTATTACAAATATTCTTCATTTAGCAGAACTTATTA  1380281

Query  9121      AAGTGGCTGAGAATAAATATAAACACCCTAATCAACTCATTAAATGGTATCGCCACCAAC  9180
                 ||||||||||||||||||||||||||||||||||||||||||||||||||||||||||||
Sbjct  1380282   AAGTGGCTGAGAATAAATATAAACACCCTAATCAACTCATTAAATGGTATCGCCACCAAC  1380341

Query  9181      TCAACAATACAGCTACAAGTGAGGGTGAACTTAGATTAGAAAGTGATGACAACTTGATTA  9240
```

```
                 ||||||||||||||||||||||||||||||||||||||||||||||||||||||||||||
Sbjct  1380342  TCAACAATACAGCTACAAGTGAGGGTGAACTTAGATTAGAAAGTGATGACAACTTGATTA  1380401

Query  9241     AGATAATTACAATCCATGGATCAAAAGGACTTGAGTATTCAGTTGTGTTTATCCCGTTTG  9300
                ||||||||||||||||||||||||||||||||||||||||||||||||||||||||||||
Sbjct  1380402  AGATAATTACAATCCATGGATCAAAAGGACTTGAGTATTCAGTTGTGTTTATCCCGTTTG  1380461

Query  9301     CTTGCTATGCAAGCACAAAGAAGTTTGAGACAAGTAATTTTACTAAATATTATGATGGTG  9360
                ||||||||||||||||||||||||||||||||||||||||||||||||||||||||||||
Sbjct  1380462  CTTGCTATGCAAGCACAAAGAAGTTTGAGACAAGTAATTTTACTAAATATTATGATGGTG  1380521

Query  9361     ACCTAAAACAAACTGTCTACAAAATTGGCAAAGATGATAGTGTGAAACAGCAAGCTGATA  9420
                ||||||||||||||||||||||||||||||||||||||||||||||||||||||||||||
Sbjct  1380522  ACCTAAAACAAACTGTCTACAAAATTGGCAAAGATGATAGTGTGAAACAGCAAGCTGATA  1380581

Query  9421     AAGAGGTTATAGAAGAATTAATGCGTCTATTTTATGTAGCTGTTACGCGTGCAGAGCATA  9480
                ||||||||||||||||||||||||||||||||||||||||||||||||||||||||||||
Sbjct  1380582  AAGAGGTTATAGAAGAATTAATGCGTCTATTTTATGTAGCTGTTACGCGTGCAGAGCATA  1380641

Query  9481     GATGTTATATTGGGGTTGCAAAATATAATAATAGCGAAAAGTCACCATTAGCAAGATTCT  9540
                ||||||||||||||||||||||||||||||||||||||||||||||||||||||||||||
Sbjct  1380642  GATGTTATATTGGGGTTGCAAAATATAATAATAGCGAAAAGTCACCATTAGCAAGATTCT  1380701

Query  9541     TAGGTTATCAAAAAGATGATGATTGGTTAGAGAAGATCCAAAGCATAACAGCAAATCCTG  9600
                ||||||||||||||||||||||||||||||||||||||||||||||||||||||||||||
Sbjct  1380702  TAGGTTATCAAAAAGATGATGATTGGTTAGAGAAGATCCAAAGCATAACAGCAAATCCTG  1380761

Query  9601     CTAATCAAAGTCTACTGATAAATATTGCAGATATGCGGGAATTTAGTTTAAAAAATTTAT  9660
                ||||||||||||||||||||||||||||||||||||||||||||||||||||||||||||
Sbjct  1380762  CTAATCAAAGTCTACTGATAAATATTGCAGATATGCGGGAATTTAGTTTAAAAAATTTAT  1380821

Query  9661     CTAGCAAAGATAAAAATTCTGATTATGATACTCTCAAAGCAAATTATATTAGTAAACTAG  9720
                ||||||||||||||||||||||||||||||||||||||||||||||||||||||||||||
Sbjct  1380822  CTAGCAAAGATAAAAATTCTGATTATGATACTCTCAAAGCAAATTATATTAGTAAACTAG  1380881

Query  9721     AAAATGATAGTTGGGAGATGTTATCTTTCTCAAAAATATCAAAGTCAAAAGTTCAAAATA  9780
                ||||||||||||||||||||||||||||||||||||||||||||||||||||||||||||
Sbjct  1380882  AAAATGATAGTTGGGAGATGTTATCTTTCTCAAAAATATCAAAGTCAAAAGTTCAAAATA  1380941

Query  9781     CTGCATTAGAAAAAGAAATTGACGAGGCAGAAGATGATAAAACTCAAGCTTCAGACAGAA  9840
                ||||||||||||||||||||||||||||||||||||||||||||||||||||||||||||
Sbjct  1380942  CTGCATTAGAAAAAGAAATTGACGAGGCAGAAGATGATAAAACTCAAGCTTCAGACAGAA  1381001

Query  9841     AGCTAGCATTTAGATTTACAGCTTCAAAAGGAGCTGATATGGGTAATATTCTGCATAATG  9900
                ||||||||||||||||||||||||||||||||||||||||||||||||||||||||||||
Sbjct  1381002  AGCTAGCATTTAGATTTACAGCTTCAAAAGGAGCTGATATGGGTAATATTCTGCATAATG  1381061

Query  9901     TTTTAGAGCATACAGATTTTAGTTTAGGTGAGATAGATGGTAATCTTTTACAAGAACAAA  9960
```

```
                    ||||||||||||||||||||||||||||||||||||||||||||||||||||||||||||
Sbjct  1381062  TTTTAGAGCATACAGATTTTAGTTTAGGTGAGATAGATGGTAATCTTTTACAAGAACAAA  1381121

Query  9961     TGGATAGATATAAAGTAGTTGCTGCAGAAGACTTCGATAATCTTAAATTATGGTTGGAAG  10020
                ||||||||||||||||||||||||||||||||||||||||||||||||||||||||||||
Sbjct  1381122  TGGATAGATATAAAGTAGTTGCTGCAGAAGACTTCGATAATCTTAAATTATGGTTGGAAG  1381181

Query  10021    AATGTTTAGAAGCACATATACCTTATATAGATACCAGTTTTTCTAGCTTAAATGAGAGTA  10080
                ||||||||||||||||||||||||||||||||||||||||||||||||||||||||||||
Sbjct  1381182  AATGTTTAGAAGCACATATACCTTATATAGATACCAGTTTTTCTAGCTTAAATGAGAGTA  1381241

Query  10081    TTCAATATGGTTTATTTGATATCAAGGATAAAGGCTTTTGCTTAAAAACTATCCCAAATT  10140
                ||||||||||||||||||||||||||||||||||||||||||||||||||||||||||||
Sbjct  1381242  TTCAATATGGTTTATTTGATATCAAGGATAAAGGCTTTTGCTTAAAAACTATCCCAAATT  1381301

Query  10141    CGAAAACTCTAAAAGAGGCGGAGTTTTATTTTCCTGTTAAAAATGAGAATTTGTATAAAA  10200
                ||||||||||||||||||||||||||||||||||||||||||||||||||||||||||||
Sbjct  1381302  CGAAAACTCTAAAAGAGGCGGAGTTTTATTTTCCTGTTAAAAATGAGAATTTGTATAAAA  1381361

Query  10201    CAAATATATTAGAAATCTTGCAAGAGTATAGAGAGCTTACAAAACTGAAGACATATAGTC  10260
                ||||||||||||||||||||||||||||||||||||||||||||||||||||||||||||
Sbjct  1381362  CAAATATATTAGAAATCTTGCAAGAGTATAGAGAGCTTACAAAACTGAAGACATATAGTC  1381421

Query  10261    ATTCCTACGTAGGTGGGAATCTTCCAACTAACTACCAAGATCTAAGAGATACCCGCCTCC  10320
                ||||||||||||||||||||||||||||||||||||||||||||||||||||||||||||
Sbjct  1381422  ATTCCTACGTAGGTGGGAATCTTCCAACTAACTACCAAGATCTAAGAGATACCCGCCTCC  1381481

Query  10321    GCGAGTATGGCAATTTAACTAATGTTTCGAATCAAAAAATTTTTGGTATGCTTCATGGTT  10380
                ||||||||||||||||||||||||||||||||||||||||||||||||||||||||||||
Sbjct  1381482  GCGAGTATGGCAATTTAACTAATGTTTCGAATCAAAAAATTTTTGGTATGCTTCATGGTT  1381541

Query  10381    TTATAGATCTTATTTTTGAGTATGAGGGTAAATTTTATGTTGCTGATTATAAATCAAATT  10440
                ||||||||||||||||||||||||||||||||||||||||||||||||||||||||||||
Sbjct  1381542  TTATAGATCTTATTTTTGAGTATGAGGGTAAATTTTATGTTGCTGATTATAAATCAAATT  1381601

Query  10441    ATCTAGGTGATACTTTAGAAGATTATAATCAGCAGGCGATGCAAGAGAAAAATCAAAGTA  10500
                ||||||||||||||||||||||||||||||||||||||||||||||||||||||||||||
Sbjct  1381602  ATCTAGGTGATACTTTAGAAGATTATAATCAGCAGGCGATGCAAGAGAAAAATCAAAGTA  1381661

Query  10501    GCTTTTATGATTTACAGTATCTAATATATAGCGTTGCCTTGGATAAGTACCTGCGACAAA  10560
                ||||||||||||||||||||||||||||||||||||||||||||||||||||||||||||
Sbjct  1381662  GCTTTTATGATTTACAGTATCTAATATATAGCGTTGCCTTGGATAAGTACCTGCGACAAA  1381721

Query  10561    ACATTGAAAGTTATAATTATGAAAAGCATTTTGGCGGAGTATATTATTTTTATCTTAGAG  10620
                ||||||||||||||||||||||||||||||||||||||||||||||||||||||||||||
Sbjct  1381722  ACATTGAAAGTTATAATTATGAAAAGCATTTTGGCGGAGTATATTATTTTTATCTTAGAG  1381781

Query  10621    GTATGAAAGATGGTTATGGAGTTTATCGAGCTAGACCAAATTTAGAGATTATCAACAAAC  10680
```

```
                  ||||||||||||||||||||||||||||||||||||||||||||||||||||||||||||
Sbjct  1381782  GTATGAAAGATGGTTATGGAGTTTATCGAGCTAGACCAAATTTAGAGATTATCAACAAAC  1381841

Query  10681    TTGCTAGTTTATTTAATGGAGATGATAATGTATAAAACTTTCCATAAAGCTTGTGAGCAA  10740
                ||||||||||||||||||||||||||||||||||||||||||||||||||||||||||||
Sbjct  1381842  TTGCTAGTTTATTTAATGGAGATGATAATGTATAAAACTTTCCATAAAGCTTGTGAGCAA  1381901

Query  10741    TTAGCAGATATTAGAGCCATAGATTTTTTCTTTGCTAAAGAGGTGTTTGATTTAGTCAAA  10800
                ||||||||||||||||||||||||||||||||||||||||||||||||||||||||||||
Sbjct  1381902  TTAGCAGATATTAGAGCCATAGATTTTTTCTTTGCTAAAGAGGTGTTTGATTTAGTCAAA  1381961

Query  10801    AAGACTACTCCGTCTATGACCACTTTATCAAGTGAGTCATATCCACCCTTTCACGGAGCA  10860
                ||||||||||||||||||||||||||||||||||||||||||||||||||||||||||||
Sbjct  1381962  AAGACTACTCCGTCTATGACCACTTTATCAAGTGAGTCATATCCACCCTTTCACGGAGCA  1382021

Query  10861    AAGGGGAATGATTTGGAACAAAAAAGTAATGAAATTTTATTTCATATTTTGATGAAGCTT  10920
                ||||||||||||||||||||||||||||||||||||||||||||||||||||||||||||
Sbjct  1382022  AAGGGGAATGATTTGGAACAAAAAAGTAATGAAATTTTATTTCATATTTTGATGAAGCTT  1382081

Query  10921    ATGCATGTGTATAGTCATGGTCATAGCTGTTTGAAGATAGCAGATATATCAAATAAAACT  10980
                ||||||||||||||||||||||||||||||||||||||||||||||||||||||||||||
Sbjct  1382082  ATGCATGTGTATAGTCATGGTCATAGCTGTTTGAAGATAGCAGATATATCAAATAAAACT  1382141

Query  10981    ATTTTTGCTTCAGAGCAAAGTAATCAGAGTGATGCTAAAGTAGGCTTTAAAATGCCAAGC  11040
                ||||||||||||||||||||||||||||||||||||||||||||||||||||||||||||
Sbjct  1382142  ATTTTTGCTTCAGAGCAAAGTAATCAGAGTGATGCTAAAGTAGGCTTTAAAATGCCAAGC  1382201

Query  11041    TCTGATAAAATTGCAAGTATTTTAGATAACTTAAATTACGATAATTTACCTATATATTTT  11100
                ||||||||||||||||||||||||||||||||||||||||||||||||||||||||||||
Sbjct  1382202  TCTGATAAAATTGCAAGTATTTTAGATAACTTAAATTACGATAATTTACCTATATATTTT  1382261

Query  11101    GCCAAACAGTATGATTCTTTGTATATTAAAAGATTGTGGAATTATGAGAATGAAATAGCT  11160
                ||||||||||||||||||||||||||||||||||||||||||||||||||||||||||||
Sbjct  1382262  GCCAAACAGTATGATTCTTTGTATATTAAAAGATTGTGGAATTATGAGAATGAAATAGCT  1382321

Query  11161    AACTTTATAAAATTAAAAACAATCCAAAATACTCAAAAAGACAATCGTATTGAAGAAGTT  11220
                ||||||||||||||||||||||||||||||||||||||||||||||||||||||||||||
Sbjct  1382322  AACTTTATAAAATTAAAAACAATCCAAAATACTCAAAAAGACAATCGTATTGAAGAAGTT  1382381

Query  11221    GTAGACAAACTATTTGAACCTGCTGGTGAAATCAACTGGCAAAAACAGGCGGTTATCAAA  11280
                ||||||||||||||||||||||||||||||||||||||||||||||||||||||||||||
Sbjct  1382382  GTAGACAAACTATTTGAACCTGCTGGTGAAATCAACTGGCAAAAACAGGCGGTTATCAAA  1382441

Query  11281    AGTCTAAATTATAATTTTAGTATTATCTCAGGTGGACCTGGTACAGGTAAGACAACTACA  11340
                ||||||||||||||||||||||||||||||||||||||||||||||||||||||||||||
Sbjct  1382442  AGTCTAAATTATAATTTTAGTATTATCTCAGGTGGACCTGGTACAGGTAAGACAACTACA  1382501

Query  11341    GTCGCAAAGCTTTTACTTGCTATACAAATGCTTAATCAAAATCAGCAAAGAATAGCGCTT  11400
```

```
               ||||||||||||||||||||||||||||||||||||||||||||||||||||||||||||
Sbjct  1382502  GTCGCAAAGCTTTTACTTGCTATACAAATGCTTAATCAAAATCAGCAAAGAATAGCGCTT  1382561

Query  11401    TTAGCACCAACTGGTAAGGCTGCTCAGAGAATGACAGAGTCATTAAAAAATACTCTAGCA  11460
               ||||||||||||||||||||||||||||||||||||||||||||||||||||||||||||
Sbjct  1382562  TTAGCACCAACTGGTAAGGCTGCTCAGAGAATGACAGAGTCATTAAAAAATACTCTAGCA  1382621

Query  11461    ACTGTCATTCCGTCAAGCTTGACCACAGAATCTACTATACaaaaaaaTGATATCTTTAAA  11520
               ||||||||||||||||||||||||||||||||||||||||||||||||||||||||||||
Sbjct  1382622  ACTGTCATTCCGTCAAGCTTGACCACAGAATCTACTATACAAAAAAATGATATCTTTAAA  1382681

Query  11521    CAAGTTCAGGATGAACTTAATAGTGTTGCTCAGTATCTTAATAATCTAGAAGCACAAACT  11580
               ||||||||||||||||||||||||||||||||||||||||||||||||||||||||||||
Sbjct  1382682  CAAGTTCAGGATGAACTTAATAGTGTTGCTCAGTATCTTAATAATCTAGAAGCACAAACT  1382741

Query  11581    ATACATAGATTTTTAGGCTTAAGACCAAACTCTACTTATGTTAAATATAACCAACAATCT  11640
               ||||||||||||||||||||||||||||||||||||||||||||||||||||||||||||
Sbjct  1382742  ATACATAGATTTTTAGGCTTAAGACCAAACTCTACTTATGTTAAATATAACCAACAATCT  1382801

Query  11641    AAAGCTCCATTCGATGTTATTATTGTTGATGAAGCTTCGATGCTTGATATGAATATTTTT  11700
               ||||||||||||||||||||||||||||||||||||||||||||||||||||||||||||
Sbjct  1382802  AAAGCTCCATTCGATGTTATTATTGTTGATGAAGCTTCGATGCTTGATATGAATATTTTT  1382861

Query  11701    ATCAAGTTAATTAGAGCTGTAGCAGATAATACTAAGTTGATATTAATAGGTGATACTAAT  11760
               ||||||||||||||||||||||||||||||||||||||||||||||||||||||||||||
Sbjct  1382862  ATCAAGTTAATTAGAGCTGTAGCAGATAATACTAAGTTGATATTAATAGGTGATACTAAT  1382921

Query  11761    CAGTTACCATCTGTTGAGGCTGGGAGTCTTTTAGCAAACTTTACACATGATAAATATGGT  11820
               ||||||||||||||||||||||||||||||||||||||||||||||||||||||||||||
Sbjct  1382922  CAGTTACCATCTGTTGAGGCTGGGAGTCTTTTAGCAAACTTTACACATGATAAATATGGT  1382981

Query  11821    AGCATTACGCCATATACAACATTACTTATCAAAAACTATCGTTCACAACAATATATAAAT  11880
               ||||||||||||||||||||||||||||||||||||||||||||||||||||||||||||
Sbjct  1382982  AGCATTACGCCATATACAACATTACTTATCAAAAACTATCGTTCACAACAATATATAAAT  1383041

Query  11881    AATCTTGCAGCCTCAGTCCTAAAAGGCGATGTTAATATTGATAGTCAACAAAATGAAAAT  11940
               ||||||||||||||||||||||||||||||||||||||||||||||||||||||||||||
Sbjct  1383042  AATCTTGCAGCCTCAGTCCTAAAAGGCGATGTTAATATTGATAGTCAACAAAATGAAAAT  1383101

Query  11941    ATTAAATTTCATAGTCTAAGAAATTTAGATATTTATTTAAGAGAGTATGCAAAGAGATAT  12000
               ||||||||||||||||||||||||||||||||||||||||||||||||||||||||||||
Sbjct  1383102  ATTAAATTTCATAGTCTAAGAAATTTAGATATTTATTTAAGAGAGTATGCAAAGAGATAT  1383161

Query  12001    TCACAGCTTGAAAAGTGCCGTGACTACAAAGAGGCTCTTACAGAGCTGAATAAATTTAGA  12060
               ||||||||||||||||||||||||||||||||||||||||||||||||||||||||||||
Sbjct  1383162  TCACAGCTTGAAAAGTGCCGTGACTACAAAGAGGCTCTTACAGAGCTGAATAAATTTAGA  1383221

Query  12061    ATACTTGTGGCTAACAAAAACCTAGAAATTGGTACAGATAAGTTAAATCAGAAAATTGAG  12120
```

```
                 ||||||||||||||||||||||||||||||||||||||||||||||||||||||||||||
Sbjct  1383222   ATACTTGTGGCTAACAAAAACCTAGAAATTGGTACAGATAAGTTAAATCAGAAAATTGAG  1383281

Query  12121     AAGTTTATGTCTAAGCCTATAGATTCTAACTATAAGGGTAAACCAATTATGATAACTCAA  12180
                 ||||||||||||||||||||||||||||||||||||||||||||||||||||||||||||
Sbjct  1383282   AAGTTTATGTCTAAGCCTATAGATTCTAACTATAAGGGTAAACCAATTATGATAACTCAA  1383341

Query  12181     AATAGTTATTCATTAGGGTTATTTAATGGAGATGTAGGGATTATGTGGCCTGATGATACT  12240
                 ||||||||||||||||||||||||||||||||||||||||||||||||||||||||||||
Sbjct  1383342   AATAGTTATTCATTAGGGTTATTTAATGGAGATGTAGGGATTATGTGGCCTGATGATACT  1383401

Query  12241     GGTAAGCTCCGAGCATATTTTGATGGTAAAGATGCTAAGGCATTTAGTCTAAATATGCTA  12300
                 ||||||||||||||||||||||||||||||||||||||||||||||||||||||||||||
Sbjct  1383402   GGTAAGCTCCGAGCATATTTTGATGGTAAAGATGCTAAGGCATTTAGTCTAAATATGCTA  1383461

Query  12301     CCAAAATATGAAAGTGTTTATGCGATGACAATTCATAAAACTCAAGGCTCAGAGTTTGAT  12360
                 ||||||||||||||||||||||||||||||||||||||||||||||||||||||||||||
Sbjct  1383462   CCAAAATATGAAAGTGTTTATGCGATGACAATTCATAAAACTCAAGGCTCAGAGTTTGAT  1383521

Query  12361     GAAATCGTGATTATTCTTCCAGCAGAAGATAATGAAGCGCTAAGTAAACAACTTTTGTAT  12420
                 ||||||||||||||||||||||||||||||||||||||||||||||||||||||||||||
Sbjct  1383522   GAAATCGTGATTATTCTTCCAGCAGAAGATAATGAAGCGCTAAGTAAACAACTTTTGTAT  1383581

Query  12421     ACAGCTATTACTAGAGCAAAATATAAATTAACGATTATATCTGAACAAAGTAGCTTAAGA  12480
                 ||||||||||||||||||||||||||||||||||||||||||||||||||||||||||||
Sbjct  1383582   ACAGCTATTACTAGAGCAAAATATAAATTAACGATTATATCTGAACAAAGTAGCTTAAGA  1383641

Query  12481     GATATTGCTCAGAAAGATATTAATAGAAATTCAAATATCAGTGAATTAGTTAGCTTGAGC  12540
                 ||||||||||||||||||||||||||||||||||||||||||||||||||||||||||||
Sbjct  1383642   GATATTGCTCAGAAAGATATTAATAGAAATTCAAATATCAGTGAATTAGTTAGCTTGAGC  1383701

Query  12541     ACTCAATAACTTAGAAATTACTTATTCATTTGCCAGATTATTCTAAGTCCATGAAGTAAT  12600
                 ||||||||||||||||||||||||||||||||||||||||||||||||||||||||||||
Sbjct  1383702   ACTCAATAACTTAGAAATTACTTATTCATTTGCCAGATTATTCTAAGTCCATGAAGTAAT  1383761

Query  12601     AAATCAGAGATGATTACATCAAAATATTGTTCTTTCTCAAAAATATGAGCATAGCCTCCA  12660
                 ||||||||||||||||||||||||||||||||||||||||||||||||||||||||||||
Sbjct  1383762   AAATCAGAGATGATTACATCAAAATATTGTTCTTTCTCAAAAATATGAGCATAGCCTCCA  1383821

Query  12661     GTTGCTATTAATATTGGTGGTTTATCTATGAAGTTTTCTTGAGATATTCGATTAATTATC  12720
                 ||||||||||||||||||||||||||||||||||||||||||||||||||||||||||||
Sbjct  1383822   GTTGCTATTAATATTGGTGGTTTATCTATGAAGTTTTCTTGAGATATTCGATTAATTATC  1383881

Query  12721     TCTTTTATAGCACCTAATTGACCATATATAAGACCCGATTGGATTTGAGAGATAGTTGTC  12780
                 ||||||||||||||||||||||||||||||||||||||||||||||||||||||||||||
Sbjct  1383882   TCTTTTATAGCACCTAATTGACCATATATAAGACCCGATTGGATTTGAGAGATAGTTGTC  1383941

Query  12781     TTACCTAACGCAGAACTAGGGTGAGAGATAGTTACATTAGATAGTTTTGCAGTTTTTTGT  12840
```

```
              ||||||||||||||||||||||||||||||||||||||||||||||||||||||||||||
Sbjct  1383942  TTACCTAACGCAGAACTAGGGTGAGAGATAGTTACATTAGATAGTTTTGCAGTTTTTTGT  1384001

Query  12841    GATAAGCTCTCCATTGATAGGTTAATTCCTGGTAGGATAGCACCGCCGATATAGGTTTTA  12900
                ||||||||||||||||||||||||||||||||||||||||||||||||||||||||||||
Sbjct  1384002  GATAAGCTCTCCATTGATAGGTTAATTCCTGGTAGGATAGCACCGCCGATATAGGTTTTA  1384061

Query  12901    TTCTCTGAAATAGCACAAATAGTTGTTGCTGTGCCAAAGTCAACTACAATAATATTTCTC  12960
                ||||||||||||||||||||||||||||||||||||||||||||||||||||||||||||
Sbjct  1384062  TTCTCTGAAATAGCACAAATAGTTGTTGCTGTGCCAAAGTCAACTACAATAATATTTCTC  1384121

Query  12961    GATGGAAAAAGAGAAATAGCTGCAACTGAGTTTGCAACACGATCTGCACCTAAATCTAGA  13020
                ||||||||||||||||||||||||||||||||||||||||||||||||||||||||||||
Sbjct  1384122  GATGGAAAAAGAGAAATAGCTGCAACTGAGTTTGCAACACGATCTGCACCTAAATCTAGA  1384181

Query  13021    GGATTTTTGATATCGAGTTTTAATCCTGTTTTTACCCCAGGTTTTAGCTCAAGCGGAGTT  13080
                ||||||||||||||||||||||||||||||||||||||||||||||||||||||||||||
Sbjct  1384182  GGATTTTTGATATCGAGTTTTAATCCTGTTTTTACCCCAGGTTTTAGCTCAAGCGGAGTT  1384241

Query  13081    ATACCTAAATACTTTTTGCAAGCGGAATTGACAGAATACTCTAGATGAAGAACAACAGAT  13140
                ||||||||||||||||||||||||||||||||||||||||||||||||||||||||||||
Sbjct  1384242  ATACCTAAATACTTTTTGCAAGCGGAATTGACAGAATACTCTAGATGAAGAACAACAGAT  1384301

Query  13141    GAAAGTACAACTGCTTCAATATCTTCGATATCAAGCTTTTTCCTCTCAAAGAACGATAGC  13200
                ||||||||||||||||||||||||||||||||||||||||||||||||||||||||||||
Sbjct  1384302  GAAAGTACAACTGCTTCAATATCTTCGATATCAAGCTTTTTCCTCTCAAAGAACGATAGC  1384361

Query  13201    AGAAATATACCTAGCGTATCAGAAGTACACGGAGTAGTAGAAGGGTAGCGAAAGTTATGC  13260
                ||||||||||||||||||||||||||||||||||||||||||||||||||||||||||||
Sbjct  1384362  AGAAATATACCTAGCGTATCAGAAGTACACGGAGTAGTAGAAGGGTAGCGAAAGTTATGC  1384421

Query  13261    TTAATTTGATCTCCTACAAATACTCCACCAAAAAATATGTGAGTTACCAATATCTATACAT  13320
                ||||||||||||||||||||||||||||||||||||||||||||||||||||||||||||
Sbjct  1384422  TTAATTTGATCTCCTACAAATACTCCACCAAAAAATATGTGAGTTACCAATATCTATACAT  1384481

Query  13321    ACTATCAT  13328
                ||||||||
Sbjct  1384482  ACTATCAT  1384489
```
