# Supplementary material for: Development and Properties of Francisella tularensis Subsp. holarctica 15 NIIEG Vaccine Strain without the recD Gene
Source: Vaccines (Basel). 2022 Jan 11;10(1):108. doi: 10.3390/vaccines10010108 (PMC8778684; doi:10.3390/vaccines10010108)
Supplement: Supplementary file 1 [file vaccines-10-00108-s001.zip › Supplementary S3.pdf]

Supplementary 3.

Multiple alignment of the amino acid sequence of the RecD proteins of the genus Francisella

Strain	RecD protein accession number
F. t. subsp. tularensis WY96-3418	ABO46414.1
F.t. subsp.holarctica LVS	AJI59740.1
F.t.subsp.holarctica FTNF002-00	ABU61181.1
F. t.subsp.mediasiatica FSC147	ACD30618.1
F.philomiragia ATCC 25017	AJI55965.1
F.t.subsp novicida U112	ABK90231.1
F.t.subsp. holarctica OSU18	ABI82625.1
F.t.subsp.tularensis FSC198	CAL09409.1
F.t.subsp.tularensis SCHU S4	CAG46026.1

```
                                                 1                                                50
 F. t. subsp. tularensis WY96-3418 RecD     (1) ---------MYKTFHKACEQLADIRAIDFFFAKEVFDLVKKTTPSMTTLS
          F.t. subsp.holarctica LVS RecD     (1) ---------MYKTFHKACEQLADIRAIDFFFAKEVFDLVKKTTPSMTTLS
   F.t.subsp.holarctica FTNF002-00 RecD     (1) ---------MYKTFHKACEQLADIRAIDFFFAKEVFDLVKKTTPSMTTLS
     F. t.subsp.mediasiatica FSC147 RecD     (1) MLVYLMEMIMYKTFHKACEQLADIRAIDFFFAKEVFDLVKKTTPSMTTLS
          F.philomiragia ATCC 25017 RecD     (1) ---------MYKTFHQACEQLADIRAIDFFFAKEVFDLVKKTTPSIITLS
            F.t.subsp novicida U112  RecD     (1) MLVYLMEMIMYKTFHKACEQLADIRAIDFFFAKEVFDLVKKTTPSMTTLS
         F.t.subsp. holarctica OSU18 RecD     (1) MLVYLMEMIMYKTFHKACEQLADIRAIDFFFAKEVFDLVKKTTPSMTTLS
        F.t.subsp.tularensis FSC198 RecD     (1) MLVYLMEMIMYKTFHKACEQLADIRAIDFFFAKEVFGLVKKTTPSMTTLS
       F.t.subsp.tularensis SCHU S4 RecD     (1) MLVYLMEMIMYKTFHKACEQLADIRAIDFFFAKEVFGLVKKTTPSMTTLS
                               Consensus     (1) MLVYLMEMIMYKTFHKACEQLADIRAIDFFFAKEVFDLVKKTTPSMTTLS
                                                 51                                              100
 F. t. subsp. tularensis WY96-3418 RecD    (42) SESYLPFHGAKGNDLEQKSNEILFHILMKLMHVYSHGHSCLKIADISNKT
          F.t. subsp.holarctica LVS RecD    (42) SESYPPFHGAKGNDLEQKSNEILFHILMKLMHVYSHGHSCLKIADISNKT
   F.t.subsp.holarctica FTNF002-00 RecD    (42) SESYPPFHGAKGNDLEQKSNEILFHILMKLMHVYSHGHSCLKIADISNKT
     F. t.subsp.mediasiatica FSC147 RecD    (51) SESYPPFHGAKGNDLEQKSNEILFHILMKLMHVYSHGHSCLKIADISNKT
          F.philomiragia ATCC 25017 RecD    (42) SELYPPLHGAKGNDLEQKSNEVLFHILIKLMHVYSHGHSCLKIADISNKT
            F.t.subsp novicida U112  RecD    (51) SESYPPLHGAKGNDLEQKSNEILFHILMKLMHVYSHGHSCLKIADISNKT
         F.t.subsp. holarctica OSU18 RecD    (51) SESYPPFHGAKGNDLEQKSNEILFHILMKLMHVYSHGHSCLKIADISNKT
        F.t.subsp.tularensis FSC198 RecD    (51) SESYPPFHGAKGNDLEQKSNEILFHILMKLMHVYSHGHSCLKIADISNKT
       F.t.subsp.tularensis SCHU S4 RecD    (51) SESYPPFHGAKGNDLEQKSNEILFHILMKLMHVYSHGHSCLKIADISNKT
                               Consensus    (51) SESYPPFHGAKGNDLEQKSNEILFHILMKLMHVYSHGHSCLKIADISNKT
                                                 101                                             150
 F. t. subsp. tularensis WY96-3418 RecD    (92) IFASEQSNQSDAKVGFKMPSSDKIASILDNLNYDNLPIYFAKQYDSLYIK
          F.t. subsp.holarctica LVS RecD    (92) IFASEQSNQSDAKVGFKMPSSDKIASILDNLNYDNLPIYFAKQYDSLYIK
   F.t.subsp.holarctica FTNF002-00 RecD    (92) IFASEQSNQSDAKVGFKMPSSDKIASILDNLNYDNLPIYFAKQYDSLYIK
     F. t.subsp.mediasiatica FSC147 RecD   (101) IFASEQSNQSDAKVGFKMPSSDKIASILDNLNYDNLPIYFAKQYDSLYIK
          F.philomiragia ATCC 25017 RecD    (92) IFASEQSTQSDAKVGFKMPSFDEIVSILESLDYDKLPIYFAKEYDSLYIK
            F.t.subsp novicida U112  RecD   (101) IFASEQSNQSDAKVGFKMPSSDKIANILDSLNYDNLPIYFAKQYDSLYIK
```

```
       F.t.subsp. holarctica OSU18 RecD  (101)  IFASEQSNQSDAKVGFKMPSSDKIASILDNLNYDNLPIYFAKQYDSLYIK
       F.t.subsp.tularensis FSC198 RecD  (101)  IFASEQSNQSDAKVGFKMPSSDKIASILDNLNYDNLPIYFAKQYDSLYIK
     F.t.subsp.tularensis SCHU S4 RecD  (101)  IFASEQSNQSDAKVGFKMPSSDKIASILDNLNYDNLPIYFAKQYDSLYIK
                              Consensus  (101)  IFASEQSNQSDAKVGFKMPSSDKIASILDNLNYDNLPIYFAKQYDSLYIK
                                                151                                              200
 F. t. subsp. tularensis WY96-3418 RecD  (142)  RLWNYENEMANFIKLKTIQNTQKDNRIEEVVDKLFEPAGEINWQKQAVIK
            F.t. subsp.holarctica LVS RecD  (142)  RLWNYENEIANFIKLKTIQNTQKDNRIEEVVDKLFEPAGEINWQKQAVIK
    F.t.subsp.holarctica FTNF002-00 RecD  (142)  RLWNYENEIANFIKLKTIQNTQKDNRIEEVVDKLFEPAGEINWQKQAVIK
      F. t.subsp.mediasiatica FSC147 RecD  (151)  RLWNYENEIANFIKLKTIQNTQKDNRIEEVVDKLFEPAGEINWQKQAVIK
          F.philomiragia ATCC 25017 RecD  (142)  RLWNYEVEIANFIKSRTIQNIQQANNIEEVVDKLFEPADEIDWQKQAVIK
            F.t.subsp novicida U112  RecD  (151)  RLWNYENEIANFIKLKTIQNTQKDNRIEEVVDKLFEPAGEINWQKQAVIK
       F.t.subsp. holarctica OSU18 RecD  (151)  RLWNYENEIANFIKLKTIQNTQKDNRIEEVVDKLFEPAGEINWQKQAVIK
       F.t.subsp.tularensis FSC198 RecD  (151)  RLWNYENEIANFIKLKTIQNTQKDNRIEEVVDKLFEPAGEINWQKQAVIK
     F.t.subsp.tularensis SCHU S4 RecD  (151)  RLWNYENEIANFIKLKTIQNTQKDNRIEEVVDKLFEPAGEINWQKQAVIK
                              Consensus  (151)  RLWNYENEIANFIKLKTIQNTQKDNRIEEVVDKLFEPAGEINWQKQAVIK

                                                201                                              250
 F. t. subsp. tularensis WY96-3418 RecD  (192)  SLNYNFSIISGGPGTGKTTTVAKLLLAIQMINQNQQRIALLAPTGKAAQR
            F.t. subsp.holarctica LVS RecD  (192)  SLNYNFSIISGGPGTGKTTTVAKLLLAIQMINQNQQRIALLAPTGKAAQR
    F.t.subsp.holarctica FTNF002-00 RecD  (192)  SLNYNFSIISGGPGTGKTTTVAKLLLAIQMINQNQQRIALLAPTGKAAQR
      F. t.subsp.mediasiatica FSC147 RecD  (201)  SLNYNFSIISGGPGTGKTTTVAKLLLAIQMINQNQQRIALLAPTGKAAQR
          F.philomiragia ATCC 25017 RecD  (192)  SLNYNFSIISGGPGTGKTTTVAKLLLAIQMSNQNQQRIALLAPTGKAAQR
            F.t.subsp novicida U112  RecD  (201)  SLNYNFSIISGGPGTGKTTTVAKLLLAIQMLNQNQQRIALLAPTGKAAQR
       F.t.subsp. holarctica OSU18 RecD  (201)  SLNYNFSIISGGPGTGKTTTVAKLLLAIQMLNQNQQRIALLAPTGKAAQR
       F.t.subsp.tularensis FSC198 RecD  (201)  SLNYNFSIISGGPGTGKTTTVAKLLLAIQMLNQNQQRIALLAPTGKAAQR
     F.t.subsp.tularensis SCHU S4 RecD  (201)  SLNYNFSIISGGPGTGKTTTVAKLLLAIQMLNQNQQRIALLAPTGKAAQR
                              Consensus  (201)  SLNYNFSIISGGPGTGKTTTVAKLLLAIQMLNQNQQRIALLAPTGKAAQR
                                                251                                              300
 F. t. subsp. tularensis WY96-3418 RecD  (242)  MTESLKNTLATVIPSSLTTESTIQKNDIFKQVQDELNSVAQYLNNLEAQT
            F.t. subsp.holarctica LVS RecD  (242)  MTESLKNTLATVIPSSLTTESTIQKNDIFKQVQDELNSVAQYLNNLEAQT
    F.t.subsp.holarctica FTNF002-00 RecD  (242)  MTESLKNTLATVIPSSLTTESTIQKNDIFKQVQDELNSVAQYLNNLEAQT
      F. t.subsp.mediasiatica FSC147 RecD  (251)  MTESLKNTLATVIPSSLTTESTIQKNDIFKQIQDELNSVAQYLNNLEAQT
          F.philomiragia ATCC 25017 RecD  (242)  MRESLNSVLSKRVDNDSYLG----------------------LSNLEAQT
            F.t.subsp novicida U112  RecD  (251)  MTESLNSVLSKRVDNDSYLG----------------------LSNLEAQT
       F.t.subsp. holarctica OSU18 RecD  (251)  MTESLKNTLATVIPSSLTTESTIQKNDIFKQVQDELNSVAQYLNNLEAQT
       F.t.subsp.tularensis FSC198 RecD  (251)  MTESLKNTLATVIPSSLTTESTIQKNDIFKQVQDELNSVAQYLNNLEAQT
     F.t.subsp.tularensis SCHU S4 RecD  (251)  MTESLKNTLATVIPSSLTTESTIQKNDIFKQVQDELNSVAQYLNNLEAQT
                              Consensus  (251)  MTESLKNTLATVIPSSLTTESTIQKNDIFKQVQDELNSVAQYLNNLEAQT
                                                301                                              350
 F. t. subsp. tularensis WY96-3418 RecD  (292)  IHRFLGLRPNSTYVKYNQQSKAPFDVIIVDEASMLDMNIFIKLIRAVADN
            F.t. subsp.holarctica LVS RecD  (292)  IHRFLGLRPNSTYVKYNQQSKAPFDVIIVDEASMLDMNIFIKLIRAVADN
```

```
F.t.subsp.holarctica FTNF002-00 RecD  (292) IHRFLGLRPNSTYVKYNQQSKAPFDVIIVDEASMLDMNIFIKLIRAVADN
F. t.subsp.mediasiatica FSC147 RecD  (301) IHRFLGLRPNSTYVKYNQQSKAPFDVIIVDEASMLDMNIFIKLIRAVADN
F.philomiragia ATCC 25017 RecD       (270) IHRFLGLRPNSTHVKYNEQSKAPYDVIIVDEASMLDMNIFIKLIRAVADN
F.t.subsp novicida U112  RecD        (279) IHRFLGLRPNSTHVKYSKQSKAPYDVIIVDEASMLDMNIFIKLIRAVADN
F.t.subsp. holarctica OSU18 RecD     (301) IHRFLGLRPNSTYVKYNQQSKAPFDVIIVDEASMLDMNIFIKLIRAVADN
F.t.subsp.tularensis FSC198 RecD     (301) IHRFLGLRPNSTYVKYNQQSKAPFDVIIVDEASMLDMNIFIKLIRAVADN
F.t.subsp.tularensis SCHU S4 RecD    (301) IHRFLGLRPNSTYVKYNQQSKAPFDVIIVDEASMLDMNIFIKLIRAVADN
Consensus                            (301) IHRFLGLRPNSTYVKYNQQSKAPFDVIIVDEASMLDMNIFIKLIRAVADN
                                           351                                              400
F. t. subsp. tularensis WY96-3418 RecD (342) TKLILIGDTNQLPSVEAGSLLANFTHDKYGSITPYTTLLIKNYRSQQYIN
F.t. subsp.holarctica LVS RecD        (342) TKLILIGDTNQLPSVEAGSLLANFTHDKYGSITPYTTLLIKNYRSQQYIN
F.t.subsp.holarctica FTNF002-00 RecD  (342) TKLILIGDTNQLPSVEAGSLLANFTHDKYGSITPYTTLLIKNYRSQQYIN
F. t.subsp.mediasiatica FSC147 RecD  (351) TKLILIGDTNQLPSVEAGSLLANFTHDKYGSITPYTTLLIKNYRSQQYIN
F.philomiragia ATCC 25017 RecD       (320) TKLILIGDTNQLPSVEAGSLLANFTHHRDGDITPYTTLLIKNYRSQQYIN
F.t.subsp novicida U112  RecD        (329) TKLILIGDTNQLPSVEAGSLLANFTHDKYGSITPYTTLLIKNYRSQQYIN
F.t.subsp. holarctica OSU18 RecD     (351) TKLILIGDTNQLPSVEAGSLLANFTHDKYGSITPYTTLLIKNYRSQQYIN
F.t.subsp.tularensis FSC198 RecD     (351) TKLILIGDTNQLPSVEAGSLLANFTHDKYGSITPYTTLLIKNYRSQQYIN
F.t.subsp.tularensis SCHU S4 RecD    (351) TKLILIGDTNQLPSVEAGSLLANFTHDKYGSITPYTTLLIKNYRSQQYIN
Consensus                            (351) TKLILIGDTNQLPSVEAGSLLANFTHDKYGSITPYTTLLIKNYRSQQYIN
                                           401                                              450
F. t. subsp. tularensis WY96-3418 RecD (392) NLAASVLKGDVNIDSQQNENIKFHSLRNLDIYLREYAKRYSQLEKCRDYK
F.t. subsp.holarctica LVS RecD        (392) NLAASVLKGDVNIDSQQNENIKFHSLRNLDIYLREYAKRYSQLEKCRDYK
F.t.subsp.holarctica FTNF002-00 RecD  (392) NLAASVLKGDVNIDSQQNENIKFHSLRNLDIYLREYAKRYSQLEKCRDYK
F. t.subsp.mediasiatica FSC147 RecD  (401) NLAASVLKGDVNIDSQQNENIKFHSLRNLDIYLREYAKRYSQLEKCRDYK
F.philomiragia ATCC 25017 RecD       (370) NLAASVLKGDINTDNHQNENINFHSIRNLDTYLREYAKRYSQLEKCRDYK
F.t.subsp novicida U112  RecD        (379) NLAASVLKGDVNIDSHQNENINFHSLRNLDTYLREYAKRYSQLEKCRNYK
F.t.subsp. holarctica OSU18 RecD     (401) NLAASVLKGDVNIDSQQNENIKFHSLRNLDIYLREYAKRYSQLEKCRDYK
F.t.subsp.tularensis FSC198 RecD     (401) NLAASVLKGDVNIDSQQNENIKFHSLRNLDIYLREYAKRYSQLEKCRDYK
F.t.subsp.tularensis SCHU S4 RecD    (401) NLAASVLKGDVNIDSQQNENIKFHSLRNLDIYLREYAKRYSQLEKCRDYK
Consensus                            (401) NLAASVLKGDVNIDSQQNENIKFHSLRNLDIYLREYAKRYSQLEKCRDYK
                                           451                                              500
F. t. subsp. tularensis WY96-3418 RecD (442) EALTELNKFRILVANKNLEIGTDKLNQKIEKFMSKPIDSNYKGKPIMITQ
F.t. subsp.holarctica LVS RecD        (442) EALTELNKFRILVANKNLEIGTDKLNQKIEKFMSKPIDSNYKGKPIMITQ
F.t.subsp.holarctica FTNF002-00 RecD  (442) EALTELNKFRILVANKNLEIGTDKLNQKIEKFMSKPIDSNYKGKPIMITQ
F. t.subsp.mediasiatica FSC147 RecD  (451) EALTELNKFRILVANKNLEIGTDKLNQKIEKFMSKPIDSNYKGKPIMITQ
F.philomiragia ATCC 25017 RecD       (420) EALTELNKFRILVANKNLEIGTDKLNQKIEKFMSKPIDSNYKGKPIMITQ
F.t.subsp novicida U112  RecD        (429) EALTELNKFRILVANKNLEIGTDKLNQKIEKFMSKPIDSNYKGKPIMITQ
F.t.subsp. holarctica OSU18 RecD     (451) EALTELNKFRILVANKNLEIGTDKLNQKIEKFMSKPIDSNYKGKPIMITQ
F.t.subsp.tularensis FSC198 RecD     (451) EALTELNKFRILVANKNLEIGTDKLNQKIEKFMSKPIDSNYKGKLIMITQ
F.t.subsp.tularensis SCHU S4 RecD    (451) EALTELNKFRILVANKNLEIGTDKLNQKIEKFMSKPIDSNYKGKLIMITQ
Consensus                            (451) EALTELNKFRILVANKNLEIGTDKLNQKIEKFMSKPIDSNYKGKPIMITQ
                                           501                                              550
F. t. subsp. tularensis WY96-3418 RecD (492) NSYSLGLFNGDVGIMWPDDTGKLRAYFDGKDAKAFSLNMLPKYESVYAMT
```

F.t. subsp.holarctica LVS RecD	(492)	NSYSLGLFNGDVGIMWPDDTGKLRAYFDGKDAKAFSLNMLPKYESVYAMT
F.t.subsp.holarctica FTNF002-00 RecD	(492)	NSYSLGLFNGDVGIMWPDDTGKLRAYFDGKDAKAFSLNMLPKYESVYAMT
F. t.subsp.mediasiatica FSC147 RecD	(501)	NSYSLGLFNGDVGIMWPDDTGKLRVYFDGKDAKAFSLNMLPKYESVYAMT
F.philomiragia ATCC 25017 RecD	(470)	NSYSLGLFNGDVGIIWPDDTGKLRAYFDGKDAKAFSLNMLPKYESVYAMT
F.t.subsp novicida U112 RecD	(479)	NSYSLGLFNGDVGIIWPDDTGKLRAYFDGKDAKAFSLNMLPKYESVYAMT
F.t.subsp. holarctica OSU18 RecD	(501)	NSYSLGLFNGDVGIMWPDDTGKLRAYFDGKDAKAFSLNMLPKYESVYAMT
F.t.subsp.tularensis FSC198 RecD	(501)	NSYSLGLFNGDVGIMWPDDTGKLRAYFDGKDAKAFSLNMLPKYESVYAMT
F.t.subsp.tularensis SCHU S4 RecD	(501)	NSYSLGLFNGDVGIMWPDDTGKLRAYFDGKDAKAFSLNMLPKYESVYAMT
Consensus	(501)	NSYSLGLFNGDVGIMWPDDTGKLRAYFDGKDAKAFSLNMLPKYESVYAMT

		551 … 600
F. t. subsp. tularensis WY96-3418 RecD	(542)	IHKTQGSEFDEIVIILPAEDNEALSKQLLYTAITRAKYKLTIISEQSSLR
F.t. subsp.holarctica LVS RecD	(542)	IHKTQGSEFDEIVIILPAEDNEALSKQLLYTAITRAKYKLTIISEQSSLR
F.t.subsp.holarctica FTNF002-00 RecD	(542)	IHKTQGSEFDEIVIILPAEDNEALSKQLLYTAITRAKYKLTIISEQSSLR
F. t.subsp.mediasiatica FSC147 RecD	(551)	IHKTQGSEFDEIVIILPAEDNEALSKQLLYTAITRAKYKLTIISEQSSLR
F.philomiragia ATCC 25017 RecD	(520)	IHKTQGSEFDEIVIILPAEDNEALCKQLLYTAITRAKHKLTIISEQSSLR
F.t.subsp novicida U112 RecD	(529)	IHKTQGSEFDEIVIILPAEDNEALSKQLLYTAITRAKYKLTIISEQSSLR
F.t.subsp. holarctica OSU18 RecD	(551)	IHKTQGSEFDEIVIILPAEDNEALSKQLLYTAITRAKYKLTIISEQSSLR
F.t.subsp.tularensis FSC198 RecD	(551)	IHKTQGSEFDEIVIILPAEDNEALSKQLLYTAITRAKYKLTIISEQSSLR
F.t.subsp.tularensis SCHU S4 RecD	(551)	IHKTQGSEFDEIVIILPAEDNEALSKQLLYTAITRAKYKLTIISEQSSLR
Consensus	(551)	IHKTQGSEFDEIVIILPAEDNEALSKQLLYTAITRAKYKLTIISEQSSLR

		601 … 622
F. t. subsp. tularensis WY96-3418 RecD	(592)	DIAQKDIKRNSNISELVSLSTQ
F.t. subsp.holarctica LVS RecD	(592)	DIAQKDINRNSNISELVSLSTQ
F.t.subsp.holarctica FTNF002-00 RecD	(592)	DIAQKDINRNSNISELVSLSTQ
F. t.subsp.mediasiatica FSC147 RecD	(601)	DIAQKDIKRNSNISELVSLSTQ
F.philomiragia ATCC 25017 RecD	(570)	DIAQKDIKRNSNISELVSLNN-
F.t.subsp novicida U112 RecD	(579)	DIAQKDIKRNSNISELVSLSD-
F.t.subsp. holarctica OSU18 RecD	(601)	DIAQKDIKRNSNISELVSLSTQ
F.t.subsp.tularensis FSC198 RecD	(601)	DIAQKDIKRNSNISELVSLSTQ
F.t.subsp.tularensis SCHU S4 RecD	(601)	DIAQKDIKRNSNISELVSLSTQ
Consensus	(601)	DIAQKDIKRNSNISELVSLSTQ
