# Supplementary material for: Development and Properties of Francisella tularensis Subsp. holarctica 15 NIIEG Vaccine Strain without the recD Gene
Source: Vaccines (Basel). 2022 Jan 11;10(1):108. doi: 10.3390/vaccines10010108 (PMC8778684; doi:10.3390/vaccines10010108)

Supplementary 4. Electrophoregram of amplicons obtained on FCD/RCD primers and *F. tularensis* DNA: 15 NIIEG – line 2, 15D – line 3. 100 bp plusDNA ladder (Fermentas)– line 1 and 4.

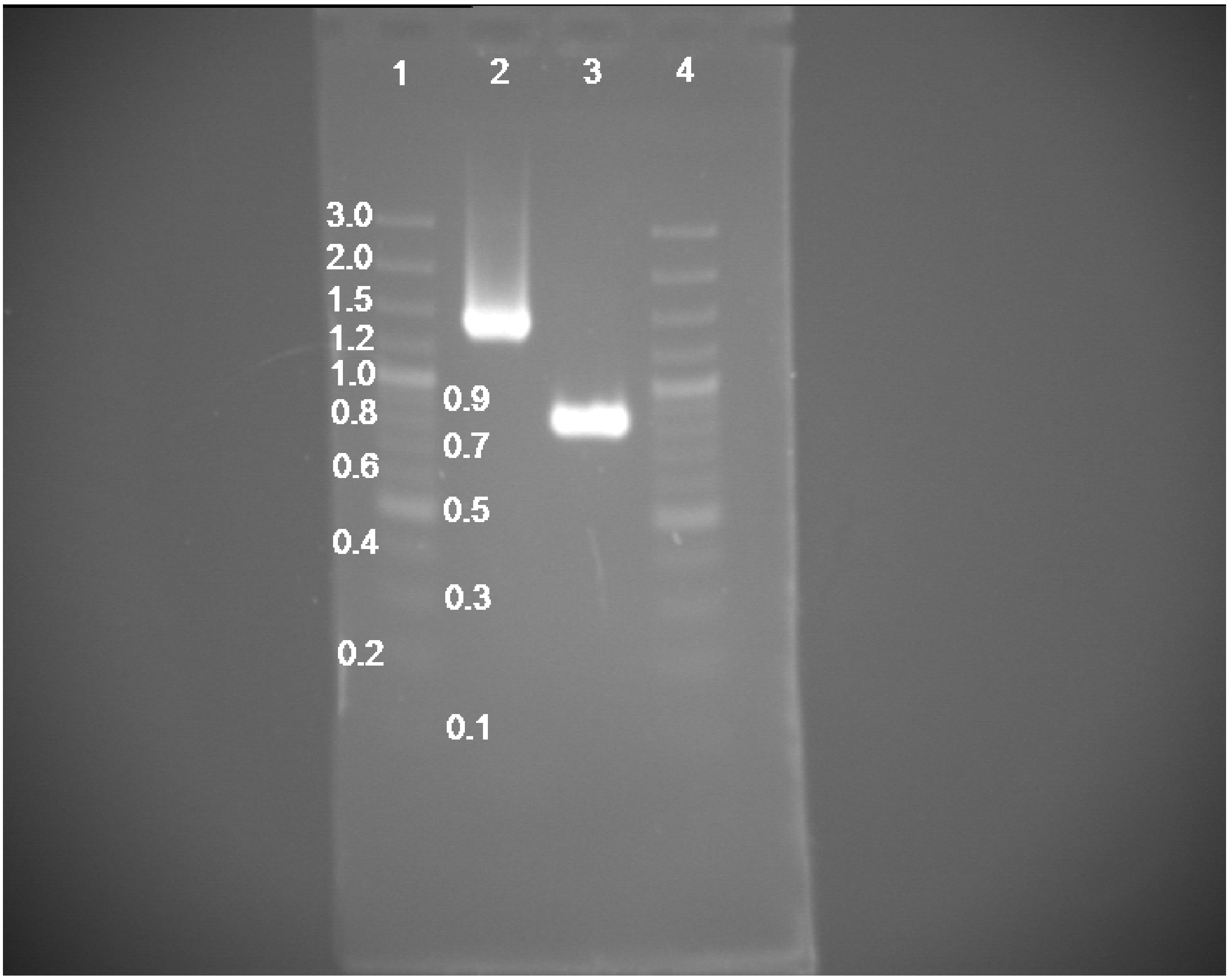

Supplement: Supplementary file 1 [file vaccines-10-00108-s001.zip › Supplementary S4.pdf]
