# Supplementary material for: Development and Properties of Francisella tularensis Subsp. holarctica 15 NIIEG Vaccine Strain without the recD Gene
Source: Vaccines (Basel). 2022 Jan 11;10(1):108. doi: 10.3390/vaccines10010108 (PMC8778684; doi:10.3390/vaccines10010108)
Supplement: Supplementary file 1 [file vaccines-10-00108-s001.zip › Supplementary S5.pdf]

**Supplementary 5. Comparison of the *recBCD* regions of *Francisella tularensis* subsp. *holarctica* LVS and *Francisella tularensis* subsp. *holarctica* 15D**

**Francisella tularensis subsp. holarctica LVS, complete genome**
**Sequence ID: CP009694.1 Length: 1892177 Number of Matches: 2**
Range 1: 1371162 to 1381979 GenBankGraphicsNext MatchPrevious Match

**Query - Francisella tularensis subsp. holarctica 15D**

**Alignment statistics for match #1**

| Score | Expect | Identities | Gaps |
|---|---|---|---|
| 19978 bits(10818) | 0.0 | 10818/10818(100%) | 0/10818(0%) |

```
Query  1        ATGCATATAAGTGAGCGCCGTAAATGGTATATAGGCTTTGCTGTTTTTGTGATGGTGATT  60
                ||||||||||||||||||||||||||||||||||||||||||||||||||||||||||||
Sbjct  1371162  ATGCATATAAGTGAGCGCCGTAAATGGTATATAGGCTTTGCTGTTTTTGTGATGGTGATT  1371221

Query  61       TTATTATCGCTTTCACTACTTCATGCTTCAATATGGTTTAGTATAGGGTTTGTCGCTGTT  120
                ||||||||||||||||||||||||||||||||||||||||||||||||||||||||||||
Sbjct  1371222  TTATTATCGCTTTCACTACTTCATGCTTCAATATGGTTTAGTATAGGGTTTGTCGCTGTT  1371281

Query  121      TTGGCAGTGATCGCAATCTATGATGTTAGCCAAACAAAGCATTCCATCCTAAGAAACTTT  180
                ||||||||||||||||||||||||||||||||||||||||||||||||||||||||||||
Sbjct  1371282  TTGGCAGTGATCGCAATCTATGATGTTAGCCAAACAAAGCATTCCATCCTAAGAAACTTT  1371341

Query  181      CCTATAGTAGGACATATGCGCTATATACTTGAGTTTTTACGTCCTGAAATTCAGCAGTAT  240
                ||||||||||||||||||||||||||||||||||||||||||||||||||||||||||||
Sbjct  1371342  CCTATAGTAGGACATATGCGCTATATACTTGAGTTTTTACGTCCTGAAATTCAGCAGTAT  1371401

Query  241      TTCATTGCTGATAATGAGAGTGAAAAGCCATTTGGTAGAGAGCTACGTTCGACAATCTAC  300
                ||||||||||||||||||||||||||||||||||||||||||||||||||||||||||||
Sbjct  1371402  TTCATTGCTGATAATGAGAGTGAAAAGCCATTTGGTAGAGAGCTACGTTCGACAATCTAC  1371461

Query  301      CGTCGTGCTAAGGGTATTAATGATACTGTTGCTTTTGGAACAGAGAAAGATATTTATAGG  360
                ||||||||||||||||||||||||||||||||||||||||||||||||||||||||||||
Sbjct  1371462  CGTCGTGCTAAGGGTATTAATGATACTGTTGCTTTTGGAACAGAGAAAGATATTTATAGG  1371521

Query  361      GTTGGTTATGAATGGGTAACACATTCACTCATGCCTAAGCATCTTGATGAAATTGAAACT  420
                ||||||||||||||||||||||||||||||||||||||||||||||||||||||||||||
Sbjct  1371522  GTTGGTTATGAATGGGTAACACATTCACTCATGCCTAAGCATCTTGATGAAATTGAAACT  1371581

Query  421      CGAGTTAAAATTGGTGGTTCTGATTGTAAACAACCATATATGGCATCGCATCTAAATATA  480
                ||||||||||||||||||||||||||||||||||||||||||||||||||||||||||||
Sbjct  1371582  CGAGTTAAAATTGGTGGTTCTGATTGTAAACAACCATATATGGCATCGCATCTAAATATA  1371641

Query  481      TCTGCGATGAGTTTTGGGGCATTATCTGCTAATGCCGTTATGGCTTTGAATAAGGGAGCT  540
                ||||||||||||||||||||||||||||||||||||||||||||||||||||||||||||
Sbjct  1371642  TCTGCGATGAGTTTTGGGGCATTATCTGCTAATGCCGTTATGGCTTTGAATAAGGGAGCT  1371701

Query  541      AAACTTGGAGGGTTTTATCAATGTACAGGTGAGGGCGGCTTGACTAAATATCATTTACAA  600
```

```
                    ||||||||||||||||||||||||||||||||||||||||||||||||||||||||||||
Sbjct  1371702  AAACTTGGAGGGTTTTATCAATGTACAGGTGAGGGCGGCTTGACTAAATATCATTTACAA  1371761

Query  601      GGTGGAGATCTTGTATTTCAAATTGGTACAGGCTATTTTGGCTGTCGTACTGATGATGGC  660
                ||||||||||||||||||||||||||||||||||||||||||||||||||||||||||||
Sbjct  1371762  GGTGGAGATCTTGTATTTCAAATTGGTACAGGCTATTTTGGCTGTCGTACTGATGATGGC  1371821

Query  661      AAGTTCTCAGCAGAGAAATTTGTTGAAAAAGCAAATTTAGATAGAGTCAAAATGATAGAA  720
                ||||||||||||||||||||||||||||||||||||||||||||||||||||||||||||
Sbjct  1371822  AAGTTCTCAGCAGAGAAATTTGTTGAAAAAGCAAATTTAGATAGAGTCAAAATGATAGAA  1371881

Query  721      ATTAAGTTATCTCAAGGGGCTAAACCATCTCATGGCGGGGTTTTGCCAGCAGCTAAGATA  780
                ||||||||||||||||||||||||||||||||||||||||||||||||||||||||||||
Sbjct  1371882  ATTAAGTTATCTCAAGGGGCTAAACCATCTCATGGCGGGGTTTTGCCAGCAGCTAAGATA  1371941

Query  781      ACTCCAGAAATTGCTGAAATTAGGGGTGTTTCAATGGGCAAAGATGTACTATCACCTCCT  840
                ||||||||||||||||||||||||||||||||||||||||||||||||||||||||||||
Sbjct  1371942  ACTCCAGAAATTGCTGAAATTAGGGGTGTTTCAATGGGCAAAGATGTACTATCACCTCCT  1372001

Query  841      GCGCATAGTGCTTTTTCGACACCGATAGAATTTTGCTATTTCATTAAACAGCTAAGAGAT  900
                ||||||||||||||||||||||||||||||||||||||||||||||||||||||||||||
Sbjct  1372002  GCGCATAGTGCTTTTTCGACACCGATAGAATTTTGCTATTTCATTAAACAGCTAAGAGAT  1372061

Query  901      CTTTCTAATGGCAAACCAATAGGGTTTAAGCTATGTATTGGTAGTCATGTTGAGTTTTTA  960
                ||||||||||||||||||||||||||||||||||||||||||||||||||||||||||||
Sbjct  1372062  CTTTCTAATGGCAAACCAATAGGGTTTAAGCTATGTATTGGTAGTCATGTTGAGTTTTTA  1372121

Query  961      GCTATTTGTAAAGCAATGCTTGAGACAGGAATAAGACCTGATTTTATAACTGTTGATGGC  1020
                ||||||||||||||||||||||||||||||||||||||||||||||||||||||||||||
Sbjct  1372122  GCTATTTGTAAAGCAATGCTTGAGACAGGAATAAGACCTGATTTTATAACTGTTGATGGC  1372181

Query  1021     GCTGATGGTGGTACAGGGGCGGCACCACTTGAGTTCTCTAATCATATTGGTATGCCTTTA  1080
                ||||||||||||||||||||||||||||||||||||||||||||||||||||||||||||
Sbjct  1372182  GCTGATGGTGGTACAGGGGCGGCACCACTTGAGTTCTCTAATCATATTGGTATGCCTTTA  1372241

Query  1081     GAAGATAGTTTAATCTTTGTGCATAATGCTTTGGTTGGTTGTGGCTTACGTGATGAGATT  1140
                ||||||||||||||||||||||||||||||||||||||||||||||||||||||||||||
Sbjct  1372242  GAAGATAGTTTAATCTTTGTGCATAATGCTTTGGTTGGTTGTGGCTTACGTGATGAGATT  1372301

Query  1141     CGTATAATTGCAAGTAGTAAAGTAGCTACAGGTTTTGATATGGTGAGGTTATTTGCTATG  1200
                ||||||||||||||||||||||||||||||||||||||||||||||||||||||||||||
Sbjct  1372302  CGTATAATTGCAAGTAGTAAAGTAGCTACAGGTTTTGATATGGTGAGGTTATTTGCTATG  1372361

Query  1201     GGAGCAGATACTTGTAACTCAGCTAGAGCAATGATGCTTGCTATCGGCTGTATTCAGTCA  1260
                ||||||||||||||||||||||||||||||||||||||||||||||||||||||||||||
Sbjct  1372362  GGAGCAGATACTTGTAACTCAGCTAGAGCAATGATGCTTGCTATCGGCTGTATTCAGTCA  1372421

Query  1261     CGACAGTGTAATAACAACACTTGTCCAGTTGGTGTTGCTACACAAAATCCACGTCTTGAA  1320
```

```
                  ||||||||||||||||||||||||||||||||||||||||||||||||||||||||||||
Sbjct  1372422  CGACAGTGTAATAACAACACTTGTCCAGTTGGTGTTGCTACACAAAATCCACGTCTTGAA  1372481

Query  1321     AAAGCTTTGGTAGTTGAAGACAAAATGTATCGAGTTTATAATTTTCATAAAGTTACAATT  1380
                ||||||||||||||||||||||||||||||||||||||||||||||||||||||||||||
Sbjct  1372482  AAAGCTTTGGTAGTTGAAGACAAAATGTATCGAGTTTATAATTTTCATAAAGTTACAATT  1372541

Query  1381     CAATCTTGTTTAGAAATTATTGGAGCTATGGGGCTTATTTCTACTGATGATGTTGAGCCA  1440
                ||||||||||||||||||||||||||||||||||||||||||||||||||||||||||||
Sbjct  1372542  CAATCTTGTTTAGAAATTATTGGAGCTATGGGGCTTATTTCTACTGATGATGTTGAGCCA  1372601

Query  1441     GAAAACCTTAAAAAGCGTATATCTGTAAATGAGATTAAATCATATGCAGATCTGTATGAT  1500
                ||||||||||||||||||||||||||||||||||||||||||||||||||||||||||||
Sbjct  1372602  GAAAACCTTAAAAAGCGTATATCTGTAAATGAGATTAAATCATATGCAGATCTGTATGAT  1372661

Query  1501     TTTATTCCAGAGAGATGTTTAGTCGATGGTAATATCCCAGCTAGTTTTGCAAGATCTTGG  1560
                ||||||||||||||||||||||||||||||||||||||||||||||||||||||||||||
Sbjct  1372662  TTTATTCCAGAGAGATGTTTAGTCGATGGTAATATCCCAGCTAGTTTTGCAAGATCTTGG  1372721

Query  1561     GAAAAGGCTCGAGCTGATTCTTTTTAGTTTCTGAATATAAATAATAATTTTTGTCTAATC  1620
                ||||||||||||||||||||||||||||||||||||||||||||||||||||||||||||
Sbjct  1372722  GAAAAGGCTCGAGCTGATTCTTTTTAGTTTCTGAATATAAATAATAATTTTTGTCTAATC  1372781

Query  1621     AATTTGCTAAAATAGCTTCTATAAGTATTCTGATTAATCAAGCATGGCACTATACACTTA  1680
                ||||||||||||||||||||||||||||||||||||||||||||||||||||||||||||
Sbjct  1372782  AATTTGCTAAAATAGCTTCTATAAGTATTCTGATTAATCAAGCATGGCACTATACACTTA  1372841

Query  1681     CCCATCAAATAAACTTGAGTATCTTGTACAAGTTCTATCTAAACTTCTAGATGTTGaaaa  1740
                ||||||||||||||||||||||||||||||||||||||||||||||||||||||||||||
Sbjct  1372842  CCCATCAAATAAACTTGAGTATCTTGTACAAGTTCTATCTAAACTTCTAGATGTTGAAAA  1372901

Query  1741     aaaaGATTTATTTACACCAACACAGTTAATTGTTGGTAGCCGTGGTATGCAACATTGGTT  1800
                ||||||||||||||||||||||||||||||||||||||||||||||||||||||||||||
Sbjct  1372902  AAAAGATTTATTTACACCAACACAGTTAATTGTTGGTAGCCGTGGTATGCAACATTGGTT  1372961

Query  1801     AAGTATGCAACTAGCCGAATATCGCAATATTGCAATGAATCTCAAATATGACATGATAAA  1860
                ||||||||||||||||||||||||||||||||||||||||||||||||||||||||||||
Sbjct  1372962  AAGTATGCAACTAGCCGAATATCGCAATATTGCAATGAATCTCAAATATGACATGATAAA  1373021

Query  1861     TGGTTATATACTTGATATCTGCTATGAACTCACAGCAAAACAAGAATATAAAAAAGCATA  1920
                ||||||||||||||||||||||||||||||||||||||||||||||||||||||||||||
Sbjct  1373022  TGGTTATATACTTGATATCTGCTATGAACTCACAGCAAAACAAGAATATAAAAAAGCATA  1373081

Query  1921     TACAAAAGATATTTTAGCTTGGAGAGTTTTTAGGTTGCTATCTAGCCAAAATCAGGaaaa  1980
                ||||||||||||||||||||||||||||||||||||||||||||||||||||||||||||
Sbjct  1373082  TACAAAAGATATTTTAGCTTGGAGAGTTTTTAGGTTGCTATCTAGCCAAAATCAGGAAAA  1373141

Query  1981     aaaCACTCATTCCCACGCAGGTGGGAATCTCATAGCTGATGGATTAAGTTCAAAAGATAC  2040
```

```
                    ||||||||||||||||||||||||||||||||||||||||||||||||||||||||||||
Sbjct  1373142   AAACACTCATTCCCACGCAGGTGGGAATCTCATAGCTGATGGATTAAGTTCAAAAGATAC  1373201

Query  2041      TAGCCTACGCAGGTATGACAATAGAGATGGTTGGAGTAGTAAGCTTAAAGAGTACTACCA  2100
                 ||||||||||||||||||||||||||||||||||||||||||||||||||||||||||||
Sbjct  1373202   TAGCCTACGCAGGTATGACAATAGAGATGGTTGGAGTAGTAAGCTTAAAGAGTACTACCA  1373261

Query  2101      AGATAGTGATCTCAAAAAATATCAGTTGTCAGTTAAAATAGCAGAGACCTTCTCAAAATA  2160
                 ||||||||||||||||||||||||||||||||||||||||||||||||||||||||||||
Sbjct  1373262   AGATAGTGATCTCAAAAAATATCAGTTGTCAGTTAAAATAGCAGAGACCTTCTCAAAATA  1373321

Query  2161      TATATCATATCGTAGTGAGTGGCTACAAAAGTGGGAGAAGGACGAATATATAAATCCCTC  2220
                 ||||||||||||||||||||||||||||||||||||||||||||||||||||||||||||
Sbjct  1373322   TATATCATATCGTAGTGAGTGGCTACAAAAGTGGGAGAAGGACGAATATATAAATCCCTC  1373381

Query  2221      TAAACTAGAGAATGATGAAGATTGGCAGATGCTAATTTGGCAGAATCTTGTCAAAGATAT  2280
                 ||||||||||||||||||||||||||||||||||||||||||||||||||||||||||||
Sbjct  1373382   TAAACTAGAGAATGATGAAGATTGGCAGATGCTAATTTGGCAGAATCTTGTCAAAGATAT  1373441

Query  2281      TGCAGAAACACCATATAAAGTCCAATTAGAAGCACTTCAAAAGCTTGATAAACAAAACTT  2340
                 ||||||||||||||||||||||||||||||||||||||||||||||||||||||||||||
Sbjct  1373442   TGCAGAAACACCATATAAAGTCCAATTAGAAGCACTTCAAAAGCTTGATAAACAAAACTT  1373501

Query  2341      AGAAAAAATTAAATATACCTAGTGATATTTATATATTTGGAGTTAACACAATATCTCCAAA  2400
                 ||||||||||||||||||||||||||||||||||||||||||||||||||||||||||||
Sbjct  1373502   AGAAAAAATTAAATATACCTAGTGATATTTATATATTTGGAGTTAACACAATATCTCCAAA  1373561

Query  2401      AAATCTTAAATTTATATTTGAATTAGCTAAGTATATTAATGTACATATTCTATATATTAA  2460
                 ||||||||||||||||||||||||||||||||||||||||||||||||||||||||||||
Sbjct  1373562   AAATCTTAAATTTATATTTGAATTAGCTAAGTATATTAATGTACATATTCTATATATTAA  1373621

Query  2461      TCCATGTAGTGAGTATTGGTATGATTTGCATAAAAGTAAAATATCAGCTTGGTTAGATAG  2520
                 ||||||||||||||||||||||||||||||||||||||||||||||||||||||||||||
Sbjct  1373622   TCCATGTAGTGAGTATTGGTATGATTTGCATAAAAGTAAAATATCAGCTTGGTTAGATAG  1373681

Query  2521      TGATGACTATGAAATTCAACCACTTTTGGCAAATCTTGGACAGCAGGGTAAAGAATTTTT  2580
                 ||||||||||||||||||||||||||||||||||||||||||||||||||||||||||||
Sbjct  1373682   TGATGACTATGAAATTCAACCACTTTTGGCAAATCTTGGACAGCAGGGTAAAGAATTTTT  1373741

Query  2581      TAATCAGCTTCTAGAAAATGAGCAACAGCAAGAATTAGAAGTATTCGAAAAGTTTGATAA  2640
                 ||||||||||||||||||||||||||||||||||||||||||||||||||||||||||||
Sbjct  1373742   TAATCAGCTTCTAGAAAATGAGCAACAGCAAGAATTAGAAGTATTCGAAAAGTTTGATAA  1373801

Query  2641      AGAATCATTAGCTTTTGAGAAACTTACCGATAATAATCAAACACAGCTAGTAAGCCTACA  2700
                 ||||||||||||||||||||||||||||||||||||||||||||||||||||||||||||
Sbjct  1373802   AGAATCATTAGCTTTTGAGAAACTTACCGATAATAATCAAACACAGCTAGTAAGCCTACA  1373861

Query  2701      GCGAAACTTACTTGAACTAGATTGCCAAAACCATGCCAAGCAAAAAGATTTAAGTATTAG  2760
```

```
                  ||||||||||||||||||||||||||||||||||||||||||||||||||||||||||||
Sbjct  1373862   GCGAAACTTACTTGAACTAGATTGCCAAAACCATGCCAAGCAAAAAGATTTAAGTATTAG  1373921

Query  2761      TATTAACTCTTGTCATAGTCCACTTAGAGAGGTGCAGATTTTGCATGATAAGCTTTTGGA  2820
                  ||||||||||||||||||||||||||||||||||||||||||||||||||||||||||||
Sbjct  1373922   TATTAACTCTTGTCATAGTCCACTTAGAGAGGTGCAGATTTTGCATGATAAGCTTTTGGA  1373981

Query  2821      TATGATCAAAGCAGATCCAAATATCAAGCCTAGAGATATCCTAGTGATGTGCCCAAATAT  2880
                  ||||||||||||||||||||||||||||||||||||||||||||||||||||||||||||
Sbjct  1373982   TATGATCAAAGCAGATCCAAATATCAAGCCTAGAGATATCCTAGTGATGTGCCCAAATAT  1374041

Query  2881      TGAAGATTATTCACCATATATTGATAGTGTTTTCTCAAGATATCCAACTGATAAAAAACT  2940
                  ||||||||||||||||||||||||||||||||||||||||||||||||||||||||||||
Sbjct  1374042   TGAAGATTATTCACCATATATTGATAGTGTTTTCTCAAGATATCCAACTGATAAAAAACT  1374101

Query  2941      GCCATGCTCAATCGCAGATAGAACGCTACTTGATTCAGAGCCTTTAGCTGCTAGTTTTAT  3000
                  ||||||||||||||||||||||||||||||||||||||||||||||||||||||||||||
Sbjct  1374102   GCCATGCTCAATCGCAGATAGAACGCTACTTGATTCAGAGCCTTTAGCTGCTAGTTTTAT  1374161

Query  3001      TGAGCTTTTGCAGTTGCCTGAGAGTAATTTTGAGGTTAATAAAATACTTGATTATCTAGC  3060
                  ||||||||||||||||||||||||||||||||||||||||||||||||||||||||||||
Sbjct  1374162   TGAGCTTTTGCAGTTGCCTGAGAGTAATTTTGAGGTTAATAAAATACTTGATTATCTAGC  1374221

Query  3061      TGTACCGGCTATCCAACAGAAGTTTAAGATCACAGATGAACAATTAGAGGCTATTCGCTA  3120
                  ||||||||||||||||||||||||||||||||||||||||||||||||||||||||||||
Sbjct  1374222   TGTACCGGCTATCCAACAGAAGTTTAAGATCACAGATGAACAATTAGAGGCTATTCGCTA  1374281

Query  3121      TTGGTTAAAAGAGTCATGTATTCATCATAGCAATAATGATCAGACATTCTCATGGAGCTG  3180
                  ||||||||||||||||||||||||||||||||||||||||||||||||||||||||||||
Sbjct  1374282   TTGGTTAAAAGAGTCATGTATTCATCATAGCAATAATGATCAGACATTCTCATGGAGCTG  1374341

Query  3181      GGGACTAAGAAGATTAATGCTTGGGTTTAGCTATAGTGATAGTAGTTACATCATTGATGA  3240
                  ||||||||||||||||||||||||||||||||||||||||||||||||||||||||||||
Sbjct  1374342   GGGACTAAGAAGATTAATGCTTGGGTTTAGCTATAGTGATAGTAGTTACATCATTGATGA  1374401

Query  3241      TAAATTAATGACGGTACCAGTTATTGAAGGAAGTGAGATCGCTGAACTTGGTGGTTTGTA  3300
                  ||||||||||||||||||||||||||||||||||||||||||||||||||||||||||||
Sbjct  1374402   TAAATTAATGACGGTACCAGTTATTGAAGGAAGTGAGATCGCTGAACTTGGTGGTTTGTA  1374461

Query  3301      TGAGTTATTAGAGCTACTTGAAAGATATTCTCAAAATTTGCTTAAACCAAGAAGTCTTGC  3360
                  ||||||||||||||||||||||||||||||||||||||||||||||||||||||||||||
Sbjct  1374462   TGAGTTATTAGAGCTACTTGAAAGATATTCTCAAAATTTGCTTAAACCAAGAAGTCTTGC  1374521

Query  3361      AAATTGGCAAGTTTATCTTTTAGAAATGTTTGATGATGTCTTTGATGTCACTAATGATGA  3420
                  ||||||||||||||||||||||||||||||||||||||||||||||||||||||||||||
Sbjct  1374522   AAATTGGCAAGTTTATCTTTTAGAAATGTTTGATGATGTCTTTGATGTCACTAATGATGA  1374581

Query  3421      GCAATATATTGCTaaaaaaaTCAAAGATATAATCGCAAAAACAGTCACTACCGCTAAAAA  3480
```

```
                  ||||||||||||||||||||||||||||||||||||||||||||||||||||||||||||
Sbjct  1374582  GCAATATATTGCTAAAAAAATCAAAGATATAATCGCAAAAACAGTCACTACCGCTAAAAA  1374641

Query  3481     TATATTGTTAGATCAAGAAATTGATTTATATACAATTAGATATTGTTTAATTTCTCAATT  3540
                  ||||||||||||||||||||||||||||||||||||||||||||||||||||||||||||
Sbjct  1374642  TATATTGTTAGATCAAGAAATTGATTTATATACAATTAGATATTGTTTAATTTCTCAATT  1374701

Query  3541     ATCAGAGCCAATTATTAATAACCATTTTTTAAATGGTAAGGTGACTTTTTGTTCGATGAC  3600
                  ||||||||||||||||||||||||||||||||||||||||||||||||||||||||||||
Sbjct  1374702  ATCAGAGCCAATTATTAATAACCATTTTTTAAATGGTAAGGTGACTTTTTGTTCGATGAC  1374761

Query  3601     GCCGATGCGTAGTGTACCATTTAGAGTTATAGCTATGCTTGGATTAAATAATGGTAAGTT  3660
                  ||||||||||||||||||||||||||||||||||||||||||||||||||||||||||||
Sbjct  1374762  GCCGATGCGTAGTGTACCATTTAGAGTTATAGCTATGCTTGGATTAAATAATGGTAAGTT  1374821

Query  3661     CCCACACCAAGAATCTGCAATTAGCTTTGATCTGATAGCAAGGCTTGGTAGaaaaaaaGG  3720
                  ||||||||||||||||||||||||||||||||||||||||||||||||||||||||||||
Sbjct  1374822  CCCACACCAAGAATCTGCAATTAGCTTTGATCTGATAGCAAGGCTTGGTAGAAAAAAAGG  1374881

Query  3721     TGATAGAACCAAACGCGATGATGATAGATATTTGTTTTTAGAGGCTATTTTATCTGCTAG  3780
                  ||||||||||||||||||||||||||||||||||||||||||||||||||||||||||||
Sbjct  1374882  TGATAGAACCAAACGCGATGATGATAGATATTTGTTTTTAGAGGCTATTTTATCTGCTAG  1374941

Query  3781     AGATTATTTGTATATAAGCTATATAGGTAGAAGTGTTAAGACTAATGTTGAGCAACAGCC  3840
                  ||||||||||||||||||||||||||||||||||||||||||||||||||||||||||||
Sbjct  1374942  AGATTATTTGTATATAAGCTATATAGGTAGAAGTGTTAAGACTAATGTTGAGCAACAGCC  1375001

Query  3841     AAGTTTGATACTTAAAGAGTTGACTAGCTATTTGAATTTGAACTACGATTGGGGAAAGGA  3900
                  ||||||||||||||||||||||||||||||||||||||||||||||||||||||||||||
Sbjct  1375002  AAGTTTGATACTTAAAGAGTTGACTAGCTATTTGAATTTGAACTACGATTGGGGAAAGGA  1375061

Query  3901     GGATATAAAAGAGTATCCATTGCATGCTTTTAGTTCTAAATGTTATTCTGACAGTTATAG  3960
                  ||||||||||||||||||||||||||||||||||||||||||||||||||||||||||||
Sbjct  1375062  GGATATAAAAGAGTATCCATTGCATGCTTTTAGTTCTAAATGTTATTCTGACAGTTATAG  1375121

Query  3961     AAGCTATGATAAAGCTTGGTTGAAGTTGTTACAGTCTGAGCCAAGGGGCTTTTATGATTC  4020
                  ||||||||||||||||||||||||||||||||||||||||||||||||||||||||||||
Sbjct  1375122  AAGCTATGATAAAGCTTGGTTGAAGTTGTTACAGTCTGAGCCAAGGGGCTTTTATGATTC  1375181

Query  4021     GGCTTCATTAAGTAACTGTGCTAATTTACCCAAAAATCTAAGTATTTCAAAACTTGTTAA  4080
                  ||||||||||||||||||||||||||||||||||||||||||||||||||||||||||||
Sbjct  1375182  GGCTTCATTAAGTAACTGTGCTAATTTACCCAAAAATCTAAGTATTTCAAAACTTGTTAA  1375241

Query  4081     AGTTTTTGATGACCCTATTAAGGCTTATGCAAATTATACTTTAGAGCTGTATTTAGAAGA  4140
                  ||||||||||||||||||||||||||||||||||||||||||||||||||||||||||||
Sbjct  1375242  AGTTTTTGATGACCCTATTAAGGCTTATGCAAATTATACTTTAGAGCTGTATTTAGAAGA  1375301

Query  4141     TGACTTTGAGGAGCTAGAAGATAGTGAACCATTTGATATCAATAGTTTGGATAAGCATAA  4200
```

```
                 ||||||||||||||||||||||||||||||||||||||||||||||||||||||||||||
Sbjct  1375302   TGACTTTGAGGAGCTAGAAGATAGTGAACCATTTGATATCAATAGTTTGGATAAGCATAA  1375361

Query  4201      GCTCAAACAAGCTTTGTTAAAAACTTTTGAAGATGAAAAAGACAAAGATTTAACAATAAA  4260
                 ||||||||||||||||||||||||||||||||||||||||||||||||||||||||||||
Sbjct  1375362   GCTCAAACAAGCTTTGTTAAAAACTTTTGAAGATGAAAAAGACAAAGATTTAACAATAAA  1375421

Query  4261      AACAGCCAAACTAAGTGGCAAGCTTCCGGAGTCAGTACTTACTGATACTGAGATTAAAGA  4320
                 ||||||||||||||||||||||||||||||||||||||||||||||||||||||||||||
Sbjct  1375422   AACAGCCAAACTAAGTGGCAAGCTTCCGGAGTCAGTACTTACTGATACTGAGATTAAAGA  1375481

Query  4321      GGAAGTTGAGAATATTCAAAAGCTACTAGACAAAATGAGCTTAGCAAGTTACGAGTCAAA  4380
                 ||||||||||||||||||||||||||||||||||||||||||||||||||||||||||||
Sbjct  1375482   GGAAGTTGAGAATATTCAAAAGCTACTAGACAAAATGAGCTTAGCAAGTTACGAGTCAAA  1375541

Query  4381      ATATTTTCATCAAGAGATATTAGGGTATGAGTTAGAAGCTAATTGTTATATTAAAAATAA  4440
                 ||||||||||||||||||||||||||||||||||||||||||||||||||||||||||||
Sbjct  1375542   ATATTTTCATCAAGAGATATTAGGGTATGAGTTAGAAGCTAATTGTTATATTAAAAATAA  1375601

Query  4441      TCAAATTATGTTATCTACACCATCTAGTTTGAATATAGGaaaaaaaTTTGAATTGTACTT  4500
                 ||||||||||||||||||||||||||||||||||||||||||||||||||||||||||||
Sbjct  1375602   TCAAATTATGTTATCTACACCATCTAGTTTGAATATAGGAAAAAAATTTGAATTGTACTT  1375661

Query  4501      AATAGCTTTATTAGTAGCATATTCTGAACAAAGAGATATTAGCGCAGTCTATTATGGTAT  4560
                 ||||||||||||||||||||||||||||||||||||||||||||||||||||||||||||
Sbjct  1375662   AATAGCTTTATTAGTAGCATATTCTGAACAAAGAGATATTAGCGCAGTCTATTATGGTAT  1375721

Query  4561      AGaaaaaaaTCAAGTAGAAGAATTTAGAGTAGAAAATATTGAATATACAACAGCTAAAGG  4620
                 ||||||||||||||||||||||||||||||||||||||||||||||||||||||||||||
Sbjct  1375722   AGAAAAAAATCAAGTAGAAGAATTTAGAGTAGAAAATATTGAATATACAACAGCTAAAGG  1375781

Query  4621      AATATTAGAGCATTATATAAATCAAGCTGAGCAAATAGTAGTTAAGCCTCAACTAGCACA  4680
                 ||||||||||||||||||||||||||||||||||||||||||||||||||||||||||||
Sbjct  1375782   AATATTAGAGCATTATATAAATCAAGCTGAGCAAATAGTAGTTAAGCCTCAACTAGCACA  1375841

Query  4681      TTTATCTTTAGCTGAAGCTATTTGTGATTATAAAAATAATACTGATAAGAAGAAGCAACA  4740
                 ||||||||||||||||||||||||||||||||||||||||||||||||||||||||||||
Sbjct  1375842   TTTATCTTTAGCTGAAGCTATTTGTGATTATAAAAATAATACTGATAAGAAGAAGCAACA  1375901

Query  4741      GGCTTGGCAAAAAGTAATAAAATCATCACAACATAATTTCAAAGCTCTAGAAGATAATAG  4800
                 ||||||||||||||||||||||||||||||||||||||||||||||||||||||||||||
Sbjct  1375902   GGCTTGGCAAAAAGTAATAAAATCATCACAACATAATTTCAAAGCTCTAGAAGATAATAG  1375961

Query  4801      TTATTTCAAGCTATTCTATAATAAGTTTCCAACTGTAGATGATTTTGAGGGTGAAAAAAT  4860
                 ||||||||||||||||||||||||||||||||||||||||||||||||||||||||||||
Sbjct  1375962   TTATTTCAAGCTATTCTATAATAAGTTTCCAACTGTAGATGATTTTGAGGGTGAAAAAAT  1376021

Query  4861      TTACAAAAAGTTCTTTGAGGTTATTTATGAAAGTCAATCATGAGCATTCTAATATCCCAC  4920
```

```
                      ||||||||||||||||||||||||||||||||||||||||||||||||||||||||||||
Sbjct  1376022  TTACAAAAAGTTCTTTGAGGTTATTTATGAAAGTCAATCATGAGCATTCTAATATCCCAC  1376081

Query  4921     AGCTATACAAAAATATTTATATTAGCTTTGAGATTGCTCTAGCTGCTTGTATATGTTTTC  4980
                ||||||||||||||||||||||||||||||||||||||||||||||||||||||||||||
Sbjct  1376082  AGCTATACAAAAATATTTATATTAGCTTTGAGATTGCTCTAGCTGCTTGTATATGTTTTC  1376141

Query  4981     TGTTAGGGTTTTATATGTCGAATTTGTTACATCGTGGTCAGTCGATTATTGGAGGCTTTT  5040
                ||||||||||||||||||||||||||||||||||||||||||||||||||||||||||||
Sbjct  1376142  TGTTAGGGTTTTATATGTCGAATTTGTTACATCGTGGTCAGTCGATTATTGGAGGCTTTT  1376201

Query  5041     GGTGTTTGATTACAGTTTCAACAATCTTACAGCTAAGTATTAGAGATTCGTATTTGGCTG  5100
                ||||||||||||||||||||||||||||||||||||||||||||||||||||||||||||
Sbjct  1376202  GGTGTTTGATTACAGTTTCAACAATCTTACAGCTAAGTATTAGAGATTCGTATTTGGCTG  1376261

Query  5101     CTTTTCAAATATTGGTTGGCTCTGTGATAGGAGGGATTACGGCATTTATTTTTACATCAG  5160
                ||||||||||||||||||||||||||||||||||||||||||||||||||||||||||||
Sbjct  1376262  CTTTTCAAATATTGGTTGGCTCTGTGATAGGAGGGATTACGGCATTTATTTTTACATCAG  1376321

Query  5161     TTTTAGGCTATTACTACTATGTTATGATTTTGGCTGTAGCTATTAGTGTTTTTATTACTG  5220
                ||||||||||||||||||||||||||||||||||||||||||||||||||||||||||||
Sbjct  1376322  TTTTAGGCTATTACTACTATGTTATGATTTTGGCTGTAGCTATTAGTGTTTTTATTACTG  1376381

Query  5221     CAAGTATAGGGTTAGAAAATGCGGTTAAAATGAGTAGCGCAAATGCTGGAGTAATTGTCG  5280
                ||||||||||||||||||||||||||||||||||||||||||||||||||||||||||||
Sbjct  1376382  CAAGTATAGGGTTAGAAAATGCGGTTAAAATGAGTAGCGCAAATGCTGGAGTAATTGTCG  1376441

Query  5281     CATTAGGTCTGTATCAGCCTAGTTATTCACCTTTTCTAAATACAGGTTTACGCTTGATAG  5340
                ||||||||||||||||||||||||||||||||||||||||||||||||||||||||||||
Sbjct  1376442  CATTAGGTCTGTATCAGCCTAGTTATTCACCTTTTCTAAATACAGGTTTACGCTTGATAG  1376501

Query  5341     AGACTTTTAGTGGTACTGCAATAGCtttatttttttatatttattagtaggatttttaGGA  5400
                ||||||||||||||||||||||||||||||||||||||||||||||||||||||||||||
Sbjct  1376502  AGACTTTTAGTGGTACTGCAATAGCTTTATTTTTTTATATTTATTAGTAGGATTTTTAGGA  1376561

Query  5401     TAAGAACAGACGACTAATATATAGATTTATAGGATTAAATGAGTAATAATGAAAAACCTA  5460
                ||||||||||||||||||||||||||||||||||||||||||||||||||||||||||||
Sbjct  1376562  TAAGAACAGACGACTAATATATAGATTTATAGGATTAAATGAGTAATAATGAAAAACCTA  1376621

Query  5461     AACGCAAAAAGAATAACCCTGCAAGGTCGCCATATTATTAAGGCAAGTGTTAAGCTCGAT  5520
                ||||||||||||||||||||||||||||||||||||||||||||||||||||||||||||
Sbjct  1376622  AACGCAAAAAGAATAACCCTGCAAGGTCGCCATATTATTAAGGCAAGTGTTAAGCTCGAT  1376681

Query  5521     GCAATACAGTATGACAGAATCTAAAAAGATGAGAAACATATGAACATACTACAGCGAATA  5580
                ||||||||||||||||||||||||||||||||||||||||||||||||||||||||||||
Sbjct  1376682  GCAATACAGTATGACAGAATCTAAAAAGATGAGAAACATATGAACATACTACAGCGAATA  1376741

Query  5581     CAAAATGGTGAAGATAGTTACACGCAATTTAAACAAGATATAAATAATGCTGATAGTCTT  5640
```

```
                 ||||||||||||||||||||||||||||||||||||||||||||||||||||||||||||
Sbjct  1376742  CAAAATGGTGAAGATAGTTACACGCAATTTAAACAAGATATAAATAATGCTGATAGTCTT  1376801

Query  5641     GCACAAGAGCTTGTTGCTTTTTCAAATGCTAATGGCGGTATTTTAATCATTGGTGTTGCT  5700
                ||||||||||||||||||||||||||||||||||||||||||||||||||||||||||||
Sbjct  1376802  GCACAAGAGCTTGTTGCTTTTTCAAATGCTAATGGCGGTATTTTAATCATTGGTGTTGCT  1376861

Query  5701     GATGATGGTAAAATTATCGGATTATCAAAAGATGATATTCATAGGATAAATCAGCTTATA  5760
                ||||||||||||||||||||||||||||||||||||||||||||||||||||||||||||
Sbjct  1376862  GATGATGGTAAAATTATCGGATTATCAAAAGATGATATTCATAGGATAAATCAGCTTATA  1376921

Query  5761     AGTAATGTTTGCAGTACAAATATAAAACCACCAATAAGCGTACTAACAAAGATAGAGAAT  5820
                ||||||||||||||||||||||||||||||||||||||||||||||||||||||||||||
Sbjct  1376922  AGTAATGTTTGCAGTACAAATATAAAACCACCAATAAGCGTACTAACAAAGATAGAGAAT  1376981

Query  5821     ATAGATGATAAAGAAATACTTATAATAGAAATAAACAATGGAGTAAATAAACCATATAGT  5880
                ||||||||||||||||||||||||||||||||||||||||||||||||||||||||||||
Sbjct  1376982  ATAGATGATAAAGAAATACTTATAATAGAAATAAACAATGGAGTAAATAAACCATATAGT  1377041

Query  5881     ACTAACAAAGGAGTATATTTGACAAAAGTTGGTAGTGATAAAAGAAAAATGTCTCAAGAA  5940
                ||||||||||||||||||||||||||||||||||||||||||||||||||||||||||||
Sbjct  1377042  ACTAACAAAGGAGTATATTTGACAAAAGTTGGTAGTGATAAAAGAAAAATGTCTCAAGAA  1377101

Query  5941     GAGCTAGGTAGACTTTTTAGACAGGCTCCAAAACTGTATCCTGATGAAGAAGTGCTATAT  6000
                ||||||||||||||||||||||||||||||||||||||||||||||||||||||||||||
Sbjct  1377102  GAGCTAGGTAGACTTTTTAGACAGGCTCCAAAACTGTATCCTGATGAAGAAGTGCTATAT  1377161

Query  6001     AAAACATCGCTAGAAAACTTTGATGTTTCAAAGTTTAGAAAGTTTCTAAAAACAGATAAC  6060
                ||||||||||||||||||||||||||||||||||||||||||||||||||||||||||||
Sbjct  1377162  AAAACATCGCTAGAAAACTTTGATGTTTCAAAGTTTAGAAAGTTTCTAAAAACAGATAAC  1377221

Query  6061     TATACTACATTTTCAAAACTAGAGAATGGTAGTCTAGATTTATCAACTATTCTAGAAAAT  6120
                ||||||||||||||||||||||||||||||||||||||||||||||||||||||||||||
Sbjct  1377222  TATACTACATTTTCAAAACTAGAGAATGGTAGTCTAGATTTATCAACTATTCTAGAAAAT  1377281

Query  6121     AAAGATTTAGCAAAAGATGGCAAACTAACTTTAGCCGGTAATCTTATATTTGGAGAAGAG  6180
                ||||||||||||||||||||||||||||||||||||||||||||||||||||||||||||
Sbjct  1377282  AAAGATTTAGCAAAAGATGGCAAACTAACTTTAGCCGGTAATCTTATATTTGGAGAAGAG  1377341

Query  6181     CCACAGCGGTTTTGTCCAAGCTTTTATATAGACTGCTGTTATTTTGATGGTAATGATATT  6240
                ||||||||||||||||||||||||||||||||||||||||||||||||||||||||||||
Sbjct  1377342  CCACAGCGGTTTTGTCCAAGCTTTTATATAGACTGCTGTTATTTTGATGGTAATGATATT  1377401

Query  6241     TCTGTATCGAAATTTATATCTAAAAAAGTTATCAAAGGAACATTTTCTGAACTATATGAA  6300
                ||||||||||||||||||||||||||||||||||||||||||||||||||||||||||||
Sbjct  1377402  TCTGTATCGAAATTTATATCTAAAAAAGTTATCAAAGGAACATTTTCTGAACTATATGAA  1377461

Query  6301     GATACCATGAGATTTTTGCTTAGTCAGTTAAAAAGTTATCAAATAGATTTAGATTTTAAC  6360
```

```
                   ||||||||||||||||||||||||||||||||||||||||||||||||||||||||||||
Sbjct  1377462   GATACCATGAGATTTTTGCTTAGTCAGTTAAAAAGTTATCAAATAGATTTAGATTTTAAC  1377521

Query  6361      TCAAACCCACAATTAGAGGTAAATGAATCTGCACTAACAGAGATAGTTGTAAATGCACTT  6420
                 ||||||||||||||||||||||||||||||||||||||||||||||||||||||||||||
Sbjct  1377522   TCAAACCCACAATTAGAGGTAAATGAATCTGCACTAACAGAGATAGTTGTAAATGCACTT  1377581

Query  6421      GTGCATAGAGACTATTATATAAACTCTTCTATAAAGGTTTTTATATTTCATAACCGTGTT  6480
                 ||||||||||||||||||||||||||||||||||||||||||||||||||||||||||||
Sbjct  1377582   GTGCATAGAGACTATTATATAAACTCTTCTATAAAGGTTTTTATATTTCATAACCGTGTT  1377641

Query  6481      GAGATTATTAGCCCTGGTAAATTACCTGACACTTTAACAGTAGATAAGATTAAAAATGGC  6540
                 ||||||||||||||||||||||||||||||||||||||||||||||||||||||||||||
Sbjct  1377642   GAGATTATTAGCCCTGGTAAATTACCTGACACTTTAACAGTAGATAAGATTAAAAATGGC  1377701

Query  6541      ATAGCAATTCAGCGAAACCCTATCTTAGCCTCAATATGCAAAACATTATTACCTTATACA  6600
                 ||||||||||||||||||||||||||||||||||||||||||||||||||||||||||||
Sbjct  1377702   ATAGCAATTCAGCGAAACCCTATCTTAGCCTCAATATGCAAAACATTATTACCTTATACA  1377761

Query  6601      GGTTATGGTAGTGGAATCAAAAGAGCTATTGAGCTAATTCCTAATATTGAATTTATAAAT  6660
                 ||||||||||||||||||||||||||||||||||||||||||||||||||||||||||||
Sbjct  1377762   GGTTATGGTAGTGGAATCAAAAGAGCTATTGAGCTAATTCCTAATATTGAATTTATAAAT  1377821

Query  6661      GATATAGATAGTGAACAGTTTAGATGTATTATaaaaagagatgaaagttcaccaaaagag  6720
                 ||||||||||||||||||||||||||||||||||||||||||||||||||||||||||||
Sbjct  1377822   GATATAGATAGTGAACAGTTTAGATGTATTATAAAAAGAGATGAAAGTTCACCAAAAGAG  1377881

Query  6721      caagaaagttcaccaaaagagcaagaaagttcaccaaaagagcaagaaagttcaccaaTA  6780
                 ||||||||||||||||||||||||||||||||||||||||||||||||||||||||||||
Sbjct  1377882   CAAGAAAGTTCACCAAAAGAGCAAGAAAGTTCACCAAAAGAGCAAGAAAGTTCACCAATA  1377941

Query  6781      AATATAAGTTATGGTGTTGGCAGTTCACCAAAAACAGATAAGCAGATTTTAGAAATGTTT  6840
                 ||||||||||||||||||||||||||||||||||||||||||||||||||||||||||||
Sbjct  1377942   AATATAAGTTATGGTGTTGGCAGTTCACCAAAAACAGATAAGCAGATTTTAGAAATGTTT  1378001

Query  6841      AGAGAAAACCCAAAAATCACAATCAAGGAAATAGCAGAGAGATTGTCTATCACAGATAGA  6900
                 ||||||||||||||||||||||||||||||||||||||||||||||||||||||||||||
Sbjct  1378002   AGAGAAAACCCAAAAATCACAATCAAGGAAATAGCAGAGAGATTGTCTATCACAGATAGA  1378061

Query  6901      GCTGTCAAAAAGCATATAGAAAAACTAAAATACCAAGGATTAATTGCTAGAGAAGGAAGC  6960
                 ||||||||||||||||||||||||||||||||||||||||||||||||||||||||||||
Sbjct  1378062   GCTGTCAAAAAGCATATAGAAAAACTAAAATACCAAGGATTAATTGCTAGAGAAGGAAGC  1378121

Query  6961      CCTCGTAACGGCTATTGGAGAGTTATATAAATTGCAATGATGAAAGTTTTAAATAGACTT  7020
                 ||||||||||||||||||||||||||||||||||||||||||||||||||||||||||||
Sbjct  1378122   CCTCGTAACGGCTATTGGAGAGTTATATAAATTGCAATGATGAAAGTTTTAAATAGACTT  1378181

Query  7021      TGAACTATCTAGATTTTATGGGTATATAGAAGACACAGATTATGGACAACCTAAACGCAA  7080
```

```
              ||||||||||||||||||||||||||||||||||||||||||||||||||||||||||||
Sbjct  1378182  TGAACTATCTAGATTTTATGGGTATATAGAAGACACAGATTATGGACAACCTAAACGCAA  1378241

Query  7081     ACATAATAACTCTACAAGGTCGCCATATTATCGAGGCAAGTGCTGGTACTGGTAAGACTT  7140
                ||||||||||||||||||||||||||||||||||||||||||||||||||||||||||||
Sbjct  1378242  ACATAATAACTCTACAAGGTCGCCATATTATCGAGGCAAGTGCTGGTACTGGTAAGACTT  1378301

Query  7141     TTAATATTACTAAGTTGTATATCCGACTTTTGCTAGaaaaaaaaTTATTGCCAAGTAATA  7200
                ||||||||||||||||||||||||||||||||||||||||||||||||||||||||||||
Sbjct  1378302  TTAATATTACTAAGTTGTATATCCGACTTTTGCTAGAAAAAAAATTATTGCCAAGTAATA  1378361

Query  7201     TCCTTGTAATGACCTTTACCAAAGATGCAACCCAAGAGATTATTGGTAGAGTTGAAAAAG  7260
                ||||||||||||||||||||||||||||||||||||||||||||||||||||||||||||
Sbjct  1378362  TCCTTGTAATGACCTTTACCAAAGATGCAACCCAAGAGATTATTGGTAGAGTTGAAAAAG  1378421

Query  7261     AGATTCGAGATGTATTAGCAAGTTATACAGATGAGAAAAAGGAGTCTGACAAAGAAAACT  7320
                ||||||||||||||||||||||||||||||||||||||||||||||||||||||||||||
Sbjct  1378422  AGATTCGAGATGTATTAGCAAGTTATACAGATGAGAAAAAGGAGTCTGACAAAGAAAACT  1378481

Query  7321     ACAAACATCTAAAAAGATCATTACTTGAGATAGATGAGGCAGCTATATTTACTATCCATG  7380
                ||||||||||||||||||||||||||||||||||||||||||||||||||||||||||||
Sbjct  1378482  ACAAACATCTAAAAAGATCATTACTTGAGATAGATGAGGCAGCTATATTTACTATCCATG  1378541

Query  7381     GTTTTTGTAAAAAAGTCTTAAGTGAGCAAGCCTTTGCTAGCGGTATCGAGATGGATGTCT  7440
                ||||||||||||||||||||||||||||||||||||||||||||||||||||||||||||
Sbjct  1378542  GTTTTTGTAAAAAAGTCTTAAGTGAGCAAGCCTTTGCTAGCGGTATCGAGATGGATGTCT  1378601

Query  7441     CTATGGAGGTTGATACTTCAGATATTTTTCAAAAGGTTGTAGAGGATTTTTTCAGAAAAC  7500
                ||||||||||||||||||||||||||||||||||||||||||||||||||||||||||||
Sbjct  1378602  CTATGGAGGTTGATACTTCAGATATTTTTCAAAAGGTTGTAGAGGATTTTTTCAGAAAAC  1378661

Query  7501     ATATCAATAAAAGCGAAACTAATTTTGGATATTTACAAATATATAAATTACATACTCCAG  7560
                ||||||||||||||||||||||||||||||||||||||||||||||||||||||||||||
Sbjct  1378662  ATATCAATAAAAGCGAAACTAATTTTGGATATTTACAAATATATAAATTACATACTCCAG  1378721

Query  7561     AGAAATTTTTAGATGACCTTGAGAATATTATTAGGTCTAATTATGAGATTTTAACAAAGC  7620
                ||||||||||||||||||||||||||||||||||||||||||||||||||||||||||||
Sbjct  1378722  AGAAATTTTTAGATGACCTTGAGAATATTATTAGGTCTAATTATGAGATTTTAACAAAGC  1378781

Query  7621     AAGCTATAAGCTTAGACGAATTTAAGATACTCAAAAAACAACAGTTAGAATTATTTATTA  7680
                ||||||||||||||||||||||||||||||||||||||||||||||||||||||||||||
Sbjct  1378782  AAGCTATAAGCTTAGACGAATTTAAGATACTCAAAAAACAACAGTTAGAATTATTTATTA  1378841

Query  7681     ATAATCACGATATCGTAGATGATTTCTTATATAAATTAGGAAAAAGTGAGCCACAGGGCA  7740
                ||||||||||||||||||||||||||||||||||||||||||||||||||||||||||||
Sbjct  1378842  ATAATCACGATATCGTAGATGATTTCTTATATAAATTAGGAAAAAGTGAGCCACAGGGCA  1378901

Query  7741     AGAGAGTAGATGAGTATCATAGGGTTTTAGAATGGTTGAAATTAGATAATCAAACACTAT  7800
```

```
                  ||||||||||||||||||||||||||||||||||||||||||||||||||||||||||||
Sbjct  1378902   AGAGAGTAGATGAGTATCATAGGGTTTTAGAATGGTTGAAATTAGATAATCAAACACTAT  1378961

Query  7801      TCCCAGAGAATATTTCTATAATTACAGATGGCAGAAAGATAAGTGCAAAACTAATAAAAC  7860
                  ||||||||||||||||||||||||||||||||||||||||||||||||||||||||||||
Sbjct  1378962   TCCCAGAGAATATTTCTATAATTACAGATGGCAGAAAGATAAGTGCAAAACTAATAAAAC  1379021

Query  7861      CAATTTTTATTGGCGTAAAAGAATTAAGAGACCTGCAACAAGAAATAAAACAAGCACAAG  7920
                  ||||||||||||||||||||||||||||||||||||||||||||||||||||||||||||
Sbjct  1379022   CAATTTTTATTGGCGTAAAAGAATTAAGAGACCTGCAACAAGAAATAAAACAAGCACAAG  1379081

Query  7921      CAGCACAATTTATCAGAAAAGCATGCTTGCAAATCCGCCAAGATTTTGCAAAGGCAAAAG  7980
                  ||||||||||||||||||||||||||||||||||||||||||||||||||||||||||||
Sbjct  1379082   CAGCACAATTTATCAGAAAAGCATGCTTGCAAATCCGCCAAGATTTTGCAAAGGCAAAAG  1379141

Query  7981      ATCAAAAGGGTGTTTTAGACTTTGATGATTTGATTACCAAGCTTTGCCAAAGTGTCAAAA  8040
                  ||||||||||||||||||||||||||||||||||||||||||||||||||||||||||||
Sbjct  1379142   ATCAAAAGGGTGTTTTAGACTTTGATGATTTGATTACCAAGCTTTGCCAAAGTGTCAAAA  1379201

Query  8041      AATCGCCAGAGTTAGTCAAAACTTTGCAAAAACAATATCCAGTTGCTCTTATAGATGAGT  8100
                  ||||||||||||||||||||||||||||||||||||||||||||||||||||||||||||
Sbjct  1379202   AATCGCCAGAGTTAGTCAAAACTTTGCAAAAACAATATCCAGTTGCTCTTATAGATGAGT  1379261

Query  8101      TTCAAGATACTGATGCTGAGCAGTATGAGATATTAGATACTATTTATCCTTTGAAGAATA  8160
                  ||||||||||||||||||||||||||||||||||||||||||||||||||||||||||||
Sbjct  1379262   TTCAAGATACTGATGCTGAGCAGTATGAGATATTAGATACTATTTATCCTTTGAAGAATA  1379321

Query  8161      GTAGTAGTCATTCCCACAAAGGTAAGAATCTCCTTACCAATGGATTAAATTTAAAAGATA  8220
                  ||||||||||||||||||||||||||||||||||||||||||||||||||||||||||||
Sbjct  1379322   GTAGTAGTCATTCCCACAAAGGTAAGAATCTCCTTACCAATGGATTAAATTTAAAAGATA  1379381

Query  8221      CCCGCTTACGCGAGTATGACAGTAGTTCTGATCAGAACGATAGCAATCTTCTGCTCTTAA  8280
                  ||||||||||||||||||||||||||||||||||||||||||||||||||||||||||||
Sbjct  1379382   CCCGCTTACGCGAGTATGACAGTAGTTCTGATCAGAACGATAGCAATCTTCTGCTCTTAA  1379441

Query  8281      TGATAGGCGATCCAAAACAGGCAATTTATGGCTTTAGAGGTGGAGATATCTTCACATATC  8340
                  ||||||||||||||||||||||||||||||||||||||||||||||||||||||||||||
Sbjct  1379442   TGATAGGCGATCCAAAACAGGCAATTTATGGCTTTAGAGGTGGAGATATCTTCACATATC  1379501

Query  8341      TAAAAGCTAAAGATAGTTGTCCAGAAGAAAATCAATGGAGTATGGACACCAACTGGCGTT  8400
                  ||||||||||||||||||||||||||||||||||||||||||||||||||||||||||||
Sbjct  1379502   TAAAAGCTAAAGATAGTTGTCCAGAAGAAAATCAATGGAGTATGGACACCAACTGGCGTT  1379561

Query  8401      CAACTGGAGAGATGATAAAAGCTTATAATAGACTTTTTTATAAACAAGATTATCAGTCAG  8460
                  ||||||||||||||||||||||||||||||||||||||||||||||||||||||||||||
Sbjct  1379562   CAACTGGAGAGATGATAAAAGCTTATAATAGACTTTTTTATAAACAAGATTATCAGTCAG  1379621

Query  8461      AAGAAGAGGGGCAAATTGGAACTAATATTTTTAGTGATGGTATTGGATATCAGCTTGTCA  8520
```

```
                  ||||||||||||||||||||||||||||||||||||||||||||||||||||||||||||
Sbjct  1379622  AAGAAGAGGGGCAAATTGGAACTAATATTTTTAGTGATGGTATTGGATATCAGCTTGTCA  1379681

Query  8521     AAGCTTCACCGGATGCTGATaaaaaaaCAAAAGATTTTGACGATAATTTTAAACCTATTA  8580
                ||||||||||||||||||||||||||||||||||||||||||||||||||||||||||||
Sbjct  1379682  AAGCTTCACCGGATGCTGATAAAAAAACAAAAGATTTTGACGATAATTTTAAACCTATTA  1379741

Query  8581     ATTATTTCTATTATGAAGTAGCCGAAGATGATAATAAAAGTGATATTGATACGAATTTAG  8640
                ||||||||||||||||||||||||||||||||||||||||||||||||||||||||||||
Sbjct  1379742  ATTATTTCTATTATGAAGTAGCCGAAGATGATAATAAAAGTGATATTGATACGAATTTAG  1379801

Query  8641     CATTATGGACAGTAAATGAAATTACTAGATTACTAAATACACAAAAAGTAGCAGAAAATG  8700
                ||||||||||||||||||||||||||||||||||||||||||||||||||||||||||||
Sbjct  1379802  CATTATGGACAGTAAATGAAATTACTAGATTACTAAATACACAAAAAGTAGCAGAAAATG  1379861

Query  8701     ATATAGCTATTTTGGTAGAAAATGGCAAACAGGCAAAGATTATCCAACAAGCTTTGCAAG  8760
                ||||||||||||||||||||||||||||||||||||||||||||||||||||||||||||
Sbjct  1379862  ATATAGCTATTTTGGTAGAAAATGGCAAACAGGCAAAGATTATCCAACAAGCTTTGCAAG  1379921

Query  8761     CTAAAAATCTAAGCTCAGTATACCTAAGCCAGCGCGATAATGTCTATCACAGTCAAGAGG  8820
                ||||||||||||||||||||||||||||||||||||||||||||||||||||||||||||
Sbjct  1379922  CTAAAAATCTAAGCTCAGTATACCTAAGCCAGCGCGATAATGTCTATCACAGTCAAGAGG  1379981

Query  8821     CTAAAGAGATCTTAGCTCTTATGGAGGGTATAAATGATCTTGAAAATAAATCTATGTTGA  8880
                ||||||||||||||||||||||||||||||||||||||||||||||||||||||||||||
Sbjct  1379982  CTAAAGAGATCTTAGCTCTTATGGAGGGTATAAATGATCTTGAAAATAAATCTATGTTGA  1380041

Query  8881     AAAGGGCATTATCGACTAGTTTACTCGGTGGTAGAGCTGATAAGTTTATTAGCTATATTG  8940
                ||||||||||||||||||||||||||||||||||||||||||||||||||||||||||||
Sbjct  1380042  AAAGGGCATTATCGACTAGTTTACTCGGTGGTAGAGCTGATAAGTTTATTAGCTATATTG  1380101

Query  8941     ATGAAAATGATGTCTCAGCGTGGGATGATGAGATAGAAAAAGCCAAATCTCTAAGGCAAC  9000
                ||||||||||||||||||||||||||||||||||||||||||||||||||||||||||||
Sbjct  1380102  ATGAAAATGATGTCTCAGCGTGGGATGATGAGATAGAAAAAGCCAAATCTCTAAGGCAAC  1380161

Query  9001     AATGGCATAAATATGGATTTATGGCATTTATTATGCAGATTATACATGAGAATTTCACTC  9060
                ||||||||||||||||||||||||||||||||||||||||||||||||||||||||||||
Sbjct  1380162  AATGGCATAAATATGGATTTATGGCATTTATTATGCAGATTATACATGAGAATTTCACTC  1380221

Query  9061     AAAAAGCAGATTCAAAAGAGCGTATTATTACAAATATTCTTCATTTAGCAGAACTTATTA  9120
                ||||||||||||||||||||||||||||||||||||||||||||||||||||||||||||
Sbjct  1380222  AAAAAGCAGATTCAAAAGAGCGTATTATTACAAATATTCTTCATTTAGCAGAACTTATTA  1380281

Query  9121     AAGTGGCTGAGAATAAATATAAACACCCTAATCAACTCATTAAATGGTATCGCCACCAAC  9180
                ||||||||||||||||||||||||||||||||||||||||||||||||||||||||||||
Sbjct  1380282  AAGTGGCTGAGAATAAATATAAACACCCTAATCAACTCATTAAATGGTATCGCCACCAAC  1380341

Query  9181     TCAACAATACAGCTACAAGTGAGGGTGAACTTAGATTAGAAAGTGATGACAACTTGATTA  9240
```

```
                  ||||||||||||||||||||||||||||||||||||||||||||||||||||||||||||
Sbjct  1380342   TCAACAATACAGCTACAAGTGAGGGTGAACTTAGATTAGAAAGTGATGACAACTTGATTA  1380401

Query  9241      AGATAATTACAATCCATGGATCAAAAGGACTTGAGTATTCAGTTGTGTTTATCCCGTTTG  9300
                 ||||||||||||||||||||||||||||||||||||||||||||||||||||||||||||
Sbjct  1380402   AGATAATTACAATCCATGGATCAAAAGGACTTGAGTATTCAGTTGTGTTTATCCCGTTTG  1380461

Query  9301      CTTGCTATGCAAGCACAAAGAAGTTTGAGACAAGTAATTTTACTAAATATTATGATGGTG  9360
                 ||||||||||||||||||||||||||||||||||||||||||||||||||||||||||||
Sbjct  1380462   CTTGCTATGCAAGCACAAAGAAGTTTGAGACAAGTAATTTTACTAAATATTATGATGGTG  1380521

Query  9361      ACCTAAAACAAACTGTCTACAAAATTGGCAAAGATGATAGTGTGAAACAGCAAGCTGATA  9420
                 ||||||||||||||||||||||||||||||||||||||||||||||||||||||||||||
Sbjct  1380522   ACCTAAAACAAACTGTCTACAAAATTGGCAAAGATGATAGTGTGAAACAGCAAGCTGATA  1380581

Query  9421      AAGAGGTTATAGAAGAATTAATGCGTCTATTTTATGTAGCTGTTACGCGTGCAGAGCATA  9480
                 ||||||||||||||||||||||||||||||||||||||||||||||||||||||||||||
Sbjct  1380582   AAGAGGTTATAGAAGAATTAATGCGTCTATTTTATGTAGCTGTTACGCGTGCAGAGCATA  1380641

Query  9481      GATGTTATATTGGGGTTGCAAAATATAATAATAGCGAAAAGTCACCATTAGCAAGATTCT  9540
                 ||||||||||||||||||||||||||||||||||||||||||||||||||||||||||||
Sbjct  1380642   GATGTTATATTGGGGTTGCAAAATATAATAATAGCGAAAAGTCACCATTAGCAAGATTCT  1380701

Query  9541      TAGGTTATCAAAAAGATGATGATTGGTTAGAGAAGATCCAAAGCATAACAGCAAATCCTG  9600
                 ||||||||||||||||||||||||||||||||||||||||||||||||||||||||||||
Sbjct  1380702   TAGGTTATCAAAAAGATGATGATTGGTTAGAGAAGATCCAAAGCATAACAGCAAATCCTG  1380761

Query  9601      CTAATCAAAGTCTACTGATAAATATTGCAGATATGCGGGAATTTAGTTTAAAAAATTTAT  9660
                 ||||||||||||||||||||||||||||||||||||||||||||||||||||||||||||
Sbjct  1380762   CTAATCAAAGTCTACTGATAAATATTGCAGATATGCGGGAATTTAGTTTAAAAAATTTAT  1380821

Query  9661      CTAGCAAAGATAAAAATTCTGATTATGATACTCTCAAAGCAAATTATATTAGTAAACTAG  9720
                 ||||||||||||||||||||||||||||||||||||||||||||||||||||||||||||
Sbjct  1380822   CTAGCAAAGATAAAAATTCTGATTATGATACTCTCAAAGCAAATTATATTAGTAAACTAG  1380881

Query  9721      AAAATGATAGTTGGGAGATGTTATCTTTCTCAAAAATATCAAAGTCAAAAGTTCAAAATA  9780
                 ||||||||||||||||||||||||||||||||||||||||||||||||||||||||||||
Sbjct  1380882   AAAATGATAGTTGGGAGATGTTATCTTTCTCAAAAATATCAAAGTCAAAAGTTCAAAATA  1380941

Query  9781      CTGCATTAGAAAAAGAAATTGACGAGGCAGAAGATGATAAAACTCAAGCTTCAGACAGAA  9840
                 ||||||||||||||||||||||||||||||||||||||||||||||||||||||||||||
Sbjct  1380942   CTGCATTAGAAAAAGAAATTGACGAGGCAGAAGATGATAAAACTCAAGCTTCAGACAGAA  1381001

Query  9841      AGCTAGCATTTAGATTTACAGCTTCAAAAGGAGCTGATATGGGTAATATTCTGCATAATG  9900
                 ||||||||||||||||||||||||||||||||||||||||||||||||||||||||||||
Sbjct  1381002   AGCTAGCATTTAGATTTACAGCTTCAAAAGGAGCTGATATGGGTAATATTCTGCATAATG  1381061

Query  9901      TTTTAGAGCATACAGATTTTAGTTTAGGTGAGATAGATGGTAATCTTTTACAAGAACAAA  9960
```

```
                ||||||||||||||||||||||||||||||||||||||||||||||||||||||||||||
Sbjct  1381062  TTTTAGAGCATACAGATTTAGTTTAGGTGAGATAGATGGTAATCTTTTACAAGAACAAA  1381121

Query  9961     TGGATAGATATAAAGTAGTTGCTGCAGAAGACTTCGATAATCTTAAATTATGGTTGGAAG  10020
                ||||||||||||||||||||||||||||||||||||||||||||||||||||||||||||
Sbjct  1381122  TGGATAGATATAAAGTAGTTGCTGCAGAAGACTTCGATAATCTTAAATTATGGTTGGAAG  1381181

Query  10021    AATGTTTAGAAGCACATATACCTTATATAGATACCAGTTTTTCTAGCTTAAATGAGAGTA  10080
                ||||||||||||||||||||||||||||||||||||||||||||||||||||||||||||
Sbjct  1381182  AATGTTTAGAAGCACATATACCTTATATAGATACCAGTTTTTCTAGCTTAAATGAGAGTA  1381241

Query  10081    TTCAATATGGTTTATTTGATATCAAGGATAAAGGCTTTTGCTTAAAAACTATCCCAAATT  10140
                ||||||||||||||||||||||||||||||||||||||||||||||||||||||||||||
Sbjct  1381242  TTCAATATGGTTTATTTGATATCAAGGATAAAGGCTTTTGCTTAAAAACTATCCCAAATT  1381301

Query  10141    CGAAAACTCTAAAAGAGGCGGAGTTTTATTTTCCTGTTAAAAATGAGAATTTGTATAAAA  10200
                ||||||||||||||||||||||||||||||||||||||||||||||||||||||||||||
Sbjct  1381302  CGAAAACTCTAAAAGAGGCGGAGTTTTATTTTCCTGTTAAAAATGAGAATTTGTATAAAA  1381361

Query  10201    CAAATATATTAGAAATCTTGCAAGAGTATAGAGAGCTTACAAAACTGAAGACATATAGTC  10260
                ||||||||||||||||||||||||||||||||||||||||||||||||||||||||||||
Sbjct  1381362  CAAATATATTAGAAATCTTGCAAGAGTATAGAGAGCTTACAAAACTGAAGACATATAGTC  1381421

Query  10261    ATTCCTACGTAGGTGGGAATCTTCCAACTAACTACCAAGATCTAAGAGATACCCGCCTCC  10320
                ||||||||||||||||||||||||||||||||||||||||||||||||||||||||||||
Sbjct  1381422  ATTCCTACGTAGGTGGGAATCTTCCAACTAACTACCAAGATCTAAGAGATACCCGCCTCC  1381481

Query  10321    GCGAGTATGGCAATTTAACTAATGTTTCGAATCAAAAAATTTTTGGTATGCTTCATGGTT  10380
                ||||||||||||||||||||||||||||||||||||||||||||||||||||||||||||
Sbjct  1381482  GCGAGTATGGCAATTTAACTAATGTTTCGAATCAAAAAATTTTTGGTATGCTTCATGGTT  1381541

Query  10381    TTATAGATCTTATTTTTGAGTATGAGGGTAAATTTTATGTTGCTGATTATAAATCAAATT  10440
                ||||||||||||||||||||||||||||||||||||||||||||||||||||||||||||
Sbjct  1381542  TTATAGATCTTATTTTTGAGTATGAGGGTAAATTTTATGTTGCTGATTATAAATCAAATT  1381601

Query  10441    ATCTAGGTGATACTTTAGAAGATTATAATCAGCAGGCGATGCAAGAGAAAAATCAAAGTA  10500
                ||||||||||||||||||||||||||||||||||||||||||||||||||||||||||||
Sbjct  1381602  ATCTAGGTGATACTTTAGAAGATTATAATCAGCAGGCGATGCAAGAGAAAAATCAAAGTA  1381661

Query  10501    GCTTTTATGATTTACAGTATCTAATATATAGCGTTGCCTTGGATAAGTACCTGCGACAAA  10560
                ||||||||||||||||||||||||||||||||||||||||||||||||||||||||||||
Sbjct  1381662  GCTTTTATGATTTACAGTATCTAATATATAGCGTTGCCTTGGATAAGTACCTGCGACAAA  1381721

Query  10561    ACATTGAAAGTTATAATTATGAAAAGCATTTTGGCGGAGTATATTATTTTTATCTTAGAG  10620
                ||||||||||||||||||||||||||||||||||||||||||||||||||||||||||||
Sbjct  1381722  ACATTGAAAGTTATAATTATGAAAAGCATTTTGGCGGAGTATATTATTTTTATCTTAGAG  1381781

Query  10621    GTATGAAAGATGGTTATGGAGTTTATCGAGCTAGACCAAATTTAGAGATTATCAACAAAC  10680
```

```
                ||||||||||||||||||||||||||||||||||||||||||||||||||||||||||||
Sbjct  1381782  GTATGAAAGATGGTTATGGAGTTTATCGAGCTAGACCAAATTTAGAGATTATCAACAAAC  1381841

Query  10681    TTGCTAGTTTATTTAATGGAGATGATAATGTATAAAACTTTCCATAAAGCTTGTGAGCAA  10740
                ||||||||||||||||||||||||||||||||||||||||||||||||||||||||||||
Sbjct  1381842  TTGCTAGTTTATTTAATGGAGATGATAATGTATAAAACTTTCCATAAAGCTTGTGAGCAA  1381901

Query  10741    TTAGCAGATATTAGAGCCATAGATTTTTTCTTTGCTAAAGAGGTGTTTGATTTAGTCAAA  10800
                ||||||||||||||||||||||||||||||||||||||||||||||||||||||||||||
Sbjct  1381902  TTAGCAGATATTAGAGCCATAGATTTTTTCTTTGCTAAAGAGGTGTTTGATTTAGTCAAA  1381961

Query  10801    AAGACTACTCCGTCTATG  10818
                ||||||||||||||||||
Sbjct  1381962  AAGACTACTCCGTCTATG  1381979
```

Range 2: 1382583 to 1384489 GenBankGraphicsNext MatchPrevious MatchFirst Match

**Alignment statistics for match #2**

| Score | Expect | Identities | Gaps | St |
|---|---|---|---|---|
| 3522 bits(1907) | 0.0 | 1907/1907(100%) | 0/1907(0%) | Pl |

```
Query  10831    GCTCAGAGAATGACAGAGTCATTAAAAAATACTCTAGCAACTGTCATTCCGTCAAGCTTG  10890
                ||||||||||||||||||||||||||||||||||||||||||||||||||||||||||||
Sbjct  1382583  GCTCAGAGAATGACAGAGTCATTAAAAAATACTCTAGCAACTGTCATTCCGTCAAGCTTG  1382642

Query  10891    ACCACAGAATCTACTATACaaaaaaaTGATATCTTTAAACAAGTTCAGGATGAACTTAAT  10950
                ||||||||||||||||||||||||||||||||||||||||||||||||||||||||||||
Sbjct  1382643  ACCACAGAATCTACTATACAAAAAAATGATATCTTTAAACAAGTTCAGGATGAACTTAAT  1382702

Query  10951    AGTGTTGCTCAGTATCTTAATAATCTAGAAGCACAAACTATACATAGATTTTTAGGCTTA  11010
                ||||||||||||||||||||||||||||||||||||||||||||||||||||||||||||
Sbjct  1382703  AGTGTTGCTCAGTATCTTAATAATCTAGAAGCACAAACTATACATAGATTTTTAGGCTTA  1382762

Query  11011    AGACCAAACTCTACTTATGTTAAATATAACCAACAATCTAAAGCTCCATTCGATGTTATT  11070
                ||||||||||||||||||||||||||||||||||||||||||||||||||||||||||||
Sbjct  1382763  AGACCAAACTCTACTTATGTTAAATATAACCAACAATCTAAAGCTCCATTCGATGTTATT  1382822

Query  11071    ATTGTTGATGAAGCTTCGATGCTTGATATGAATATTTTTATCAAGTTAATTAGAGCTGTA  11130
                ||||||||||||||||||||||||||||||||||||||||||||||||||||||||||||
Sbjct  1382823  ATTGTTGATGAAGCTTCGATGCTTGATATGAATATTTTTATCAAGTTAATTAGAGCTGTA  1382882

Query  11131    GCAGATAATACTAAGTTGATATTAATAGGTGATACTAATCAGTTACCATCTGTTGAGGCT  11190
                ||||||||||||||||||||||||||||||||||||||||||||||||||||||||||||
Sbjct  1382883  GCAGATAATACTAAGTTGATATTAATAGGTGATACTAATCAGTTACCATCTGTTGAGGCT  1382942

Query  11191    GGGAGTCTTTTAGCAAACTTTACACATGATAAATATGGTAGCATTACGCCATATACAACA  11250
                ||||||||||||||||||||||||||||||||||||||||||||||||||||||||||||
```

```
Sbjct  1382943  GGGAGTCTTTTAGCAAACTTTACACATGATAAATATGGTAGCATTACGCCATATACAACA  1383002

Query  11251    TTACTTATCAAAAACTATCGTTCACAACAATATATAAATAATCTTGCAGCCTCAGTCCTA  11310
                ||||||||||||||||||||||||||||||||||||||||||||||||||||||||||||
Sbjct  1383003  TTACTTATCAAAAACTATCGTTCACAACAATATATAAATAATCTTGCAGCCTCAGTCCTA  1383062

Query  11311    AAAGGCGATGTTAATATTGATAGTCAACAAAATGAAAATATTAAATTTCATAGTCTAAGA  11370
                ||||||||||||||||||||||||||||||||||||||||||||||||||||||||||||
Sbjct  1383063  AAAGGCGATGTTAATATTGATAGTCAACAAAATGAAAATATTAAATTTCATAGTCTAAGA  1383122

Query  11371    AATTTAGATATTTATTTAAGAGAGTATGCAAAGAGATATTCACAGCTTGAAAAGTGCCGT  11430
                ||||||||||||||||||||||||||||||||||||||||||||||||||||||||||||
Sbjct  1383123  AATTTAGATATTTATTTAAGAGAGTATGCAAAGAGATATTCACAGCTTGAAAAGTGCCGT  1383182

Query  11431    GACTACAAAGAGGCTCTTACAGAGCTGAATAAATTTAGAATACTTGTGGCTAACAAAAAC  11490
                ||||||||||||||||||||||||||||||||||||||||||||||||||||||||||||
Sbjct  1383183  GACTACAAAGAGGCTCTTACAGAGCTGAATAAATTTAGAATACTTGTGGCTAACAAAAAC  1383242

Query  11491    CTAGAAATTGGTACAGATAAGTTAAATCAGAAAATTGAGAAGTTTATGTCTAAGCCTATA  11550
                ||||||||||||||||||||||||||||||||||||||||||||||||||||||||||||
Sbjct  1383243  CTAGAAATTGGTACAGATAAGTTAAATCAGAAAATTGAGAAGTTTATGTCTAAGCCTATA  1383302

Query  11551    GATTCTAACTATAAGGGTAAACCAATTATGATAACTCAAAATAGTTATTCATTAGGGTTA  11610
                ||||||||||||||||||||||||||||||||||||||||||||||||||||||||||||
Sbjct  1383303  GATTCTAACTATAAGGGTAAACCAATTATGATAACTCAAAATAGTTATTCATTAGGGTTA  1383362

Query  11611    TTTAATGGAGATGTAGGGATTATGTGGCCTGATGATACTGGTAAGCTCCGAGCATATTTT  11670
                ||||||||||||||||||||||||||||||||||||||||||||||||||||||||||||
Sbjct  1383363  TTTAATGGAGATGTAGGGATTATGTGGCCTGATGATACTGGTAAGCTCCGAGCATATTTT  1383422

Query  11671    GATGGTAAAGATGCTAAGGCATTTAGTCTAAATATGCTACCAAAATATGAAAGTGTTTAT  11730
                ||||||||||||||||||||||||||||||||||||||||||||||||||||||||||||
Sbjct  1383423  GATGGTAAAGATGCTAAGGCATTTAGTCTAAATATGCTACCAAAATATGAAAGTGTTTAT  1383482

Query  11731    GCGATGACAATTCATAAAACTCAAGGCTCAGAGTTTGATGAAATCGTGATTATTCTTCCA  11790
                ||||||||||||||||||||||||||||||||||||||||||||||||||||||||||||
Sbjct  1383483  GCGATGACAATTCATAAAACTCAAGGCTCAGAGTTTGATGAAATCGTGATTATTCTTCCA  1383542

Query  11791    GCAGAAGATAATGAAGCGCTAAGTAAACAACTTTTGTATACAGCTATTACTAGAGCAAAA  11850
                ||||||||||||||||||||||||||||||||||||||||||||||||||||||||||||
Sbjct  1383543  GCAGAAGATAATGAAGCGCTAAGTAAACAACTTTTGTATACAGCTATTACTAGAGCAAAA  1383602

Query  11851    TATAAATTAACGATTATATCTGAACAAAGTAGCTTAAGAGATATTGCTCAGAAAGATATT  11910
                ||||||||||||||||||||||||||||||||||||||||||||||||||||||||||||
Sbjct  1383603  TATAAATTAACGATTATATCTGAACAAAGTAGCTTAAGAGATATTGCTCAGAAAGATATT  1383662

Query  11911    AATAGAAATTCAAATATCAGTGAATTAGTTAGCTTGAGCACTCAATAACTTAGAAATTAC  11970
                ||||||||||||||||||||||||||||||||||||||||||||||||||||||||||||
```

```
Sbjct  1383663  AATAGAAATTCAAATATCAGTGAATTAGTTAGCTTGAGCACTCAATAACTTAGAAATTAC  1383722

Query  11971    TTATTCATTTGCCAGATTATTCTAAGTCCATGAAGTAATAAATCAGAGATGATTACATCA  12030
                ||||||||||||||||||||||||||||||||||||||||||||||||||||||||||||
Sbjct  1383723  TTATTCATTTGCCAGATTATTCTAAGTCCATGAAGTAATAAATCAGAGATGATTACATCA  1383782

Query  12031    AAATATTGTTCTTTCTCAAAAATATGAGCATAGCCTCCAGTTGCTATTAATATTGGTGGT  12090
                ||||||||||||||||||||||||||||||||||||||||||||||||||||||||||||
Sbjct  1383783  AAATATTGTTCTTTCTCAAAAATATGAGCATAGCCTCCAGTTGCTATTAATATTGGTGGT  1383842

Query  12091    TTATCTATGAAGTTTTCTTGAGATATTCGATTAATTATCTCTTTTATAGCACCTAATTGA  12150
                ||||||||||||||||||||||||||||||||||||||||||||||||||||||||||||
Sbjct  1383843  TTATCTATGAAGTTTTCTTGAGATATTCGATTAATTATCTCTTTTATAGCACCTAATTGA  1383902

Query  12151    CCATATATAAGACCCGATTGGATTTGAGAGATAGTTGTCTTACCTAACGCAGAACTAGGG  12210
                ||||||||||||||||||||||||||||||||||||||||||||||||||||||||||||
Sbjct  1383903  CCATATATAAGACCCGATTGGATTTGAGAGATAGTTGTCTTACCTAACGCAGAACTAGGG  1383962

Query  12211    TGAGAGATAGTTACATTAGATAGTTTTGCAGTTTTTTGTGATAAGCTCTCCATTGATAGG  12270
                ||||||||||||||||||||||||||||||||||||||||||||||||||||||||||||
Sbjct  1383963  TGAGAGATAGTTACATTAGATAGTTTTGCAGTTTTTTGTGATAAGCTCTCCATTGATAGG  1384022

Query  12271    TTAATTCCTGGTAGGATAGCACCGCCGATATAGGTTTTATTCTCTGAAATAGCACAAATA  12330
                ||||||||||||||||||||||||||||||||||||||||||||||||||||||||||||
Sbjct  1384023  TTAATTCCTGGTAGGATAGCACCGCCGATATAGGTTTTATTCTCTGAAATAGCACAAATA  1384082

Query  12331    GTTGTTGCTGTGCCAAAGTCAACTACAATAATATTTCTCGATGGAAAAAGAGAAATAGCT  12390
                ||||||||||||||||||||||||||||||||||||||||||||||||||||||||||||
Sbjct  1384083  GTTGTTGCTGTGCCAAAGTCAACTACAATAATATTTCTCGATGGAAAAAGAGAAATAGCT  1384142

Query  12391    GCAACTGAGTTTGCAACACGATCTGCACCTAAATCTAGAGGATTTTTGATATCGAGTTTT  12450
                ||||||||||||||||||||||||||||||||||||||||||||||||||||||||||||
Sbjct  1384143  GCAACTGAGTTTGCAACACGATCTGCACCTAAATCTAGAGGATTTTTGATATCGAGTTTT  1384202

Query  12451    AATCCTGTTTTTACCCCAGGTTTTAGCTCAAGCGGAGTTATACCTAAATACTTTTTGCAA  12510
                ||||||||||||||||||||||||||||||||||||||||||||||||||||||||||||
Sbjct  1384203  AATCCTGTTTTTACCCCAGGTTTTAGCTCAAGCGGAGTTATACCTAAATACTTTTTGCAA  1384262

Query  12511    GCGGAATTGACAGAATACTCTAGATGAAGAACAACAGATGAAAGTACAACTGCTTCAATA  12570
                ||||||||||||||||||||||||||||||||||||||||||||||||||||||||||||
Sbjct  1384263  GCGGAATTGACAGAATACTCTAGATGAAGAACAACAGATGAAAGTACAACTGCTTCAATA  1384322

Query  12571    TCTTCGATATCAAGCTTTTTCCTCTCAAAGAACGATAGCAGAAATATACCTAGCGTATCA  12630
                ||||||||||||||||||||||||||||||||||||||||||||||||||||||||||||
Sbjct  1384323  TCTTCGATATCAAGCTTTTTCCTCTCAAAGAACGATAGCAGAAATATACCTAGCGTATCA  1384382

Query  12631    GAAGTACACGGAGTAGTAGAAGGGTAGCGAAAGTTATGCTTAATTTGATCTCCTACAAAT  12690
                ||||||||||||||||||||||||||||||||||||||||||||||||||||||||||||
```

```
Sbjct  1384383  GAAGTACACGGAGTAGTAGAAGGGTAGCGAAAGTTATGCTTAATTTGATCTCCTACAAAT  1384442

Query  12691    ACTCCACCAAAAATATGTGAGTTACCAATATCTATACATACTATCAT  12737
                |||||||||||||||||||||||||||||||||||||||||||||||
Sbjct  1384443  ACTCCACCAAAAATATGTGAGTTACCAATATCTATACATACTATCAT  1384489
```
